# Transcriptomic Analysis of Rice (*Oryza sativa*) Developing Embryos Using the RNA-Seq Technique

**DOI:** 10.1371/journal.pone.0030646

**Published:** 2012-02-08

**Authors:** Hong Xu, Yi Gao, Jianbo Wang

**Affiliations:** State Key Laboratory of Hybrid Rice, College of Life Sciences, Wuhan University, Wuhan, China; University of Toronto, Canada

## Abstract

Rice (*Oryza sativa*) is an excellent model monocot with a known genome sequence for studying embryogenesis. Here we report the transcriptome profiling analysis of rice developing embryos using RNA-Seq as an attempt to gain insight into the molecular and cellular events associated with rice embryogenesis. RNA-Seq analysis generated 17,755,890 sequence reads aligned with 27,190 genes, which provided abundant data for the analysis of rice embryogenesis. A total of 23,971, 23,732, and 23,592 genes were identified from embryos at three developmental stages (3–5, 7, and 14 DAP), while an analysis between stages allowed the identification of a subset of stage-specific genes. The number of genes expressed stage-specifically was 1,131, 1,443, and 1,223, respectively. In addition, we investigated transcriptomic changes during rice embryogenesis based on our RNA-Seq data. A total of 1,011 differentially expressed genes (DEGs) (log_2_Ratio ≥1, FDR ≤0.001) were identified; thus, the transcriptome of the developing rice embryos changed considerably. A total of 672 genes with significant changes in expression were detected between 3–5 and 7 DAP; 504 DEGs were identified between 7 and 14 DAP. A large number of genes related to metabolism, transcriptional regulation, nucleic acid replication/processing, and signal transduction were expressed predominantly in the early and middle stages of embryogenesis. Protein biosynthesis-related genes accumulated predominantly in embryos at the middle stage. Genes for starch/sucrose metabolism and protein modification were highly expressed in the middle and late stages of embryogenesis. In addition, we found that many transcription factor families may play important roles at different developmental stages, not only in embryo initiation but also in other developmental processes. These results will expand our understanding of the complex molecular and cellular events in rice embryogenesis and provide a foundation for future studies on embryo development in rice and other cereal crops.

## Introduction

Rice (*Oryza sativa*), which belongs to the grass family (Poaceae), together with barley, wheat, maize, and sorghum, are important cereal crops that support the global food supply. Rice offers various advantages as an experimental plant compared with other monocot species, including a small genome size and known genome sequence [1]. Patterning during embryogenesis begins with asymmetric zygote cell division that produces a small apical cell that ultimately becomes the embryo and a large basal cell that develops into the suspensor. The classification of gene expression patterns associated with specific stages of embryo development and a functional understanding of the encoded genes is critical for comprehending the molecular and biochemical events associated with embryogenesis. Although embryo development is a major subject in plant growth and development research, there is still a long way to go in order to understand the mechanism of this developmental process. Given the availability of a genomic sequence database, rice is an excellent model monocot for studying embryogenesis.

Most knowledge concerning the genetic program activated during embryogenesis in higher plants comes from the study of mutants. Mutations affecting embryonic development have been identified in several plant species. For example, the role of *AGL23* gene in *Arabidopsis* embryo development [2], auxin-induced developmental patterns in *Brassica juncea* embryos [3], and the essential function of *Orysa;CycB1;1* gene in rice embryo enlargement [4] were identified. Analyses of these mutants have led to the identification and characterization of several genes with key roles in plant embryonic development. However, mutagenesis alone cannot be used to identify all of the genes that are potentially involved in a biological process.

In the past few years, several differential screening techniques (e.g., differential display, subtraction libraries, and differential hybridization) have made it possible to characterize genes that are differentially expressed during embryogenesis [5], [6]. Recently, methods such as serial analysis of gene expression (SAGE) and microarray analyses have allowed us to visualize global changes in transcript abundance in a spatial, temporal, or conditional way [7], [8]. Genome-wide transcription profiling is an important and powerful tool leading to the generation of testable hypotheses for novel processes not yet characterized at the molecular level. Its usefulness has been demonstrated in investigations of transcriptional programs occurring in a variety of developmental processes, including flower development, embryo development, seed development, defense responses to pathogens, and the response to wounding [9]–[11]. For example, the results of a genome-wide microarray analysis provided an outline of gene expression patterns and metabolic network models of embryogenesis in *Arabidopsis* [9]. Microarray analyses of the rice transcriptome encompassing different cell types, tissues and organs, specific stages of growth and development [12], [13] have generated a large amount of information that provides initial clues for understanding the function of genes based on their time, place and level of expression in the plant.

More recently developed RNA deep-sequencing technologies, such as digital gene expression (DGE) [14] and Solexa/Illumina RNA-Seq [15], will dramatically change the methods used to identify embryogenesis-related genes in plants because these technologies facilitate investigations of the functional complexity of transcriptomes. RNA-Seq refers to whole-transcriptome shotgun sequencing, wherein mRNA or cDNA is mechanically fragmented, resulting in overlapping short fragments that cover the entire transcriptome. Recent studies have shown that massively parallel sequencing technology is more sensitive for low-expressed transcripts [16], [17] than traditional SAGE and microarray hybridizations [7], [8].

The expression level of virtually all genes in a sample is measured by counting the number of individual mRNA molecules produced from each gene. RNA-Seq is more suitable and affordable for comparative gene expression studies because it verifies direct transcript profiling without compromise and potential bias, thus allowing for more sensitive and accurate profiling of the transcriptome that more closely resembles the biology of the cell [15], [17]. This technology has been used in transcriptome profiling studies for various organisms, including maize, rice, and soybean [18]–[21]. For example, a genome-wide survey of imprinted genes in rice seeds revealed imprinting primarily occurs in the endosperm, and one of these genes was also imprinted in the embryo [21]. However, RNA-Seq technology has not been used to analyze embryogenesis in rice.

Here, we used deep RNA sequencing (the Illumina RNA-Seq method) to rapidly identify and analyze the rice (*O. sativa*) transcriptome during embryo development in a cost-effective manner. We report a comprehensive analysis of transcriptome dynamics that may serve as a gene expression profile blueprint for embryo development, which was used to investigate the regulation of gene expression during early embryo development events from 3 to 14 days after pollination (DAP). This included identifying genes expressed in a time-specific manner, defining clusters of genes showing similar patterns of temporal expression, and identifying stage- or transition-specific candidate genes for additional functional analyses. Four expression patterns (clusters) of differentially expressed genes (DEGs) were identified. The genes of the different expression clusters associated with different functional categories clearly indicate the molecular and cellular events involved in rice embryo development.

## Results

### Illumina sequencing and aligning to the reference genome

An immediate application of our transcriptome sequence data included gene expression profiling at different embryo developmental stages. The RNA-Seq method generates absolute information, rather than relative gene expression measurements; thus, it avoids many of the inherent limitations of microarray analysis. This method was used to analyze variations in gene expression during rice embryo development. We sequenced three cDNA libraries, R1 (3–5 DAP), R2 (7 DAP), and R3 (14 DAP), and generated 17,755,890 sequence reads, each of which was 42–50 bp in length, encompassing 2.34 Gb of sequence data (Table 1). Each stage was represented by approximately 6 million reads, a tag density sufficient for the quantitative analysis of gene expression. The sequence reads were aligned to the rice reference genome database using SOAPaligner/soap2 software (set to allow two base mismatches). Of the total reads, 83.28% matched either to a unique (77.53%) or to multiple (5.75%) genomic locations; the remaining 16.72% were unmatched (Table 1), because only reads aligning entirely inside exonic regions will be matched (reads from exon-exon junction regions will not match).

**Table 1:** Summary of read numbers based on the RNA-Seq data from rice developing embryos.

	*R1(3–5 DAP)*	*R2(7 DAP)*	*R3(14 DAP)*
Total reads	6,035,714	5,720,176	6,000,001
Mapped reads	4,995,740	4,777,902	5,013,883
	(82.77%)	(83.53%)	(83.56%)
Unique match	4,622,547	4,461,088	4,681,906
	(76.59%)	(77.99%)	(78.03%)
Multi-position match	373,193	316,814	331,977
	(6.18%)	(5.54%)	(5.53%)
Unmapped reads	1,039,974	942,274	986,117
	(17.23%)	(16.47%)	(16.44%)

### Global analysis of gene expression

One of the primary goals of RNA sequencing is to compare gene expression levels between samples. For robust conclusions about biological differences among samples, it is important to utilize biological replication. Here, we pooled biological replicates from multiple embryo clusters to obtain representative samples for deep sequencing analysis; specifically, we were interested in developing approaches to look at differences in expression among the three samples. The following analyses demonstrate methods for using RNA-Seq data to perform global analyses of transcriptome variation during rice embryo development.

**Figure 1:**
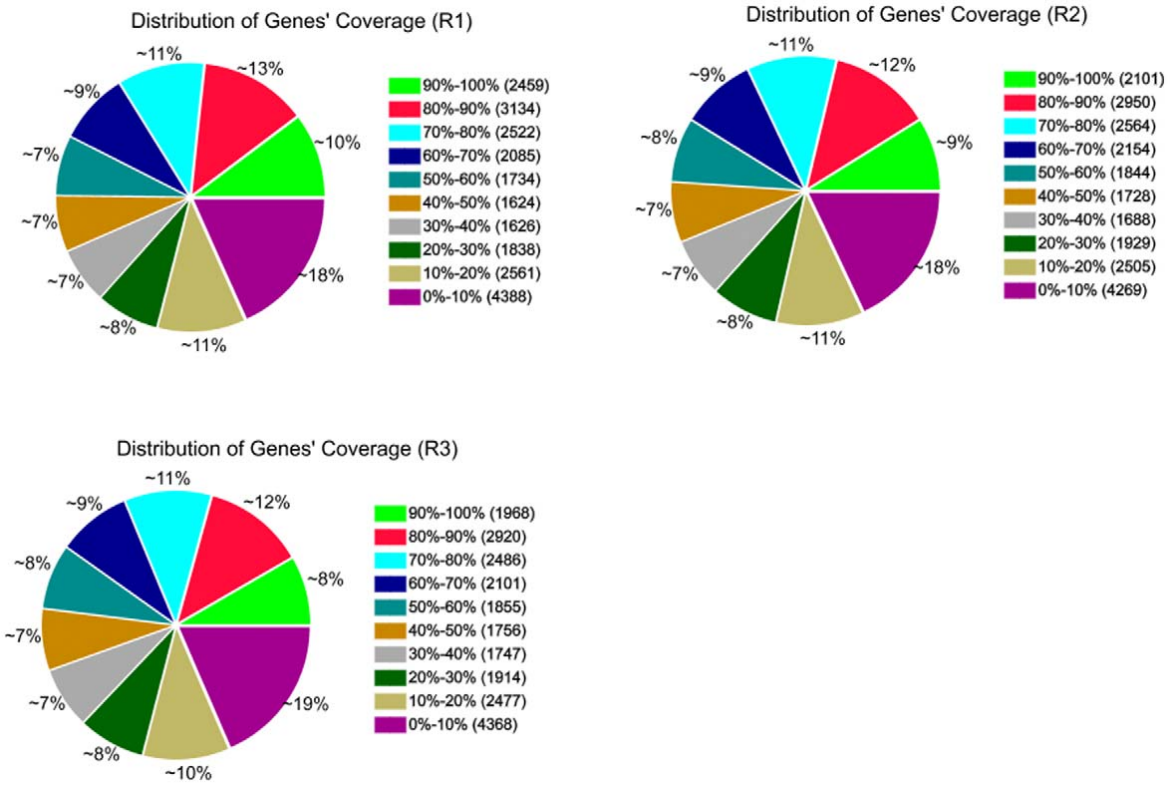
Percent of coverage representing the percentage of genes which expressed in each of the three stages mapped in the rice genome. R1, 3–5 DAP; R2, 7 DAP; R3, 14 DAP. Gene coverage is the percentage of a gene covered by reads. This value is equal to the ratio of the base number in a gene covered by unique mapping reads to the total bases number of that gene. The distribution of distinct tags over different tag abundance categories show similar patterns for all three RNA-Seq libraries. The similarity distribution has a comparable pattern with more than 20% of the sequences having a similarity >80%, while approximately 80% of the hits has a similar range.

ERANGE (version 4.0) (http://woldlab.caltech.edu/gitweb/) measures gene expression in reads per kilobase per million mapped sequence reads (RPKM), a normalized measure of read density that allows transcript levels to be compared both within and between samples. As ERANGE distributes multireads at similar loci in proportion to the number of unique reads recorded, we included in the analysis both unique reads and reads that occurred up to ten times to avoid undercounting genes with closely related paralogs. For mRNA expression, heterogeneity and redundancy are two significant characteristics. While the majority of mRNA is expressed at low levels, a small proportion of mRNA is highly expressed. Therefore, the distribution of tag expression was used to evaluate the normality of our RNA-Seq data. As shown in Figure 1, the distribution of distinct tags over different tag abundance categories showed similar patterns for all three RNA-Seq libraries. The similarity distribution had a comparable pattern with more than 20% of the sequences having a similarity >80%, while approximately 80% of the hits had a similar range (Figure 1).

**Figure 2:**
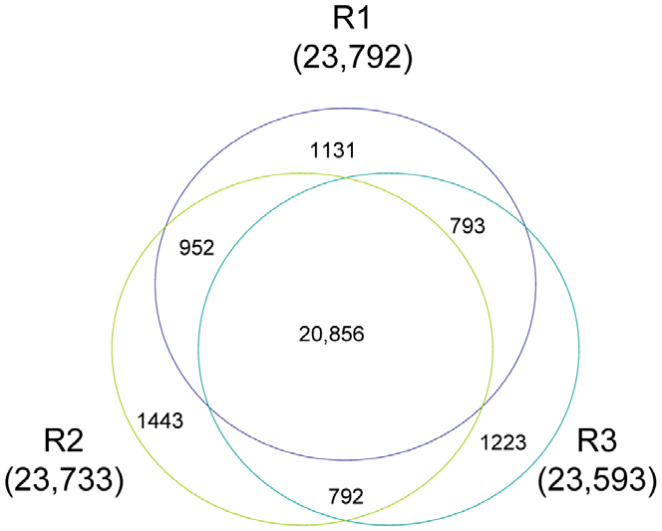
Venn diagram showing the genes expressed in each of the three stages of rice embryo development. R1, 3–5 DAP; R2, 7 DAP; R3, 14 DAP. Among these genes, 20,856 are expressed at all three developmental stages, 952 are co-expressed in R1 and R2, 792 are co-expressed in R2 and R3, and 793 are co-expressed in R1 and R3. The number of stage-specifically expressed genes is 1,131 (R1), 1,443 (R2), and 1,223 (R3), respectively.

A total of 23,971 (R1), 23,732 (R2), and 23,592 (R3) genes, ranging from 100 to ≥2,000 bp, were detected in the samples. As shown in Table 2, the proportion of sequences with matches to rice databases was higher among the longer assembled sequences. Specifically, a match efficiency of 38.99% was observed for sequences longer than 2,000 bp, whereas the match efficiency decreased to about 14.57% for those ranging from 500 to 1,000 bp, and to 4.56% for sequences between 100 to 500 bp (Table 2). The removal of partial overlapping sequences yielded 27,190 genes, providing abundant data for the analysis of rice embryo development. Their expression in the three developmental stages is summarized in Figure 2. A Venn diagram shows the distribution of expressed genes from R1 (3–5 DAP), R2 (7 DAP), and R3 (14 DAP). Among these genes, 20,856 were expressed at all three developmental stages, 952 were co-expressed in R1 and R2, 792 were co-expressed in R2 and R3, and 793 were co-expressed in R1 and R3. The number of stage-specifically expressed genes was 1,131 (R1), 1,443 (R2), and 1,223 (R3), respectively. Although there are 20,856 genes expressed at all three developmental stages, many of them are quantitatively regulated. While some of these genes had little variation across embryo development, which may be thought to fulfill housekeeping functions.

**Table 2:** Distribution of the gene sequences detected in rice developing embryo via RNA-Seq.

*Gene length (bp)*	*Total number*	*Percentage (%)*
100–500	1239	4.56
500–1000	3963	14.57
1000–1500	5608	20.63
1500–2000	5779	21.25
≥2000	10601	38.99
Total	27190	100

### Annotation of all detected genes expressed during rice embryo development

**Figure 3:**
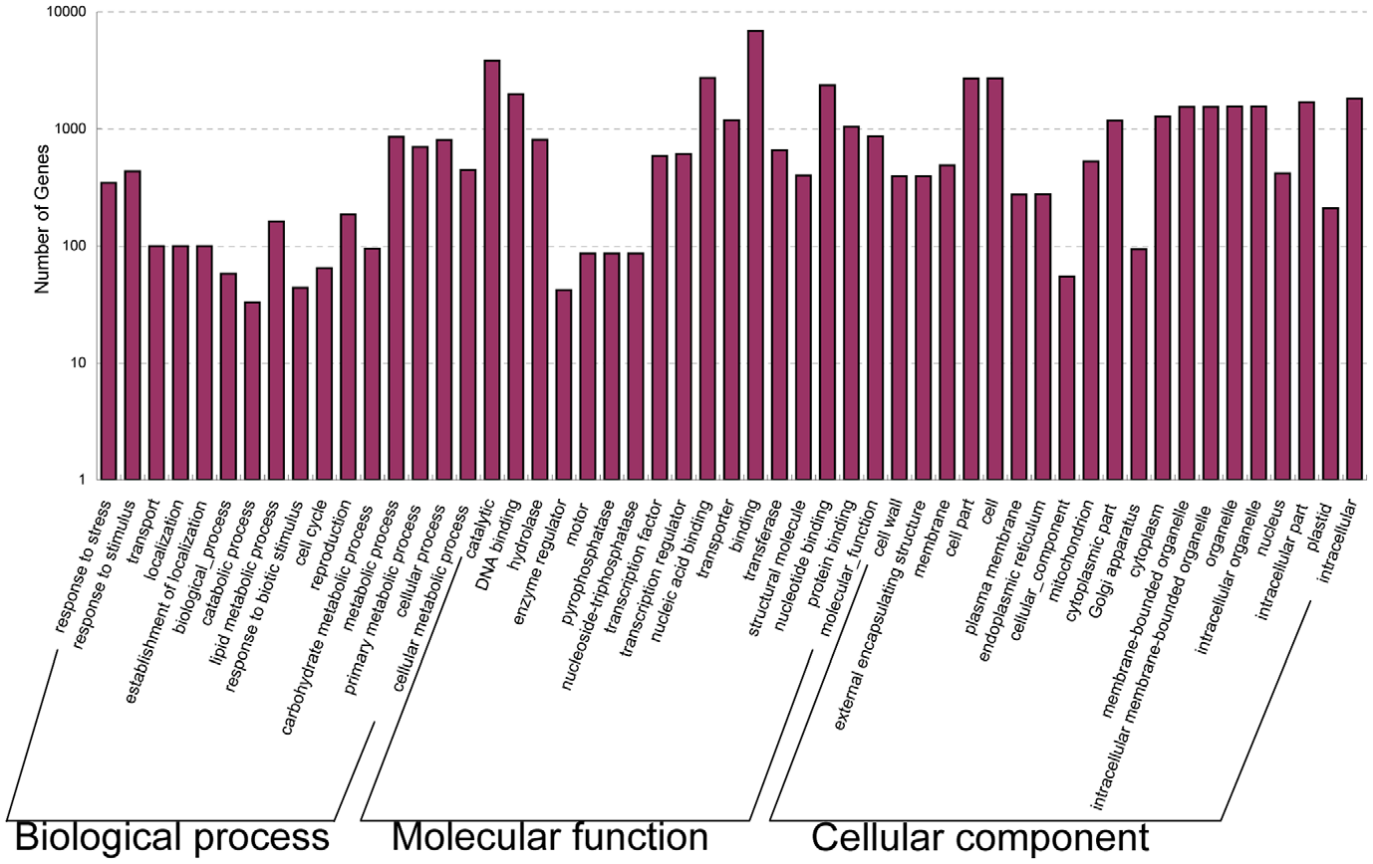
Histogram presentation of gene ontology (GO) classification. The results are summarized in three main categories: biological process, molecular function and cellular component. The y-axis indicates the number of genes in a category. In three main categories of GO classification, there are 16, 17, and 20 functional groups, respectively. Metabolic process (GO: 0008152), with 851 genes, are dominant in the main category of biological process. Binding (GO: 0005488) and cell part (GO: 0044464) consisted of 6892 and 2688 genes, are dominant in the main categories of molecular function and cellular component, respectively.

To facilitate the global analysis of gene expression, all predicted rice genes were assigned to different functional categories using Blast2GO (version 2.3.5) (http://www.blast2go.org/). The annotations were verified manually and integrated using gene ontology (GO) classification (http://www.geneontology.org). Of 27,190 detected genes, 18,307 were categorized into 53 functional groups based on sequence homology. In each of the three main categories (biological process, molecular function, and cellular component) of the GO classification, there were 16, 17, and 20 functional groups, respectively (Figure 3). Metabolic process (GO: 0008152), with 851 genes, were dominant in the main category of biological process. Binding (GO: 0005488) and cell part (GO: 0044464) consisted of 6892 and 2688 genes, were dominant in the main categories of molecular function and cellular component, respectively. We also noticed a high percentage of genes from functional groups of cellular process (GO: 0009987) with 803 genes, nucleic acid binding (GO: 0003676) with 2725 genes, and intracellular (GO: 0005622) consisted of 1809 genes in the three main categories, respectively (Figure 3).

To identify the biological pathways that are active in rice embryos, we mapped the detected genes to reference canonical pathways in the Kyoto Encyclopedia of Genes and Genomes (KEGG) (http://www.genome.ad.jp/kegg/) [22]. Of the total 27,190 detected genes, 21,258 were assigned to 164 KEGG pathways. Those pathways with the greatest representation by unique genes were for biosynthesis of secondary metabolites (1,938 members); inositol phosphate metabolism (1,061 members); valine, leucine, and isoleucine degradation (1,045 members); and propanoate metabolism (1,035 members). These annotations provide a valuable resource for investigating specific processes, functions, and pathways during rice embryo development.

### Changes in gene expression profiles among the different developmental stages

To obtain statistical confirmation of the differences in gene expression among the developmental stages, we compared the RPKM-derived read count using a likelihood ratio test [23]. To minimize false positives and negatives, we concluded that a statistical analysis was reliable when applied to genes with an RPKM value ≥2 (i.e., six mapped reads on 200 nt of mRNA) in at least one of the three stages. It should be noted that these statistical significances are based on expected sampling distributions. To determine which of the 27,190 genes were differentially expressed between the three developmental stages, we required a twofold or greater change in expression and false discovery rate (FDR) of 10^−3^ or less, which resulted in a set of 1,011 DEGs (Table S1).

**Figure 4:**
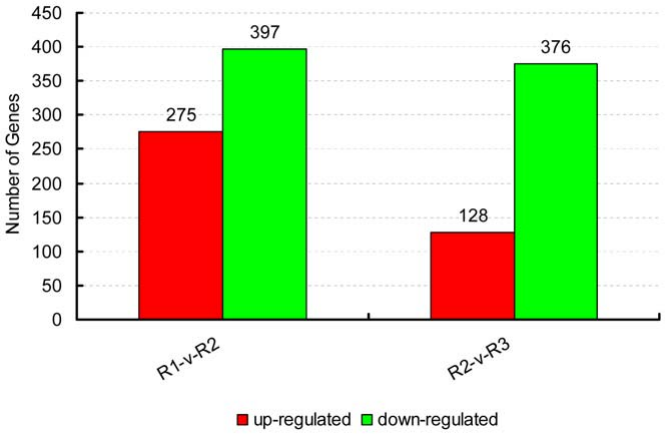
Changes in gene expression profile among the different developmental stages. The number of up-regulated and down-regulated genes between R1 and R2, R2 and R3 are summarized. Between the R1 (3 DAP) and R2 (7 DAP) rice embryo libraries, there are 275 genes up-regulated and 397 genes down-regulated, while there are 128 up-regulated genes and 376 down-regulated genes between the R2 (7 DAP) and R3 (14 DAP) rice embryo libraries.

To identify genes showing a significant change in expression during different developmental stages, the differentially expressed tags between two samples were identified using an algorithm developed by Audic *et al.* [24]. A total of 672 significantly changed genes were detected between the R1 (3 DAP) and R2 (7 DAP) rice embryo libraries, with 275 genes up-regulated and 397 genes down-regulated (Figure 4 and Table S2). Between the R2 (7 DAP) and R3 (14 DAP) rice embryo libraries, a total of 504 DEGs were detected, with 128 up-regulated genes and 376 down-regulated genes (Figure 4 and Table S3). This suggests that the differentiation of expressed genes between R1 (3 DAP) and R2 (7 DAP) is larger than that between R2 (7 DAP) and R3 (14 DAP).

### Functional analysis of DEGs based on RNA-Seq data

**Figure 5:**
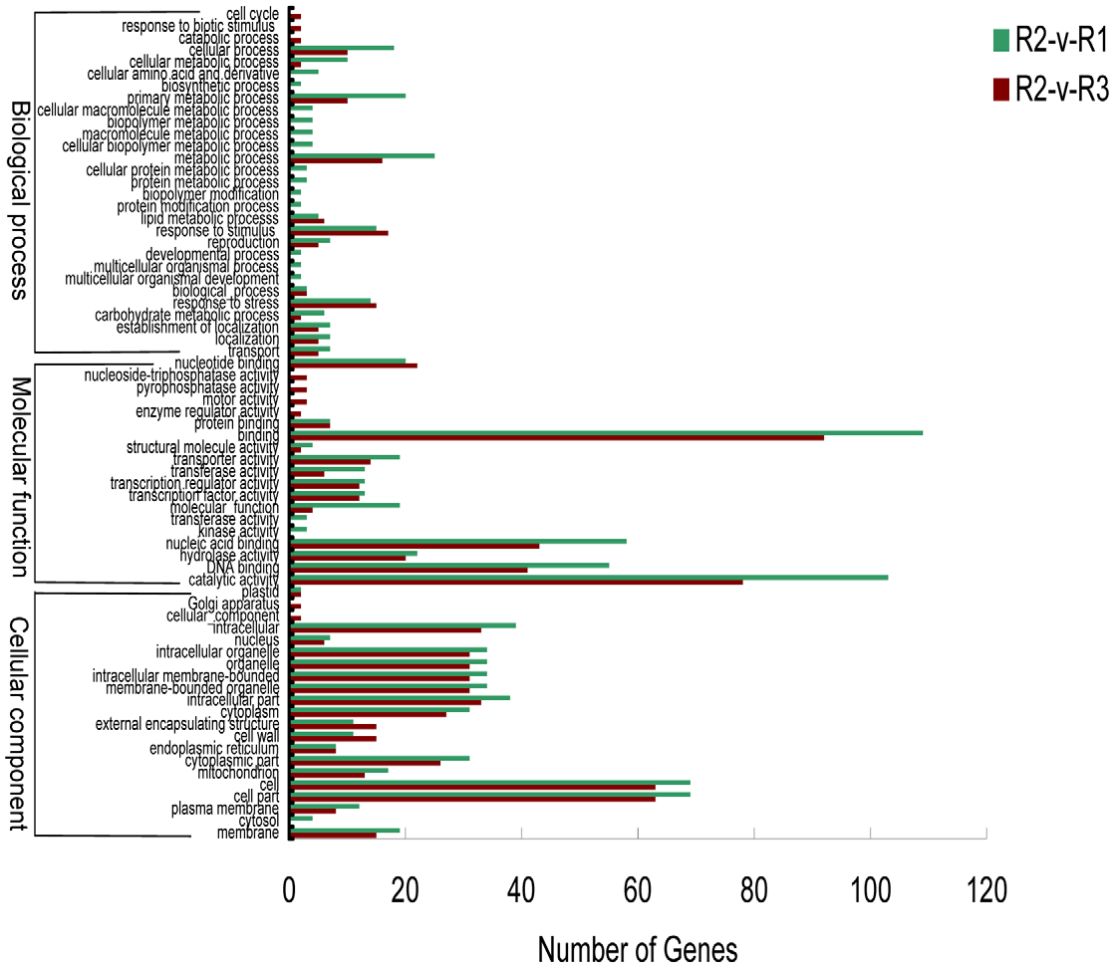
Functional analysis of differentially expressed genes (DEGs) based on RNA-Seq data. GO functional enrichment analysis of differentially expressed genes in R2-v-R1 and R2-v-R3. Based on sequence homology, 1,011 DEGs could be categorized into three main categories (cellular component, molecular function, and biological process), in which there are 14, 17, and 20 functional groups, respectively. Among these groups, the terms cell part (GO: 0044464), binding (GO: 0005488), and metabolic process (GO: 0008152) are dominant in each of the three main categories, respectively.

GO analyses were used to classify the functions of the DEGs during rice embryogenesis. Based on sequence homology, 1,011 DEGs could be categorized into 51 functional groups (Figure 5 and Table S4). In the three main categories (cellular component, molecular function, and biological process) of the GO classification, there were 14, 17, and 20 functional groups, respectively (Figure 5). Among these groups, the terms cell part (GO: 0044464), binding (GO: 0005488), and metabolic process (GO: 0008152) were dominant in each of the three main categories, respectively. We also noticed a high percentage of genes from functional groups of intracellular part (GO: 0044424), membrane-bounded organelle (GO: 0043227), cytoplasmic part (GO: 0044444), catalytic activity (GO: 0003824), nucleic acid binding (GO: 0003676), DNA binding (GO: 0003677), primary metabolic process (GO: 0044238), and response to stimulus (GO: 0050896) (Figure 5).

To further understand the functions of the DEGs, we mapped the genes to terms in the KEGG database (http://www.genome.ad.jp/kegg/) and compared this with the whole transcriptome background, with a view to search for genes involved in metabolic or signal transduction pathways that were significantly enriched. Among those genes with a KEGG pathway annotation, 325 DEGs were identified between the R1 and R2 libraries. Notably, specific enrichment of genes was observed for pathways involved in amino acid, lipid, and energy metabolism, including the citrate (TCA) cycle, glycolysis/gluconeogenesis, and fatty acid metabolism. Between stages R3 (14 DAP) and R2 (7 DAP), a total of 274 DEGs with a KEGG pathway annotation were found, and specific enrichment of starch and sucrose metabolic pathways was noted. This suggests that there are considerable differences between the physiological processes in the early and later stages of rice embryogenesis.

### Clustering of DEGs in the three developmental stages

**Figure 6:**
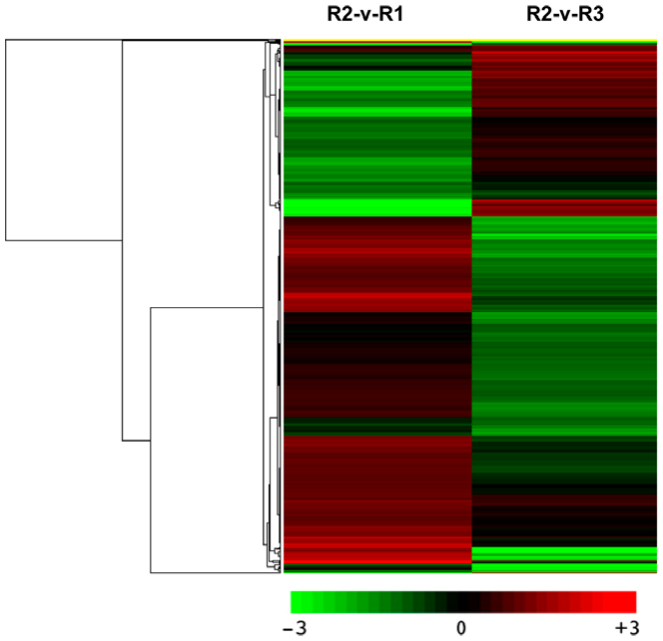
Hierarchical cluster analysis of gene expression based on log ratio RPKM data. Green represents lower expression, red represents high expression, column represent individual experiments, and rows represent transcriptional units. Two-dimensional hierarchical clustering classifies 1,011 differential expression profiles into four expression cluster groups according to the similarity of their expression profiles. Their identities and expression patterns are listed in Table S1.

RNA-Seq provides a platform for measuring differences in gene expression in a manner that is more sensitive than traditional microarray hybridization experiments [25]. We used our RNA-Seq data to analyze the expression of all previously annotated genes, as well as a set of novel transcripts that were uncovered in this study. RPKM values were determined for all genes in each of the stages tested, and the resulting data were transformed by first dividing each value for a gene at a particular stage by that gene's mean RPKM value across all stages and then taking the log (base 2) of the resulting values. Effectively, this transformed the data into the familiar log ratio values typically used for gene expression analyses. The expression profiles of the DEGs were determined by a cluster analysis based on the k-means method using Pearson's correlation distance. We did this so that we could determine the similarity in relative change for each transcript across the set of stages, and how those changes were similar or differed between transcripts. These data were then subjected to hierarchical clustering using the Pearson correlation as the distance metric (Figure 6). To identify clusters with functional enrichment, we determined a significant Pearson correlation through permutation analysis, as done previously [26]. We then cut the tree at this correlation, and the resulting clusters were refined by visual inspection and analyzed for GO term enrichment using Blast2GO (version 2.3.5) (http://www.blast2go.org/). We also clustered the RPKM data themselves, to provide a representation of absolute abundance for the transcripts, and noted that some clusters also showed functional enrichment, suggesting that many transcripts that contribute to a process are maintained at similar levels across stages.

**Figure 7:**
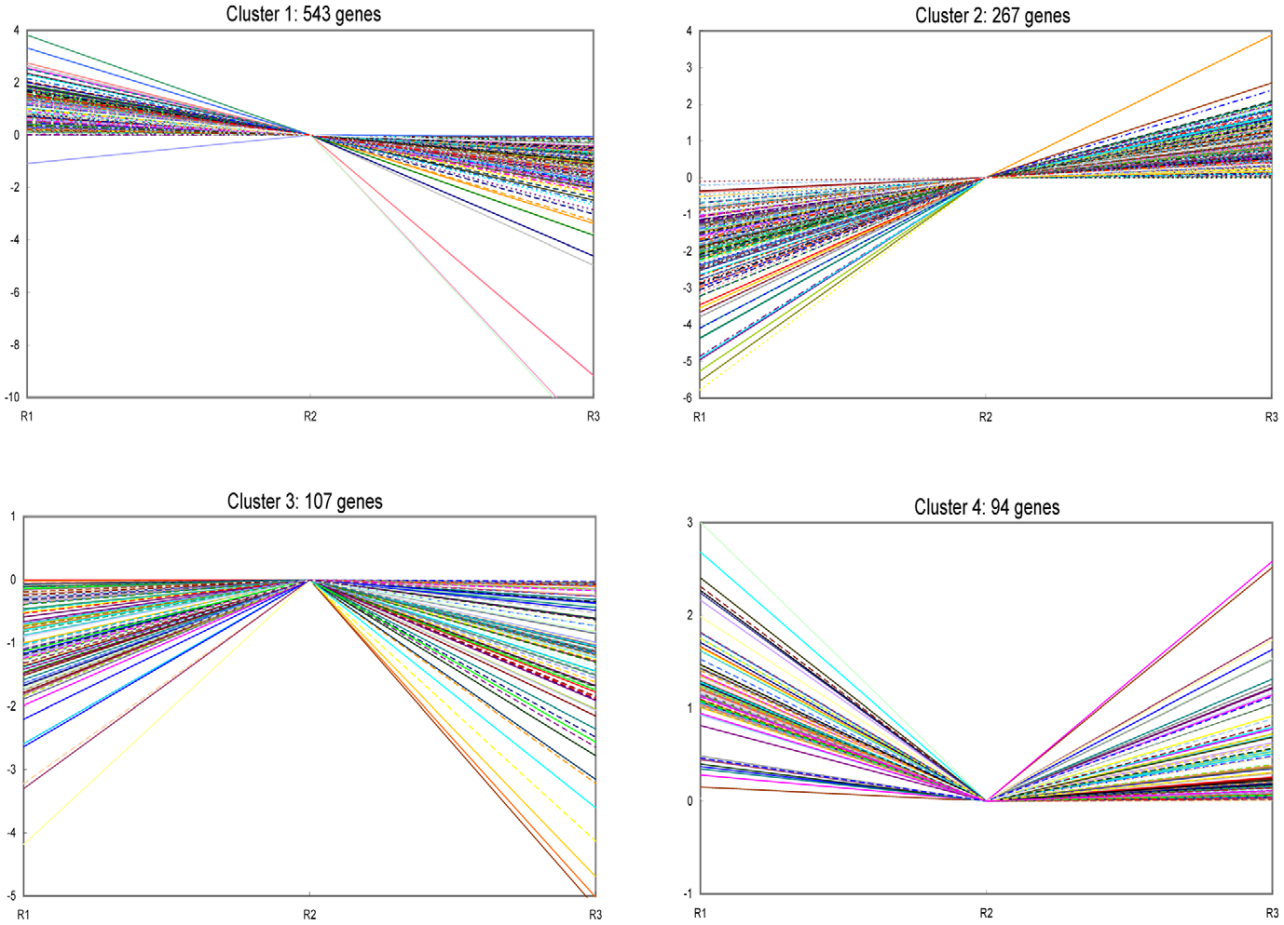
Expression patterns in the four expression clusters. Clusters were obtained by the k-means method on the gene expression profiles of the 1011 modulated genes. R1, 3–5 DAP; R2, 7 DAP; R3, 14 DAP. The most abundant group is Cluster 1, with 543 genes whose expression shows a negative slope during embryogenesis. The second abundant group is Cluster 2, which contained 267 genes whose expression shows a positive slope from 3 to 14 DAP. Cluster 3 is composed of 107 genes that begin to up-regulate at 3–5 DAP, peak at 7 DAP, and decrease thereafter. Cluster 4 consisted of 94 genes that are down-regulated from 3–5 to 7 DAP, then up-regulate to 14 DAP. Their identities and expression clusters are listed in Table S4.

Two-dimensional hierarchical clustering classified 1,011 differential expression profiles into four expression cluster groups (Clusters 1, 2, 3, and 4; Figures 5 and 6) according to the similarity of their expression profiles, representing the number of profiles indicated using figure of merit analysis. Visual inspection of these expression groups suggested diverse and complex patterns of regulation. Clusters 1 and 2 contained genes positively or negatively modulated throughout the whole time course, while genes expressed in the early and late stages of embryo development fell into Cluster 4; genes specifically induced at 7 DAP were grouped in Cluster 3 (Figure 7).

The most abundant group was Cluster 1, with 543 genes whose expression showed a negative slope during embryogenesis. These genes were expressed at their highest level at 3–5 DAP; they then decreased greatly at 7 and 14 DAP. The second most abundant group was Cluster 2, which contained 267 genes whose expression showed a positive slope from 3 to 14 DAP. Cluster 3 was composed of 107 genes that began to up-regulate at 3–5 DAP, peaked at 7 DAP, and decreased thereafter. Cluster 4 consisted of 94 genes that were down-regulated from 3–5 to 7 DAP, then up-regulated to 14 DAP (Figure 7 and Table S4).

The functional category distribution frequency was then calculated for each cluster to identify differences in the distribution of genes among the three developmental stages. Three major expression profiles corresponding to embryonic stages R1 (3–5 DAP), R2 (7 DAP), and R3 (14 DAP) were identified. In the early and middle stages, a large number of genes corresponding to DNA processing, signal transduction, and transcriptional regulation were found. Protein biosynthesis-related genes, including genes encoding ribosomal proteins and other components of the translation machinery, were highly expressed in the middle stage. Genes for protein modification were overrepresented later in the middle and late stages of embryogenesis.

We queried the Database of Rice Transcription Factors (http://drtf.cbi.pku.edu.cn/) and identified 857 putative TF genes. Quantitative analysis of their expression profiles indicated that TFs are expressed over a wide range of transcript abundances. Of the 857 putative TFs, 49 were differentially expressed (*P*<0.05) based on statistical analysis, and the expression profiles of these TFs are statistically enriched within four expression clusters (Table S5). Some TF families were identified, such as the *AP2* gene family, and the MADS-box gene family, which will be discussed in detail later. We identified five auxin-regulated genes differentially expressed, including auxin-repressed protein gene (Os03g22270, Os08g35190, and Os04g45370), *OsIAA18* gene (Os05g44810), and *OsSAUR19* gene (Os04g45370). The functions of these gene families above were clearly linked to the molecular and cellular changes that take place during rice embryo development.

## Discussion

RNA-Seq analysis created a comprehensive view of the participation of several multigene families in embryogenesis. Some members of these multigene families were identified, and their expression profiles were characterized in detail. The results indicated that these genes are likely to participate in the regulation of embryo development.

Auxin-regulated genes were identified among the transcripts that were differentially expressed during early embryo development. Auxins are important signaling molecules involved in many developmental processes in plants. Analyses of embryo-defective mutants affected in auxin transport or signaling indicate that auxins play various roles in coordinating the organization of the embryo by providing positional information [27]. Apical-to-basal auxin transport is initiated at the early globular stage and plays a key role in regulating many aspects of embryonic pattern formation in plants [3], [28]. A previous study on embryogenesis in *Arabidopsis* showing that auxin signaling events associated with gene activity are more prevalent in early developmental stages when the post-fertilization sporophytic program is initiated [9]. Among the auxin-regulated genes found in our transcriptomic analysis, we noticed up-regulation of the auxin-responsive *Aux/IAA* gene *OsIAA18* during rice embryo development, which indicated that this gene not only participates in early embryo developmental events, but also is involved in developmental processes at middle and late stages.

Among all the identified *AP2* transcription factors, five enriched in Cluster 1, showed a negative slope during embryo development. These genes were expressed at their highest level at 3–5 DAP, and then decreased substantially at 7 and 14 DAP. *AP2* transcription factors are involved in embryo initiation and development, and redundantly control embryo patterning via interactions with other genes [29]. We found that one transcription factor gene is enriched in Cluster 2 and showed a positive slope during embryo development, while one of the transcription factors is enriched in Cluster 3 and 4, respectively (Table S5). The complex regulation of these *AP2* genes indicated that the *AP2* transcription factor family plays an important role in the whole process of embryo development.

*AGL* genes belong to the MADS-box gene family, whose products contain a conserved MADS-box domain; some may encode a signaling substance, while others may act as transcription factors to regulate the expression of other genes during embryo development [2], [30]. We detected two *AGL* genes as DEGs: *ECAGL3* showed a down-regulated trend, with peak expression at R1 (3–5 DAP) followed by a rapid decline; *ECAGL1* showed steady up-regulation, indicating that each *AGL* gene has a different mission during embryo development.

*OSH* genes, which belong to the homeobox gene family, are expressed before the differentiation of embryonic organs, and play an important role in formation of the shoot apical meristem [31]. We identified four *OSH* genes as DEGs: *OsHKT1;3* and *OsHKT1;1* had similar expression patterns during early development, while *OsHKT1;4* showed an up-regulated trend and *OsHKT2;1* expression increased during early stages and then decreased, showing that homeotic genes collaborate with others with independent functions during embryo development.

A comparative expression profiling strategy between different developmental stages was used to identify a subset of genes that were differently expressed. Some potential regulators of embryogenesis have been identified. In the early and middle stages, a large number of genes corresponding to amino acid, lipid, and energy metabolism; nucleic acid replication/processing; signal transduction; and transcriptional regulation were found. Protein biosynthesis-related genes, including genes encoding ribosomal proteins and other components of the translation machinery, were highly expressed in embryos at the middle stage. Genes for protein modification and starch/sucrose metabolism were highly expressed later in the middle and late stages of embryogenesis. These results suggest that metabolism/cellular processes occurring in the embryo involve a transition from cell growth and differentiation to embryo maturation. In addition, we found that many gene families, especially transcription factor families, may play important roles at different developmental events, including embryo initiation as well as other developmental processes.

Despite the considerable depth of sequence coverage obtained, we did not detect expression of all of the previously annotated genes in the genome. It should be noted that all of the RNA that we analyzed in this study was put through two sequential rounds of poly(A) mRNA selection, so we did not expect to detect transcripts that are not polyadenylated. Long-lived transcripts with short poly(A) tails and short transcripts, which may have been size-selected against in our isolation protocol, would also be underrepresented in our data set.

In summary, RNA-Seq data from a diverse collection of developing rice embryos provided new tools for the analysis of large transcriptome data sets obtained by next generation sequencing. This study produced abundant data for the analysis of rice embryogenesis. Stage-specifically expressed genes were identified from three embryonic developmental stages. A comparative expression profiling strategy between different developmental stages provided a subset of genes that were differently expressed. The identification of genes involved in rice embryogenesis will extend our understanding of the complex molecular and cellular events in this development processes and provide a foundation for future studies on the metabolism, growth, and differentiation of embryos of rice and other cereal crops.

## Materials and Methods

### Plant materials

Rice (*O. sativa* L.ssp. *indica* cv.9311) plants were grown in a greenhouse at Wuhan University (30°33″N, 114°19″E), China. Some spikelets were tagged at the initiation of pollination, and harvested at 3–5, 7, and 14 DAP. Before 3 DAP, the zygote reiterates cell divisions to form a globular embryo with no apparent morphological differentiation. At 5 DAP, the first leaf primordium is visible on the opposite side of the coleoptile, and the shoot apical meristem becomes dome-shaped at 7 DAP [32]. Morphological maturity of the rice embryo was noted at approximately 14 DAP. Since the number of embryos at 3 DAP was extremely low, a mixture of embryos at 3–5 DAP was collected as one sample. The three-staged embryos were taken from spikelets with microdissection needles under a dissection microscope (Olympus, Tokyo, Japan) according to the manual microdissection method and frozen immediately in liquid nitrogen for total RNA extraction. Three embryo clusters were collected from 15 different plants at 3–5, 7, and 14 DAP during the growing season. The embryos were randomly selected from each cluster and pooled with embryos from other plants at the same greenhouse site, resulting in three independent pools for each developmental stage.

### Preparation of a cDNA library for RNA-Seq

Total RNA was extracted using TRIzol® reagent according to the manufacturer's protocol (Invitrogen, Burlington, ON, Canada). The yield and purity of RNA were assessed by determination of the absorbance (Abs) at 260 and 280 nm. RNA was only used when the Abs260 nm/Abs280 nm ratio was >1.8. RNA integrity was checked using a 1% agarose gel with the RNA 6000 Nano Assay Kit and Agilent 2100 Bioanalyzer. The extracted total RNA was stored at −70°C for later use.

The total RNA samples were pooled and 10 µg of total RNA from each pool was used to isolate poly(A) mRNA and to prepare a nondirectional Illumina RNA-Seq library with an mRNA-Seq 8 Sample Prep Kit (Illumina). We modified the gel extraction step by dissolving excised gel slices at room temperature to avoid underrepresentation of AT-rich sequences. Library quality control and quantification were performed with a Bioanalyzer Chip DNA 1000 series II (Agilent). Each library had an insert size of 200 bp; 42- to 50-bp sequences were generated via Illumina HiSeq™ 2000.

### Mapping reads to the reference genome and annotated genes

The rice genome and gene information were downloaded from the Rice Genome Annotation Project (http://rice.plantbiology.msu.edu). Sequencing-received raw image data were transformed by base culling into sequence data. Prior to mapping reads to the reference database, we filtered all sequences to remove adaptor sequences and low-quality sequences (the percentage of low-quality bases with a quality value ≤5 was >50% in a read). The remaining reads were aligned to the rice genome using SOAPaligner/soap2, allowing up to two base mismatches. Reads that failed to be mapped were progressively trimmed off, one base at a time from the 30-end and mapped to the genome again until a match was found (unless the read had been trimmed by <27 bases). For single-end reads, the insert between paired reads was set as 1 base-5 kilobases, allowing them to span introns of various sizes in the genome. The same strategy was used to align single-end reads to the non-redundant gene except that the insert was changed to 1 base-1 kilobase.

### Normalized expression levels of genes from RNA-Seq and GO analysis

ERANGE software (version 4.0) (http://woldlab.caltech.edu/gitweb/) was used to calculate the normalized gene locus expression level by assigning reads to their site of origin and counting them. The expression level of a gene from RNA-Seq was normalized by the RPKM value. The cutoff value for determining gene transcriptional activity was determined based on a 95% confidence interval for all RPKM values for each gene.

We obtained the GO terms for each rice gene using Blast2GO (version 2.3.5) (http://www.blast2go.org/) with the default parameters. Blast2GO was also used for a GO functional enrichment analysis of certain genes, by performing Fisher's exact test with a robust FDR correction to obtain an adjusted *p*-value between certain test gene groups and the whole genome annotation.

### Analysis of differential gene expression during rice embryo development

We applied the R package DEGseq to identify DEGs with the random sampling model based on the read count for each gene at different developmental stages [33]. We used “FDR ≤0.001 and the absolute value of log_2_Ratio ≥1” as the threshold for judging the significance of each gene expression difference. More stringent criteria with a smaller FDR and bigger fold-change value can be used to identify DEGs. GO functional enrichment analysis was carried out using Blast2GO (version 2.3.5) (http://www.blast2go.org/). KEGG pathway analyses of the DEGs were performed using Cytoscape software (version 2.6.2) (http://www.cytoscape.org/) with the ClueGO plugin (http://www.ici.upmc.fr/cluego/cluegoDownload.shtml) [34].

## Supporting Information

Table S1 **A set of 1011 differentially expressed genes during rice embryo development.** We used FDR<0.001 and the absolute value of log_2_Ratio≥1 as the threshold to judge the significance of gene expression difference. In order to calculate the log_2_Ratio and FDR, we used RPKM value of 0.001 instead of 0 for genes that do not express in one sample.
(DOC)

Table S2 **Differentially expressed genes between R1 and R2. FDR: false discovery rate.** We used FDR<0.001 and the absolute value of log_2_Ratio≥1 as the threshold to judge the significance of gene expression difference.
(DOC)

Table S3 **Differentially expressed genes between R3 and R2. FDR: false discovery rate.** We used FDR<0.001 and the absolute value of log_2_Ratio≥1 as the threshold to judge the significance of gene expression difference.
(DOC)

Table S4 **A list of GO categories for each cluster.** Functional annotation for each cluster utilized the corresponding GO Slim classification.
(XLS)

Table S5 **A list of putative transcription factors (TFs) differentially expressed during rice embryo development.** All the putative TFs are statistically enriched within specific expression clusters.
(XLS)
