# Supplementary material for: Transcriptomic Analysis of Rice (Oryza sativa) Developing Embryos Using the RNA-Seq Technique
Source: PLoS One. 2012 Feb 8;7(2):e30646. doi: 10.1371/journal.pone.0030646 (PMC3275597; doi:10.1371/journal.pone.0030646)
Supplement: Table S1 — A set of 1011 differentially expressed genes during rice embryo development. We used FDR<0.001 and the absolute value of log2Ratio≥1 as the threshold to judge the significance of gene expression difference. In order to calculate the log2Ratio and FDR, we used RPKM value of 0.001 instead of 0 for genes that do not express in one sample. (DOC) [file pone.0030646.s001.doc]

**Table S1** A set of 1011 differentially expressed genes during rice embryo development. We used FDR < 0.001 and the absolute value of log_2_Ratio≥1 as the threshold to judge the significance of gene expression difference. In order to calculate the log_2_Ratio and FDR, we used RPKM value of 0.001 instead of 0 for genes that do not express in one sample.

| ***Gene ID*** | ***R2-v-R1*** | ***R2-v-R3*** |
| --- | --- | --- |
| LOC_Os01g01870|13101.t00085 | 0.740486917089169 | -1.06198077713093 |
| LOC_Os01g02160|13101.t00113 | -1.00071302407162 | -0.483013081268794 |
| LOC_Os01g03360|13101.t00229 | -1.74424006236609 | 1.35343542144298 |
| LOC_Os01g03680|13101.t00258 | -1.17709358896284 | 0.404651089958769 |
| LOC_Os01g03690|13101.t00259 | 2.55695043156974 | -9.15928684094104 |
| LOC_Os01g04050|13101.t00293 | 1.8440185296032 | -1.40323400728979 |
| LOC_Os01g04280|13101.t00314 | 2.31051253626664 | -0.924066170591236 |
| LOC_Os01g04360|13101.t00323 | -2.23282152177111 | 1.42500500060356 |
| LOC_Os01g04370|13101.t00324 | -2.39915351913876 | 1.69319654994908 |
| LOC_Os01g04380|13101.t00325 | -2.37641237539997 | 1.35158364592835 |
| LOC_Os01g04409|13101.t00327 | 0.693976952491431 | -1.14913272622601 |
| LOC_Os01g04660|13101.t00351 | -3.66329352318813 | 1.62699592351163 |
| LOC_Os01g04670|13101.t00352 | 2.00522196561167 | -1.49890445403868 |
| LOC_Os01g04920|13101.t00375 | 1.54255105250913 | -1.69648730253945 |
| LOC_Os01g05630|13101.t00445 | 1.05484070992574 | -0.333656961972014 |
| LOC_Os01g05970|13101.t00477 | 1.00504342087413 | -0.367567133993772 |
| LOC_Os01g06560|13101.t00536 | 0.160783706390464 | -1.68518562646622 |
| LOC_Os01g06590|13101.t00539 | 1.12060242604549 | -0.0495970526750952 |
| LOC_Os01g06630|13101.t00543 | -4.09075966690415 | 1.95933104639488 |
| LOC_Os01g07970|13101.t00675 | 1.50010457116504 | -0.464634551953935 |
| LOC_Os01g08150|13101.t00692 | 0.336052256913622 | -1.02734424582797 |
| LOC_Os01g08380|13101.t00715 | 2.64595543762848 | -11.4902105541568 |
| LOC_Os01g08600|13101.t00738 | 1.26744381437476 | 0.162453424439897 |
| LOC_Os01g09030|13101.t00779 | 0.0487696812844251 | -1.40685822067474 |
| LOC_Os01g09220|13101.t00798 | 1.70848649212556 | -1.41344385341825 |
| LOC_Os01g09790|13101.t00855 | 0.285744631117697 | -1.21186848157398 |
| LOC_Os01g09800|13101.t00856 | 1.1463347288755 | -0.701673749254784 |
| LOC_Os01g10580|13101.t00929 | -1.38418972599168 | 1.01115697840401 |
| LOC_Os01g10860|13101.t00956 | 0.344785902907922 | -1.35076658739566 |
| LOC_Os01g11340|13101.t01000 | 0.365847518435756 | -1.81513179903807 |
| LOC_Os01g11460|13101.t01009 | 0.281118751271838 | 1.1430480252673 |
| LOC_Os01g12110|13101.t01072 | 1.76654557844208 | -3.37152514756245 |
| LOC_Os01g12580|13101.t01120 | -2.38686673303713 | 1.14727356293524 |
| LOC_Os01g13060|13101.t01165 | -1.02848907197966 | 0.180270489223504 |
| LOC_Os01g14850|13101.t01332 | 1.34092441201731 | -0.492337480743785 |
| LOC_Os01g15000|13101.t01348 | 1.74964674067072 | -0.674557621328168 |
| LOC_Os01g15290|13101.t01375 | 1.01690035272416 | -0.765804086674503 |
| LOC_Os01g15830|13101.t01429 | -1.37641237539997 | -0.276367914522114 |
| LOC_Os01g16170|13101.t01463 | 1.03949186769641 | -0.563038256014929 |
| LOC_Os01g16250|13101.t01471 | 1.72841759782046 | -1.27907889879786 |
| LOC_Os01g16920|13101.t01537 | -1.94460666274022 | 0.841290491969512 |
| LOC_Os01g17050|13101.t01550 | 1.04403146012846 | -0.7024021991773 |
| LOC_Os01g17170|13101.t01561 | 0.790541059511058 | -2.4825564599512 |
| LOC_Os01g18630|13101.t01648 | -1.17821964893484 | 1.17420631746906 |
| LOC_Os01g19820|13101.t01761 | -0.516590033448227 | -1.45398730775443 |
| LOC_Os01g21590|13101.t01883 | -0.46952177979145 | -1.44387599178714 |
| LOC_Os01g22954|13101.t02006 | 1.0998438658787 | -1.02651343956205 |
| LOC_Os01g24070|13101.t02108 | -1.56349792800046 | 1.21668301266235 |
| LOC_Os01g29150|13101.t02534 | 1.25810594979542 | -1.2489058608985 |
| LOC_Os01g29780|13101.t02587 | -1.97065268970913 | 0.269862786621519 |
| LOC_Os01g32380|13101.t02738 | -1.62276804008713 | 0.394502912206215 |
| LOC_Os01g34870|13101.t02967 | 1.01917276121271 | -0.655318113563043 |
| LOC_Os01g36950|13101.t03165 | 1.25820560751942 | 0.530240415868193 |
| LOC_Os01g37690|13101.t03231 | -1.02436429213627 | 0.423603472161886 |
| LOC_Os01g39020|13101.t03357 | -1.44054271281968 | 0.832833350943734 |
| LOC_Os01g40190|13101.t03466 | 1.89971202987428 | -0.349157334533995 |
| LOC_Os01g40650|13101.t03508 | 1.12896186058333 | -0.150880388512276 |
| LOC_Os01g41720|13101.t03613 | -1.47898210904051 | 1.14834232493682 |
| LOC_Os01g42520|13101.t03688 | -1.8988334100524 | 0.479512213312548 |
| LOC_Os01g43140|13101.t03749 | -0.376412375399967 | -1.01538133733718 |
| LOC_Os01g43480|13101.t03784 | 1.72602902932637 | -1.63455955339624 |
| LOC_Os01g43851|13101.t03819 | -3.54633737684228 | 0.867940787132933 |
| LOC_Os01g45624|13101.t03936 | -1.78383669056893 | -0.0726806657881333 |
| LOC_Os01g45659|13101.t03940 | -2.03720628908077 | 1.42711255739404 |
| LOC_Os01g46290|13101.t03997 | 0.688740304125288 | -1.23330900584748 |
| LOC_Os01g46580|13101.t04025 | -1.27624532545346 | 0.187470849548034 |
| LOC_Os01g47400|13101.t04109 | -1.44680170329136 | 0.130975192966726 |
| LOC_Os01g47760|13101.t04144 | 1.36103275842194 | -1.23786369010692 |
| LOC_Os01g49200|13101.t04281 | 1.19496715304826 | -0.687670889855814 |
| LOC_Os01g49320|13101.t04293 | 1.49911404929922 | -1.68186526817461 |
| LOC_Os01g49370|13101.t04298 | 1.24807848950783 | 0.158989569136327 |
| LOC_Os01g50420|13101.t04395 | 0.873529606661669 | -1.91557970470341 |
| LOC_Os01g50616|13101.t04415 | -0.82315873440615 | 1.2341959133255 |
| LOC_Os01g50700|13101.t04424 | -1.25270789380541 | 0.401464964223085 |
| LOC_Os01g51230|13101.t04476 | 0.13192884371828 | -1.38401609174565 |
| LOC_Os01g52110|13101.t04561 | 1.01659881754888 | 0.134827518462334 |
| LOC_Os01g52230|13101.t04572 | 0.413811851671985 | -1.34727760131578 |
| LOC_Os01g52500|13101.t04597 | 1.05702403470439 | -1.01564972075175 |
| LOC_Os01g52660|13101.t04612 | 0.119545119262449 | -2.01307117664998 |
| LOC_Os01g52830|13101.t04630 | -1.3729177551021 | 0.163602471050444 |
| LOC_Os01g53620|13101.t04702 | 1.25745972580214 | 0.301875317826287 |
| LOC_Os01g53710|13101.t04711 | 1.05494896057335 | -1.14689425403 |
| LOC_Os01g53990|13101.t04739 | 0.242781760140493 | -1.05777098412079 |
| LOC_Os01g54300|13101.t04768 | -1.35704705053303 | 0.189410082512253 |
| LOC_Os01g54340|13101.t04772 | 2.63853796606601 | -4.95648764828361 |
| LOC_Os01g54550|13101.t04793 | 1.43094254665764 | 0.252965717345339 |
| LOC_Os01g55000|13101.t04835 | -2.77695030498369 | 0.191411046828702 |
| LOC_Os01g55030|13101.t04838 | 1.45588890378787 | -0.34657879043223 |
| LOC_Os01g55160|13101.t04852 | -0.501943257483821 | 2.58267116282442 |
| LOC_Os01g55240|13101.t04859 | -0.57531644381404 | -3.50902867131239 |
| LOC_Os01g55430|13101.t04878 | 0.372936943222073 | 1.63158076330537 |
| LOC_Os01g55690|13101.t04902 | -1.08700320138768 | -0.43843519809153 |
| LOC_Os01g56220|13101.t04953 | 2.36055321876625 | -0.634559553396246 |
| LOC_Os01g56235|13101.t04955 | -1.16737833691853 | -0.480231407004948 |
| LOC_Os01g56610|13101.t04993 | -1.26668093474316 | 0.210510993046042 |
| LOC_Os01g56880|13101.t05019 | 0.636495108379426 | -1.02158267650549 |
| LOC_Os01g57690|13101.t05093 | -2.59053718075281 | -1.44814642916537 |
| LOC_Os01g58670|13101.t05185 | -2.77695030498369 | 1.56422031071825 |
| LOC_Os01g59020|13101.t05218 | -1.70983610912516 | 0.221050537268577 |
| LOC_Os01g59060|13101.t05222 | 1.05593970918105 | -0.0495970526750969 |
| LOC_Os01g59200|13101.t05236 | 7.36368720853366 | 11.6165019498148 |
| LOC_Os01g59350|13101.t05250 | 1.23585747131693 | -0.589330299403249 |
| LOC_Os01g59680|13101.t05281 | 2.752870641545 | -9.16993408514055 |
| LOC_Os01g60730|13101.t05379 | 1.50941660468073 | -0.180841585953343 |
| LOC_Os01g60740|13101.t05380 | 0.201960738889796 | -1.03736256481484 |
| LOC_Os01g60860|13101.t05391 | 1.2140045554133 | -1.39355145389246 |
| LOC_Os01g61080|13101.t05411 | 0.910750301326724 | -1.82300464280936 |
| LOC_Os01g62290|13101.t05528 | -1.27577414540189 | -0.263192525774384 |
| LOC_Os01g62420|13101.t05541 | 0.0239897775104833 | -1.00547449944279 |
| LOC_Os01g62900|13101.t05590 | 1.3704589617394 | -0.680729734829175 |
| LOC_Os01g63010|13101.t05599 | -1.68418509599905 | 0.553604566331105 |
| LOC_Os01g63210|13101.t05618 | -3.45820646656361 | 1.65520521805207 |
| LOC_Os01g63580|13101.t05652 | 0.474624985475042 | -1.35337780085219 |
| LOC_Os01g63690|13101.t05662 | 0.475436856650597 | -1.92406617059123 |
| LOC_Os01g64120|13101.t05703 | -1.42287168649262 | 0.268261377903222 |
| LOC_Os01g64256|13101.t05717 | 0.779505768048464 | -1.4032340072898 |
| LOC_Os01g64470|13101.t05739 | 1.56022556360261 | -0.879672051232777 |
| LOC_Os01g64640|13101.t05756 | 1.27008853563033 | -1.13595733747827 |
| LOC_Os01g64670|13101.t05759 | -2.98048369906883 | 1.58511848324316 |
| LOC_Os01g64900|13101.t05782 | 1.26218808892327 | -0.339103669870074 |
| LOC_Os01g65590|13101.t05846 | 0.548180221942015 | -11.6469901795421 |
| LOC_Os01g65650|13101.t05852 | 1.37746660170259 | -0.192555006517135 |
| LOC_Os01g65670|13101.t05854 | -1.52742016349965 | -0.403234007289792 |
| LOC_Os01g66830|13101.t05965 | 0.856248381390311 | -1.58911658263508 |
| LOC_Os01g66850|13101.t05967 | 1.33303215600234 | -1.01643018873989 |
| LOC_Os01g67850|13101.t06066 | 1.28514581610822 | -0.531466060432141 |
| LOC_Os01g68650|13101.t06141 | 1.29407894087823 | -1.17106056516076 |
| LOC_Os01g68660|13101.t06142 | 0.346937846908745 | -1.14883492561789 |
| LOC_Os01g68870|13101.t06163 | -1.0765105868426 | 0.40585471504966 |
| LOC_Os01g68950|13101.t06170 | 0.474187325056047 | -1.17240945827928 |
| LOC_Os01g69850|13101.t06209 | 1.80268094131594 | -0.252413935674781 |
| LOC_Os01g69870|13101.t06210 | 0.67486812954373 | -2.21006172486834 |
| LOC_Os01g70080|13101.t06232 | 0.597592416067093 | -3.27198947401154 |
| LOC_Os01g70560|13101.t06280 | 0.521200406620543 | -1.10618058104146 |
| LOC_Os01g70740|13101.t06298 | 0.0069162641515417 | -1.21560700411402 |
| LOC_Os01g70790|13101.t06303 | 1.06818126787687 | -0.301433251904018 |
| LOC_Os01g72530|13101.t06471 | 2.0159050473788 | -2.37152514756245 |
| LOC_Os01g73720|13101.t06587 | -4.18376729745757 | -0.856951974732697 |
| LOC_Os01g74040|13101.t06619 | 0.0875347243598259 | -1.08739908538748 |
| LOC_Os01g74370|13101.t06652 | 1.0051586659148 | -1.8569519747327 |
| LOC_Os02g01150|13102.t00015 | -1.58499899721138 | 1.04437909553392 |
| LOC_Os02g01970|13102.t00101 | 1.42550866060702 | 0.509830355938929 |
| LOC_Os02g02400|13102.t00143 | 1.57043232859786 | -1.49113454993199 |
| LOC_Os02g02410|13102.t00144 | 0.203331236371076 | -1.25162167631758 |
| LOC_Os02g03220|13102.t00224 | 1.01951630093118 | 0.47506493777825 |
| LOC_Os02g03580|13102.t00259 | 0.547352038772948 | -2.06667056603403 |
| LOC_Os02g04160|13102.t00317 | -2.63944678123376 | 1.88900240266076 |
| LOC_Os02g04369|13102.t00339 | -1.14021415453849 | 0.562846184468171 |
| LOC_Os02g04520|13102.t00354 | 1.03666360754559 | -0.800569504835178 |
| LOC_Os02g04780|13102.t00382 | -4.36191280570485 | 1.54986501774118 |
| LOC_Os02g05430|13102.t00447 | 3.00440940854097 | 1.53536544804606 |
| LOC_Os02g05680|13102.t00472 | 1.23887466217799 | 0.2087149429163 |
| LOC_Os02g05830|13102.t00487 | -1.22665974773327 | -0.174523664575924 |
| LOC_Os02g05890|13102.t00493 | 1.50190906801178 | -0.677628275288134 |
| LOC_Os02g06340|13102.t00539 | 0.0435478024479267 | -1.06091236590292 |
| LOC_Os02g06360|13102.t00541 | -0.309741335754682 | -1.17158757705371 |
| LOC_Os02g06410|13102.t00546 | -2.51915254751604 | 0.38877295043438 |
| LOC_Os02g06560|13102.t00561 | -1.34078846566924 | 0.530548431748288 |
| LOC_Os02g06725|13102.t05533 | 0.927813717753938 | 1.05228256134412 |
| LOC_Os02g06930|13102.t00599 | 1.47540683771488 | -0.355405482199178 |
| LOC_Os02g07180|13102.t00625 | 1.44432157645884 | -0.403234007289796 |
| LOC_Os02g07840|13102.t00692 | -1.99477803438833 | 1.09067467475284 |
| LOC_Os02g07870|13102.t00695 | 0.699497290669202 | -1.00150276447405 |
| LOC_Os02g08440|13102.t00754 | 1.30979010508435 | 0.0105239397624758 |
| LOC_Os02g09250|13102.t00786 | -4.37641237539996 | 2.05809081663928 |
| LOC_Os02g09810|13102.t00838 | 1.36055321876624 | -0.634559553396247 |
| LOC_Os02g09960|13102.t00853 | 0.611514792299464 | -1.19095290192063 |
| LOC_Os02g09980|13102.t00855 | 1.41944690781981 | -1.34505293620126 |
| LOC_Os02g09990|13102.t00856 | 2.04562720427761 | -1.24347678626913 |
| LOC_Os02g11070|13102.t00963 | 1.62904205469215 | -1.5090286713124 |
| LOC_Os02g15250|13102.t01332 | -2.0969904436303 | 0.617203740102523 |
| LOC_Os02g15280|13102.t01335 | 1.86797911901638 | -3.01643018873989 |
| LOC_Os02g15740|13102.t01381 | -1.85558021209853 | 0.626905852782199 |
| LOC_Os02g15810|13102.t01388 | 0.562680899910213 | -1.22065848193582 |
| LOC_Os02g15930|13102.t01400 | -1.46436007467566 | 1.82275512422895 |
| LOC_Os02g16500|13102.t01457 | 1.36541897393806 | -0.0692258594240249 |
| LOC_Os02g16540|13102.t01461 | 2.53047822020855 | -0.123597634118867 |
| LOC_Os02g16630|13102.t01471 | 1.11544072092971 | -0.421565830062049 |
| LOC_Os02g17280|13102.t01534 | 1.03984310203555 | -0.434621982887497 |
| LOC_Os02g19820|13102.t01784 | 0.571608096168416 | -1.06150381935926 |
| LOC_Os02g22020|13102.t01999 | 0.498056742516187 | -2.06340285220012 |
| LOC_Os02g25680|13102.t02277 | -1.20033514697606 | 1.19247773452305 |
| LOC_Os02g25780|13102.t02287 | 1.12771905070815 | -3.75003677081619 |
| LOC_Os02g26470|13102.t02353 | -1.56196802855604 | 0.535365448046066 |
| LOC_Os02g30600|13102.t02768 | -2.00868059089948 | 0.1794284609938 |
| LOC_Os02g31030|13102.t02811 | 0.967883532515855 | -1.59016543403779 |
| LOC_Os02g32580|13102.t02920 | 1.53047822020855 | -1.98033439023798 |
| LOC_Os02g32814|13102.t02942 | 0.226383418408512 | -1.80825771794061 |
| LOC_Os02g32860|13102.t02945 | -1.12022960262747 | 0.420255020611294 |
| LOC_Os02g32970|13102.t02956 | 0.773151672593668 | -1.67408791758289 |
| LOC_Os02g33070|13102.t02966 | -0.402407583932912 | 1.65400994454468 |
| LOC_Os02g33149|13102.t02974 | 1.34406509597767 | -0.0495970526750929 |
| LOC_Os02g33380|13102.t02997 | -1.20423140001728 | -0.0414920030380367 |
| LOC_Os02g35900|13102.t03245 | 0.608480732209822 | -1.49705602964632 |
| LOC_Os02g36340|13102.t03288 | 1.33193254082648 | -0.593917568898901 |
| LOC_Os02g36530|13102.t03307 | 2.16274643570807 | 0.628474852437546 |
| LOC_Os02g36700|13102.t03322 | 1.50494312810142 | -2.29752456611868 |
| LOC_Os02g37300|13102.t03382 | 0.330805875372196 | -2.6643068967903 |
| LOC_Os02g39000|13102.t03559 | -2.15882094032734 | 1.04770014867982 |
| LOC_Os02g39850|13102.t03646 | 1.1381607974298 | -2.8569519747327 |
| LOC_Os02g41510|13102.t03763 | 1.10238456810426 | -1.35215982269552 |
| LOC_Os02g41630|13102.t03775 | -1.08155252371077 | -0.238416365869875 |
| LOC_Os02g41840|13102.t03797 | 1.71253854750978 | -0.712562065397525 |
| LOC_Os02g41904|13102.t03804 | 1.06432314952743 | -1.81224517086731 |
| LOC_Os02g41954|13102.t03809 | 1.66540780029467 | -1.81513179903807 |
| LOC_Os02g42330|13102.t03845 | 0.922505741440898 | -1.12455911035631 |
| LOC_Os02g44230|13102.t04046 | 1.77840573365214 | -1.04959705267509 |
| LOC_Os02g44870|13102.t04110 | 1.4193518341738 | -1.3467825273461 |
| LOC_Os02g45450|13102.t04165 | 1.19344323293099 | -0.776578558268678 |
| LOC_Os02g45780|13102.t04198 | 0.650772453926266 | -1.08130591240243 |
| LOC_Os02g45930|13102.t04213 | 1.28066310340673 | -1.3958750089782 |
| LOC_Os02g45940|13102.t04214 | 0.815678281844819 | -1.00305446673806 |
| LOC_Os02g46210|13102.t04242 | 1.4049473381247 | -0.52603509661808 |
| LOC_Os02g46610|13102.t04281 | -0.90248118706755 | 1.95040294732491 |
| LOC_Os02g46910|13102.t04311 | 0.633571713172662 | -1.0231248413139 |
| LOC_Os02g47120|13102.t04332 | 1.07609982929754 | -0.371525147562455 |
| LOC_Os02g47130|13102.t04333 | 0.345040219394919 | -1.33596396233951 |
| LOC_Os02g47180|13102.t04338 | 0.492886332071159 | -1.18487591131051 |
| LOC_Os02g47310|13102.t04351 | 1.12385296074591 | -1.16824154917371 |
| LOC_Os02g47350|13102.t04355 | 0.114021443844375 | -1.06242109303268 |
| LOC_Os02g47670|13102.t04389 | 1.0249501869818 | 0.372229612266394 |
| LOC_Os02g47840|13102.t04405 | -0.278524554730532 | -1.01060292105923 |
| LOC_Os02g48320|13102.t04457 | 1.22262531053291 | -0.201600146120142 |
| LOC_Os02g48360|13102.t04461 | 1.16196500145782 | -0.335648111233857 |
| LOC_Os02g48570|13102.t04482 | -3.22440928195491 | 1.46853393956786 |
| LOC_Os02g49370|13102.t04563 | 3.8158804390708 | -1.0495970526751 |
| LOC_Os02g49520|13102.t04578 | -1.60702530354138 | 0.812899423574969 |
| LOC_Os02g49720|13102.t04598 | -0.87571879157641 | -1.23961347205687 |
| LOC_Os02g49840|13102.t04610 | 2.33783314226616 | -0.72766895778773 |
| LOC_Os02g50040|13102.t04631 | 0.822504829382059 | -1.54058340518723 |
| LOC_Os02g50990|13102.t04726 | 1.13816079742979 | 0.247384685082039 |
| LOC_Os02g52150|13102.t04844 | -0.727673964245495 | -2.04959705267509 |
| LOC_Os02g52210|13102.t04850 | 0.733107386723572 | -1.15651225659161 |
| LOC_Os02g52314|13102.t04860 | 0.287132149296872 | -1.41059471224104 |
| LOC_Os02g52560|13102.t04884 | -2.66591899259495 | 0.798399853879857 |
| LOC_Os02g53000|13102.t04927 | 0.736062353858447 | -1.18580143627341 |
| LOC_Os02g53620|13102.t04988 | 0.810356136205764 | -1.18475663595673 |
| LOC_Os02g54060|13102.t05033 | 1.2168177413048 | -1.00068745219414 |
| LOC_Os02g54140|13102.t05041 | -1.9785354272793 | 0.13326700447472 |
| LOC_Os02g54254|13102.t05052 | 1.22441653078021 | -0.119644550203356 |
| LOC_Os02g54730|13102.t05097 | 1.752870641545 | 0.700424694316558 |
| LOC_Os02g54980|13102.t05123 | 1.69397695249144 | -1.10849074172866 |
| LOC_Os02g55080|13102.t05133 | -1.36153909891844 | 0.881507866113839 |
| LOC_Os02g55910|13102.t05215 | 1.04567988141284 | -1.52562518184308 |
| LOC_Os02g56460|13102.t05271 | -0.997900752146234 | -4.69345324244981 |
| LOC_Os02g56540|13102.t05279 | -0.0160101326979593 | -1.06922585942402 |
| LOC_Os02g56680|13102.t05294 | -0.776183118025496 | -5.01922340363157 |
| LOC_Os02g56920|13102.t05318 | 0.489836235711207 | -1.13813372727675 |
| LOC_Os02g57110|13102.t05337 | -1.88847432907368 | -0.852560205615543 |
| LOC_Os02g57620|13102.t05387 | 1.2201380995964 | -0.418830862340813 |
| LOC_Os02g58260|13102.t05453 | 1.20032961851622 | -0.223626452450148 |
| LOC_Os03g01270|13103.t00029 | 0.167908140823846 | -1.23120385916504 |
| LOC_Os03g01770|13103.t00079 | 1.2991526741021 | 0.282978286411777 |
| LOC_Os03g02050|13103.t00106 | -2.07314812477764 | 0.973296859958256 |
| LOC_Os03g02290|13103.t00128 | 0.358114173314826 | -1.06922585942403 |
| LOC_Os03g02550|13103.t00153 | 1.84213797964209 | -4.60418590435273 |
| LOC_Os03g02780|13103.t00177 | 0.686028199375943 | -1.13650511789826 |
| LOC_Os03g03630|13103.t00251 | 0.286552637322468 | -1.01788819294775 |
| LOC_Os03g03724|13103.t00262 | 1.58155240480283 | -1.35215982269552 |
| LOC_Os03g03810|13103.t00270 | -2.09811121291286 | 1.85360535590331 |
| LOC_Os03g04070|13103.t00294 | 1.17900584970718 | -2.93712232341668 |
| LOC_Os03g04100|13103.t00296 | 1.16524634110033 | -0.531305591599218 |
| LOC_Os03g04110|13103.t00298 | 0.776761261753021 | -1.6159438752289 |
| LOC_Os03g04190|13103.t00304 | -1.72365978513684 | 0.51036144191617 |
| LOC_Os03g04220|13103.t00306 | -1.68675249601211 | -0.145812367934394 |
| LOC_Os03g04550|13103.t00338 | 0.0459176164452381 | -1.35215982269552 |
| LOC_Os03g04660|13103.t00349 | -1.82500243438983 | 1.02315928976022 |
| LOC_Os03g04680|13103.t00351 | -0.367642165772231 | 1.13200975381486 |
| LOC_Os03g04770|13103.t00359 | -1.42755122439176 | 0.864643776726999 |
| LOC_Os03g05334|13103.t00413 | 0.0556986372378232 | -1.77894946273142 |
| LOC_Os03g05540|13103.t00434 | 1.14053170189254 | 0.724843246909777 |
| LOC_Os03g05710|13103.t00451 | 0.747388576528586 | -1.01179501996271 |
| LOC_Os03g05750|13103.t00455 | 1.15426846428143 | -1.04098392237038 |
| LOC_Os03g06180|13103.t00497 | -1.4675110546703 | 0.806092461868249 |
| LOC_Os03g06580|13103.t00535 | 0.657713408504658 | -1.90374618621164 |
| LOC_Os03g06670|13103.t00542 | 0.658758402868866 | -1.03114014796753 |
| LOC_Os03g06705|13103.t05676 | -0.460476640188437 | -1.77751750723829 |
| LOC_Os03g07100|13103.t00588 | -1.07429276159278 | 0.928931417022081 |
| LOC_Os03g07180|13103.t00596 | -1.32936712371289 | 0.0950589956384005 |
| LOC_Os03g07380|13103.t00614 | 1.08687156873294 | -0.51256902901764 |
| LOC_Os03g07810|13103.t00655 | 0.489836235711205 | -1.17512793475895 |
| LOC_Os03g08220|13103.t00696 | -0.458206466563611 | -1.51471978339025 |
| LOC_Os03g08470|13103.t00721 | 0.0440795539709841 | -1.0065283307832 |
| LOC_Os03g09230|13103.t00793 | 1.65823376740693 | -0.897593959230042 |
| LOC_Os03g10240|13103.t00843 | 1.11110178520538 | -0.799618799666738 |
| LOC_Os03g10620|13103.t00881 | 1.66798174395849 | -0.786562646841303 |
| LOC_Os03g10880|13103.t00908 | 1.17378470716052 | -0.930952556176477 |
| LOC_Os03g11490|13103.t00967 | -0.0951262650099469 | 1.41710256642625 |
| LOC_Os03g11500|13103.t00968 | 1.01477838192451 | -0.456772434180967 |
| LOC_Os03g11734|13103.t00990 | 0.982041595512511 | -2.01307117664998 |
| LOC_Os03g11910|13103.t01006 | -1.1360141660092 | 0.79133646135729 |
| LOC_Os03g12510|13103.t01064 | -0.689719987714982 | -2.77998999291782 |
| LOC_Os03g12730|13103.t01086 | -1.31435140726772 | 0.632432866006228 |
| LOC_Os03g12900|13103.t01103 | -0.108152945306187 | -1.0577249335585 |
| LOC_Os03g12990|13103.t01112 | -1.07786720795055 | 0.518255117623641 |
| LOC_Os03g13300|13103.t01142 | 0.491484088592688 | -1.7865626468413 |
| LOC_Os03g14180|13103.t01228 | -5.26393764614156 | 1.41337492366745 |
| LOC_Os03g14300|13103.t01241 | 0.460088892317155 | -1.26110115786881 |
| LOC_Os03g15270|13103.t01332 | 1.96046606095337 | -1.46463455195393 |
| LOC_Os03g15530|13103.t01358 | -1.21446316953468 | 0.0110576314593587 |
| LOC_Os03g15540|13103.t01359 | 1.00964605690711 | 0.557060519145382 |
| LOC_Os03g15560|13103.t01361 | 1.09173646033844 | 0.306546757550187 |
| LOC_Os03g15750|13103.t01380 | 1.03777422733938 | 0.199762416261624 |
| LOC_Os03g16260|13103.t01430 | -1.48332757931648 | -5.17888006962005 |
| LOC_Os03g16480|13103.t01449 | 1.20718328955729 | 0.030130139795644 |
| LOC_Os03g16670|13103.t01465 | 1.74302285558866 | -0.982482856816556 |
| LOC_Os03g16860|13103.t01483 | -0.218460015623729 | -1.13557027578495 |
| LOC_Os03g16920|13103.t01490 | -2.16996149793253 | 3.88658784972747 |
| LOC_Os03g16940|13103.t01492 | -1.80218275146721 | 0.500261647090152 |
| LOC_Os03g17200|13103.t01518 | -0.278027635329226 | -1.04271066708985 |
| LOC_Os03g17470|13103.t01542 | 0.4860841008501 | 1.52071867208166 |
| LOC_Os03g17690|13103.t01561 | 1.11925233487973 | -0.827204631338647 |
| LOC_Os03g17790|13103.t01571 | -1.06053332518541 | 1.14493316213672 |
| LOC_Os03g17900|13103.t01582 | 1.15672440233117 | 0.038630130336635 |
| LOC_Os03g17940|13103.t01586 | 1.23181990464404 | 0.17781344342798 |
| LOC_Os03g17960|13103.t01588 | 0.212161378873572 | -1.01741664692595 |
| LOC_Os03g18264|13103.t01618 | 0.398566544702975 | 1.04526213366635 |
| LOC_Os03g18770|13103.t01667 | 1.68895080898316 | -0.642687434279644 |
| LOC_Os03g18960|13103.t01685 | -0.87162022336279 | 1.20038120033325 |
| LOC_Os03g19720|13103.t01761 | 1.15247810025424 | -0.822826190828067 |
| LOC_Os03g20090|13103.t01796 | 0.994425319968347 | -1.27822142772305 |
| LOC_Os03g20120|13103.t01799 | -1.46952177979145 | 0.972770760353359 |
| LOC_Os03g20460|13103.t01830 | 1.28947012070476 | 0.249963229183815 |
| LOC_Os03g20680|13103.t01851 | -1.20782932074187 | 0.133656682456403 |
| LOC_Os03g20700|13103.t01853 | 1.20411787900593 | -1.3322360164484 |
| LOC_Os03g21260|13103.t01907 | -1.37641237539996 | 2.08790647107484 |
| LOC_Os03g21640|13103.t01943 | 1.62754563816872 | -1.27198947401154 |
| LOC_Os03g21650|13103.t01944 | -1.89098554822972 | 1.32891457057864 |
| LOC_Os03g21790|13103.t01959 | -1.03597881166053 | 0.4680101802443 |
| LOC_Os03g22270|13103.t02007 | 1.12143946155115 | -0.554541524307073 |
| LOC_Os03g22790|13103.t02057 | 1.01417571538763 | -0.657423661747986 |
| LOC_Os03g26490|13103.t02360 | -1.33171256792915 | 0.738898842131194 |
| LOC_Os03g26870|13103.t02394 | -0.678975145420394 | 1.51573664129713 |
| LOC_Os03g27280|13103.t02430 | -0.698340470287324 | 1.69886418032894 |
| LOC_Os03g27800|13103.t02480 | -2.08690575820498 | 1.45854985099523 |
| LOC_Os03g28160|13103.t02515 | -1.17866369173333 | 0.144905971487888 |
| LOC_Os03g28330|13103.t02530 | 0.573682889671198 | -1.31391971188511 |
| LOC_Os03g28990|13103.t02546 | -1.74037569008454 | 0.413374923667454 |
| LOC_Os03g29190|13103.t02565 | 1.34144439581854 | -0.975596471231317 |
| LOC_Os03g29250|13103.t02576 | 0.654052905327084 | -1.94771743865588 |
| LOC_Os03g30530|13103.t02686 | -1.39151926779017 | 1.13031203733984 |
| LOC_Os03g31679|13103.t02790 | 1.89304829959326 | -0.535023879845335 |
| LOC_Os03g31750|13103.t02797 | -1.56169162371931 | 1.20203569790716 |
| LOC_Os03g36560|13103.t03143 | -0.0544842805125905 | -1.06708447940393 |
| LOC_Os03g37490|13103.t03219 | 0.498056742516176 | -1.63455955339625 |
| LOC_Os03g39000|13103.t03360 | 1.0074434686306 | -0.23863087706511 |
| LOC_Os03g39020|13103.t03362 | 0.550018406604197 | -1.17274528344796 |
| LOC_Os03g40540|13103.t03495 | 1.02561860680545 | -0.95773619448087 |
| LOC_Os03g42420|13103.t03652 | -0.424240900490589 | -1.25123091384475 |
| LOC_Os03g42430|13103.t03653 | -1.04040909530088 | 0.22049211069265 |
| LOC_Os03g42464|13103.t03656 | 0.151966596954826 | 2.51674976987872 |
| LOC_Os03g42520|13103.t03661 | -1.08095649187379 | 0.927266448526975 |
| LOC_Os03g43720|13103.t03767 | 1.16790814082385 | 0.0759338294087679 |
| LOC_Os03g44380|13103.t03830 | 1.10261668057213 | -0.443511260388182 |
| LOC_Os03g44540|13103.t03846 | 1.83242194064176 | -0.535023879845334 |
| LOC_Os03g44810|13103.t03869 | 1.0486092124515 | -0.482556459951198 |
| LOC_Os03g45250|13103.t03908 | -0.00104502155114049 | -1.77751750723829 |
| LOC_Os03g45280|13103.t03911 | 1.0670167245351 | -1.30660767088112 |
| LOC_Os03g45760|13103.t03955 | 0.678231840652243 | -1.13705989392543 |
| LOC_Os03g46100|13103.t03985 | -1.4058160265576 | 0.743640293228298 |
| LOC_Os03g46440|13103.t04018 | 1.72723471455816 | -1.0694966101128 |
| LOC_Os03g46640|13103.t04037 | 0.469077675544411 | -1.02406196056796 |
| LOC_Os03g46920|13103.t04063 | 0.206279951696367 | -1.04959705267509 |
| LOC_Os03g47530|13103.t04120 | 0.563046082664984 | -2.15195877030477 |
| LOC_Os03g47980|13103.t04171 | 1.04281292084232 | -0.153933712489825 |
| LOC_Os03g48390|13103.t04210 | 1.27809105857427 | -0.16062836506384 |
| LOC_Os03g49440|13103.t04304 | 1.88258446376094 | -3.82218655657202 |
| LOC_Os03g51010|13103.t04442 | -1.03937738812239 | 0.675168087885594 |
| LOC_Os03g51080|13103.t04448 | 1.71105046585038 | -0.912093528925162 |
| LOC_Os03g51350|13103.t04474 | -2.22548471625062 | 0.800429045104648 |
| LOC_Os03g51390|13103.t04477 | 1.50010457116503 | -2.04959705267509 |
| LOC_Os03g52320|13103.t04564 | 0.0111040611149745 | -1.41696811832362 |
| LOC_Os03g52380|13103.t04570 | -3.0544842805126 | 1.60575477593746 |
| LOC_Os03g52650|13103.t04593 | 0.127818853208333 | -1.00895506817775 |
| LOC_Os03g52680|13103.t04596 | 1.27590539312295 | -1.81513179903807 |
| LOC_Os03g52860|13103.t04612 | -5.53561097024922 | 1.24625753060237 |
| LOC_Os03g53020|13103.t04629 | 1.57112020470589 | -1.4239925674566 |
| LOC_Os03g53610|13103.t04685 | -4.96137487612112 | 0.833589282342155 |
| LOC_Os03g53620|13103.t04686 | -2.78589034933691 | 0.843487743408399 |
| LOC_Os03g53740|13103.t04698 | 0.336940661544285 | -1.45840259824243 |
| LOC_Os03g53900|13103.t04711 | -1.71953763382775 | 0.493490713676504 |
| LOC_Os03g53930|13103.t04714 | 1.56022556360261 | -1.0495970526751 |
| LOC_Os03g54050|13103.t04726 | -2.50699648521011 | 1.22150678313458 |
| LOC_Os03g55120|13103.t04782 | 0.838600515570888 | -1.14074494073329 |
| LOC_Os03g55240|13103.t04793 | 2.21560488285514 | -1.63455955339624 |
| LOC_Os03g55430|13103.t04812 | 1.43490056022632 | 0.0573181512414211 |
| LOC_Os03g55540|13103.t04821 | 1.86062682190088 | -1.13448595026161 |
| LOC_Os03g55590|13103.t04825 | 1.29216624378599 | -1.96318230085761 |
| LOC_Os03g55776|13103.t04844 | 2.15891935759659 | -2.5731590087321 |
| LOC_Os03g56070|13103.t04872 | 0.390300562160286 | -1.44693255022048 |
| LOC_Os03g56590|13103.t04923 | 0.717709801964586 | -1.52533048364149 |
| LOC_Os03g57310|13103.t05005 | 1.19167630675679 | -1.89089930665603 |
| LOC_Os03g57690|13103.t05040 | -1.13627837167625 | 0.664468139381031 |
| LOC_Os03g57960|13103.t05064 | -1.29549549243568 | 0.618050762727726 |
| LOC_Os03g58040|13103.t05072 | 1.01285696481697 | -1.36057673665398 |
| LOC_Os03g58580|13103.t05120 | -0.0901081902433221 | 1.49974153831535 |
| LOC_Os03g58650|13103.t05127 | -0.141947121762935 | -1.74474247114667 |
| LOC_Os03g58830|13103.t05143 | 1.14800858338614 | -0.654459110833956 |
| LOC_Os03g59300|13103.t05185 | 0.480964265166977 | -1.41300178353862 |
| LOC_Os03g59460|13103.t05201 | -1.28435182241119 | 0.196563534594306 |
| LOC_Os03g60380|13103.t05286 | 0.225215578985464 | -1.33218425593028 |
| LOC_Os03g60570|13103.t05305 | 1.75950373387745 | -0.755865849618381 |
| LOC_Os03g61240|13103.t05367 | 2.26744381437476 | 0.172795368661355 |
| LOC_Os03g62590|13103.t05499 | -1.04973075054197 | 0.0866073309232178 |
| LOC_Os03g62650|13103.t05505 | 0.029173648534308 | 1.05423875830945 |
| LOC_Os03g63280|13103.t05558 | -1.45502221009633 | 0.0352918449114208 |
| LOC_Os04g01230|13104.t00024 | 1.66172275348681 | 0.258525242687241 |
| LOC_Os04g01690|13104.t00066 | -2.37641237539996 | 0.582671162824421 |
| LOC_Os04g01740|13104.t00070 | -2.20334766642709 | -0.26259077600929 |
| LOC_Os04g02530|13104.t00145 | 2.00680284027154 | -1.79566485394855 |
| LOC_Os04g02670|13104.t00158 | 1.1339608089005 | 0.2245779107639 |
| LOC_Os04g02754|13104.t00165 | -3.69834047028732 | 1.84570556865822 |
| LOC_Os04g05050|13104.t00383 | -1.24950026291775 | -0.820115206552327 |
| LOC_Os04g08350|13104.t00699 | 1.12464321397574 | -1.75666116112744 |
| LOC_Os04g09390|13104.t00770 | -1.22122867229914 | 0.120629967896942 |
| LOC_Os04g09800|13104.t00806 | 1.38045277620395 | -0.219522054117406 |
| LOC_Os04g10460|13104.t00870 | 0.0658099532051107 | -1.20392519906638 |
| LOC_Os04g13260|13104.t01135 | 1.05048527908895 | 0.777071344439355 |
| LOC_Os04g14150|13104.t01221 | -1.04607204069641 | 0.872400435323633 |
| LOC_Os04g15800|13104.t01327 | 0.164525501429193 | -1.0261380798511 |
| LOC_Os04g16450|13104.t01387 | 0.629653625299388 | -1.96054586485796 |
| LOC_Os04g16830|13104.t01425 | 1.65077245392627 | 0.918694087597569 |
| LOC_Os04g17660|13104.t01503 | -0.349227546073733 | -1.04959705267509 |
| LOC_Os04g18950|13104.t01624 | -1.13465462919658 | 0.0263518005582042 |
| LOC_Os04g19960|13104.t01722 | 0.2471714193485 | -1.52166549699031 |
| LOC_Os04g20774|13104.t01797 | -2.63944678123376 | 0.687368541491111 |
| LOC_Os04g21110|13104.t01827 | 1.36055321876624 | 0.780477945882594 |
| LOC_Os04g22290|13104.t01944 | 0.592336690615595 | -1.33088316306511 |
| LOC_Os04g23550|13104.t02064 | 0.772530918296028 | -1.67171255181371 |
| LOC_Os04g28250|13104.t02503 | -0.826215292839481 | 1.16096993326457 |
| LOC_Os04g28420|13104.t02519 | -0.796291137748306 | -1.05686505313913 |
| LOC_Os04g28820|13104.t02557 | -1.30389811025609 | 1.09326576841085 |
| LOC_Os04g29274|13104.t02601 | 3.32402734274113 | -0.0495970526750917 |
| LOC_Os04g30420|13104.t02709 | 1.29950502360706 | -0.679840432697116 |
| LOC_Os04g31524|13104.t02814 | 0.227147344198525 | -1.05826191380222 |
| LOC_Os04g31924|13104.t02852 | 0.978530776715359 | -1.07656410027536 |
| LOC_Os04g32080|13104.t02866 | -1.85881404935255 | 0.28895130413272 |
| LOC_Os04g32540|13104.t02910 | 0.24190872226763 | -1.07513214478223 |
| LOC_Os04g33740|13104.t03030 | 1.37435901829127 | -1.33499927153734 |
| LOC_Os04g33820|13104.t03038 | 1.11620023399852 | -1.26290160118359 |
| LOC_Os04g33990|13104.t03054 | -2.97009209322223 | 1.49422675325258 |
| LOC_Os04g35270|13104.t03179 | -1.5139158991499 | -1.13979486164667 |
| LOC_Os04g35490|13104.t03201 | -1.97522100198795 | 0.46161710806475 |
| LOC_Os04g38790|13104.t03431 | -1.46047664018844 | -0.607592505795979 |
| LOC_Os04g38940|13104.t03447 | -1.67339411315709 | -0.617880812249618 |
| LOC_Os04g39150|13104.t03468 | -1.37012611255253 | 0.391969929358566 |
| LOC_Os04g39864|13104.t03531 | -1.74636198515027 | -0.0495970526750941 |
| LOC_Os04g40510|13104.t03595 | -1.41853785107264 | -0.0970757628419767 |
| LOC_Os04g40540|13104.t03598 | -1.5799457694851 | -0.315671912828937 |
| LOC_Os04g40630|13104.t03606 | 1.09663345233953 | -0.922591775939766 |
| LOC_Os04g40730|13104.t03615 | 0.386088310873373 | -1.57154975587045 |
| LOC_Os04g40950|13104.t03638 | 0.380769541716316 | -1.02423462055366 |
| LOC_Os04g41900|13104.t03730 | 0.191198790810066 | -1.00760296401047 |
| LOC_Os04g41970|13104.t03736 | -2.79864537608301 | 0.838510067254016 |
| LOC_Os04g42860|13104.t03817 | 0.353600458124475 | -1.58011176937387 |
| LOC_Os04g43170|13104.t03848 | -1.150951205146 | 0.0515121701114077 |
| LOC_Os04g43230|13104.t03854 | 0.206640535312563 | -1.11998638056649 |
| LOC_Os04g43360|13104.t03865 | -1.80225040628041 | 1.058027440853 |
| LOC_Os04g44210|13104.t03947 | -2.36764216577223 | 0.669666539757657 |
| LOC_Os04g44510|13104.t03977 | 1.45104375271416 | -1.60902446128911 |
| LOC_Os04g45370|13104.t04059 | 1.27893945321259 | 0.798399853879859 |
| LOC_Os04g45610|13104.t04083 | 1.12712252597735 | -0.362754937934722 |
| LOC_Os04g45970|13104.t04116 | 1.656672127304 | -0.999323846193867 |
| LOC_Os04g46280|13104.t04144 | 1.08403558356883 | -0.360384589957259 |
| LOC_Os04g46390|13104.t04156 | -0.850843642789367 | 1.13265232763808 |
| LOC_Os04g46980|13104.t04217 | 1.55037777764626 | -1.46463455195394 |
| LOC_Os04g47220|13104.t04239 | 0.781735458265995 | -1.35403772083362 |
| LOC_Os04g47250|13104.t04241 | -0.352825555082348 | -1.19983968825571 |
| LOC_Os04g47330|13104.t04249 | 0.242675909225821 | -1.74620490922516 |
| LOC_Os04g47580|13104.t04272 | 0.414391370393793 | -1.11123611846748 |
| LOC_Os04g47590|13104.t04273 | -0.764977663317616 | 1.49929619333854 |
| LOC_Os04g48270|13104.t04337 | 2.02080384679164 | -1.04959705267509 |
| LOC_Os04g48350|13104.t04344 | 1.48156861972761 | -1.1698912863928 |
| LOC_Os04g49370|13104.t04446 | 1.04958817363509 | -1.04279722056975 |
| LOC_Os04g49450|13104.t04453 | 1.38161483429407 | -0.648773412615948 |
| LOC_Os04g49520|13104.t04459 | 1.43094254665764 | 0.239909564519891 |
| LOC_Os04g49550|13104.t04462 | 1.57170088293507 | -0.0495970526750932 |
| LOC_Os04g49980|13104.t04507 | -1.66642529021056 | 0.32235791895164 |
| LOC_Os04g50700|13104.t04544 | 0.26744381437476 | -1.18286358353855 |
| LOC_Os04g50960|13104.t04571 | -0.158820940327337 | -1.11830980275911 |
| LOC_Os04g51090|13104.t04584 | 1.54497778990367 | -0.0939911720335467 |
| LOC_Os04g51130|13104.t04588 | 2.3717804741895 | -2.04959705267509 |
| LOC_Os04g51460|13104.t04624 | 0.206588608987908 | -1.1637680725944 |
| LOC_Os04g52110|13104.t04689 | -1.28727907316871 | 0.161955789746159 |
| LOC_Os04g52260|13104.t04703 | 1.6909428924018 | -0.0495970526750926 |
| LOC_Os04g52370|13104.t04714 | 1.12837977663721 | -0.214656298945592 |
| LOC_Os04g52750|13104.t04752 | -2.42899933749418 | 0.246408597427045 |
| LOC_Os04g53950|13104.t04874 | 0.0227808458140998 | -1.40219684330487 |
| LOC_Os04g54210|13104.t04902 | 1.67421369767 | -0.593917568898905 |
| LOC_Os04g54620|13104.t04942 | -1.17232077080646 | 0.243761890015492 |
| LOC_Os04g55120|13104.t04990 | 0.552173291307872 | -1.65104767618482 |
| LOC_Os04g55410|13104.t05019 | 1.19647729302061 | -0.605990401199475 |
| LOC_Os04g55730|13104.t05050 | -1.35704705053303 | 0.865070893525148 |
| LOC_Os04g56230|13104.t05100 | 0.531817148416851 | -1.67945609137058 |
| LOC_Os04g56240|13104.t05101 | 0.360553218766244 | -1.19993132575952 |
| LOC_Os04g56400|13104.t05117 | 0.700039685037909 | -1.05944483863144 |
| LOC_Os04g56590|13104.t05135 | 1.07104660157126 | 0.0608269370185609 |
| LOC_Os04g56610|13104.t05137 | 1.70801640576074 | 0.187442144625757 |
| LOC_Os04g58890|13104.t05363 | 1.77739296067907 | -0.445525729006234 |
| LOC_Os04g59150|13104.t05387 | -1.63944678123376 | -0.988196508010949 |
| LOC_Os04g59260|13104.t05398 | -1.81055869762651 | -0.0591198267936001 |
| LOC_Os04g59520|13104.t05422 | -2.19198780426253 | 1.70042469431657 |
| LOC_Os05g01970|13105.t00097 | 0.726029029326362 | -1.00379336306197 |
| LOC_Os05g02060|13105.t00106 | 0.0301469699545748 | -1.07180833176526 |
| LOC_Os05g02070|13105.t00107 | -0.208685009154067 | -1.01055077514121 |
| LOC_Os05g02300|13105.t00130 | 0.910411918531483 | -1.29060515217888 |
| LOC_Os05g03620|13105.t00259 | 0.344686813307222 | -1.01397314294437 |
| LOC_Os05g04700|13105.t00366 | -1.12159847637114 | 0.285387195037715 |
| LOC_Os05g04820|13105.t00378 | -1.3175186863464 | -0.827204631338647 |
| LOC_Os05g04870|13105.t00383 | -3.78562789722158 | 1.33178202101332 |
| LOC_Os05g05480|13105.t00442 | 1.14904911357253 | 0.365440446603751 |
| LOC_Os05g05930|13105.t00487 | -1.92895339842874 | 1.16904323380025 |
| LOC_Os05g06440|13105.t00539 | 0.370268373599456 | -1.14357320088411 |
| LOC_Os05g07220|13105.t00614 | 1.26744381437476 | -0.464634551953933 |
| LOC_Os05g07560|13105.t00643 | -1.799911453427 | 1.98169907781445 |
| LOC_Os05g07890|13105.t00675 | -0.589521555902075 | -1.67578221612277 |
| LOC_Os05g08044|13105.t00691 | -1.31972295727313 | 0.326370881925158 |
| LOC_Os05g08420|13105.t00730 | 1.05552472426782 | -0.559672341765256 |
| LOC_Os05g09500|13105.t00836 | 0.800317803947469 | -1.17792114965063 |
| LOC_Os05g11090|13105.t00955 | 0.815455178923028 | 1.2217049691423 |
| LOC_Os05g11510|13105.t00995 | -0.928953398428742 | 1.81289942357497 |
| LOC_Os05g13580|13105.t01153 | 1.60848073220983 | -0.412167132059802 |
| LOC_Os05g15510|13105.t01344 | 1.12608796512922 | -1.00895506817775 |
| LOC_Os05g15770|13105.t01370 | -1.27687670184905 | 0.666609981324317 |
| LOC_Os05g20450|13105.t01829 | -0.0995721700411389 | 1.45941659481277 |
| LOC_Os05g20460|13105.t01830 | -1.10695170040674 | 1.17647102680475 |
| LOC_Os05g25640|13105.t02246 | -1.13965166377528 | 0.0045103526643542 |
| LOC_Os05g25770|13105.t02258 | 1.35671115247185 | -2.14475428571544 |
| LOC_Os05g27090|13105.t02390 | 0.654466937484007 | -1.13532692670098 |
| LOC_Os05g28210|13105.t02498 | -1.38196674580898 | 0.267108789958665 |
| LOC_Os05g29010|13105.t02575 | 1.00549782431563 | -0.192555006517136 |
| LOC_Os05g29050|13105.t02579 | 0.347614163058744 | 1.31297302670962 |
| LOC_Os05g30490|13105.t02672 | -2.84456121113837 | 0.97624295226017 |
| LOC_Os05g30860|13105.t02711 | 1.12483941893196 | -0.182863583538557 |
| LOC_Os05g31020|13105.t02726 | -2.04323952334498 | 0.841402170163536 |
| LOC_Os05g31280|13105.t02748 | 0.448016060016582 | -4.21952205411741 |
| LOC_Os05g31620|13105.t02783 | 1.07754411317118 | -0.581448216936691 |
| LOC_Os05g31670|13105.t02788 | -2.35952059600119 | 1.85172208980962 |
| LOC_Os05g33140|13105.t02936 | -5.7824047350758 | 2.00384220628637 |
| LOC_Os05g33380|13105.t02959 | -0.0559349543813267 | -1.29906595555576 |
| LOC_Os05g33400|13105.t02961 | 0.468193941954214 | -1.55548798240504 |
| LOC_Os05g33460|13105.t02967 | 0.495712802047884 | -1.11998638056649 |
| LOC_Os05g33690|13105.t02991 | 0.270682188441309 | -1.13301306086273 |
| LOC_Os05g33820|13105.t03004 | 2.04505139303831 | -0.156512256591612 |
| LOC_Os05g34170|13105.t03039 | -0.376412375399968 | -1.17687771670892 |
| LOC_Os05g34270|13105.t03049 | 1.3432752273344 | 0.295051118712353 |
| LOC_Os05g35140|13105.t03136 | 2.35579968879561 | -2.71256206539752 |
| LOC_Os05g35200|13105.t03142 | -1.37398563800542 | 0.7162234041633 |
| LOC_Os05g35360|13105.t03158 | -0.764977663317617 | 1.5748938122327 |
| LOC_Os05g35500|13105.t03173 | 0.787450873255147 | -2.0770777890972 |
| LOC_Os05g35980|13105.t03218 | 1.16790814082384 | 1.26160463561921 |
| LOC_Os05g36270|13105.t03248 | 2.23239686727557 | -1.10849074172866 |
| LOC_Os05g36280|13105.t03249 | 0.599804290844379 | -1.23098116949247 |
| LOC_Os05g38230|13105.t03395 | -1.49058339531927 | -0.121947896899772 |
| LOC_Os05g39230|13105.t03497 | 0.773904341417202 | -1.29921094274656 |
| LOC_Os05g39250|13105.t03499 | -1.94541258136513 | 0.502257136234128 |
| LOC_Os05g39310|13105.t03505 | -0.901133503605808 | -2.06314358451304 |
| LOC_Os05g39320|13105.t03506 | -1.06855946572432 | -2.48817006641472 |
| LOC_Os05g39690|13105.t03543 | -3.22885518698611 | 1.85250833991363 |
| LOC_Os05g39720|13105.t03546 | 1.50845191387856 | -1.70167374925478 |
| LOC_Os05g40010|13105.t03574 | -1.37641237539996 | 0.450476550459554 |
| LOC_Os05g41390|13105.t03711 | 0.578458249110377 | -1.00161702377326 |
| LOC_Os05g41670|13105.t03739 | 1.31474952915312 | -1.82720463133864 |
| LOC_Os05g41760|13105.t03748 | 1.19082253277185 | -0.678759357306782 |
| LOC_Os05g41780|13105.t03750 | 1.05135388338309 | 0.0918162161259239 |
| LOC_Os05g43170|13105.t03844 | 1.5213760131013 | -1.60759250579598 |
| LOC_Os05g43460|13105.t03872 | -1.18983613340749 | 0.577800714366463 |
| LOC_Os05g44060|13105.t03932 | 1.82932270198287 | -2.27198947401155 |
| LOC_Os05g44290|13105.t03955 | 1.79966485302394 | -0.0495970526750982 |
| LOC_Os05g44340|13105.t03960 | -1.55256933055379 | 0.611796111290918 |
| LOC_Os05g44810|13105.t04008 | -0.469521779791443 | 2.03786578857525 |
| LOC_Os05g45030|13105.t04031 | 0.151966596954828 | -1.30525192814329 |
| LOC_Os05g45460|13105.t04074 | 1.08524048315401 | -1.45111341230032 |
| LOC_Os05g45730|13105.t04101 | -0.21120978420006 | 1.00249145847335 |
| LOC_Os05g45740|13105.t04102 | 1.09235710781667 | -0.487718165066976 |
| LOC_Os05g46480|13105.t04177 | -1.5422842607982 | 0.493865669519178 |
| LOC_Os05g46760|13105.t04205 | 1.67912230156369 | -1.89089930665603 |
| LOC_Os05g47540|13105.t04236 | 1.22791545018812 | -1.55861070016295 |
| LOC_Os05g47870|13105.t04267 | -1.56729699547792 | 1.28460937199071 |
| LOC_Os05g47960|13105.t04276 | 1.47894791956847 | -1.49016964406107 |
| LOC_Os05g48930|13105.t04373 | 1.46398280842165 | -0.674087917582888 |
| LOC_Os05g49060|13105.t04386 | 0.709075523665321 | -2.15393371248983 |
| LOC_Os05g49160|13105.t04396 | 1.54997439583435 | -0.806325901662732 |
| LOC_Os05g49300|13105.t04410 | -2.42574008776353 | 1.04426987078913 |
| LOC_Os05g49730|13105.t04454 | -1.98737008465406 | -0.354451634203515 |
| LOC_Os05g49860|13105.t04467 | 1.44234730992989 | -0.683469153877196 |
| LOC_Os05g50710|13105.t04553 | 0.448016060016579 | 1.76631988288594 |
| LOC_Os05g50750|13105.t04557 | 0.725389905122893 | -1.60759250579598 |
| LOC_Os05g50910|13105.t04574 | 1.12837977663721 | 0.0077361223908585 |
| LOC_Os06g02000|13106.t00092 | -0.539911107682842 | 1.89793552743077 |
| LOC_Os06g02490|13106.t00138 | -1.21922904284923 | 0.664351888766142 |
| LOC_Os06g03520|13106.t00241 | -1.33459219970534 | -4.13705989392543 |
| LOC_Os06g03670|13106.t00256 | 1.06099293690733 | 0.130975192966723 |
| LOC_Os06g03710|13106.t00261 | 0.767014823865276 | -1.07468803363793 |
| LOC_Os06g03930|13106.t00281 | 0.56125056900337 | -1.59212428707585 |
| LOC_Os06g04230|13106.t00308 | 1.01980950183471 | -0.617072126667407 |
| LOC_Os06g04240|13106.t00309 | 2.19344323293098 | -1.04959705267509 |
| LOC_Os06g04460|13106.t00331 | -1.0544842805126 | 0.0487050209367131 |
| LOC_Os06g04800|13106.t00364 | 1.02158237740299 | -0.0868299588740706 |
| LOC_Os06g04940|13106.t00378 | -3.13110556211552 | 0.808383942452479 |
| LOC_Os06g04990|13106.t00383 | -1.38597398854449 | -0.357300507842614 |
| LOC_Os06g05000|13106.t00384 | -1.12037221616906 | -2.64749860910375 |
| LOC_Os06g05010|13106.t00385 | -1.36814475941636 | -2.15269054563919 |
| LOC_Os06g05020|13106.t00386 | -1.27114033229991 | -2.35817559390344 |
| LOC_Os06g05480|13106.t00433 | -1.63944678123376 | -0.0296974952373891 |
| LOC_Os06g05740|13106.t00459 | 0.67444399458819 | -1.10404483669747 |
| LOC_Os06g05860|13106.t00472 | 0.65869506078924 | -1.02857927140601 |
| LOC_Os06g06350|13106.t00521 | 1.45030787152457 | -0.504162916140574 |
| LOC_Os06g06460|13106.t00532 | 0.670335883791517 | -1.1478991262869 |
| LOC_Os06g06980|13106.t00582 | -1.02572528753118 | -0.0690932283008909 |
| LOC_Os06g07600|13106.t00643 | 2.01297868726614 | -2.79683098229513 |
| LOC_Os06g08250|13106.t00706 | -0.584998997211381 | 1.70939484782111 |
| LOC_Os06g08440|13106.t00723 | 1.09038934887434 | -0.584634328064565 |
| LOC_Os06g09600|13106.t00839 | -0.271714996733268 | -1.37930249815417 |
| LOC_Os06g10650|13106.t00939 | 0.523689054545923 | -1.2451478617929 |
| LOC_Os06g11090|13106.t00983 | -1.7115965669896 | -0.822186556572019 |
| LOC_Os06g12150|13106.t01090 | 1.54885674952341 | -0.357719348037421 |
| LOC_Os06g14406|13106.t01314 | 1.06518595015001 | 1.21018437536361 |
| LOC_Os06g14420|13106.t01316 | 1.02658733013472 | -1.3143002788695 |
| LOC_Os06g14460|13106.t01320 | 0.352691100993268 | -1.08130591240244 |
| LOC_Os06g14620|13106.t01336 | -0.0812843398563163 | -1.02328577555925 |
| LOC_Os06g18010|13106.t01672 | -0.307603217481314 | -1.49254054852382 |
| LOC_Os06g18670|13106.t01689 | -1.56472286307847 | 0.0196203891736591 |
| LOC_Os06g19444|13106.t01764 | 1.54341727591605 | -0.129767401359076 |
| LOC_Os06g21400|13106.t01956 | 1.19344323293098 | 0.380390788069724 |
| LOC_Os06g21910|13106.t02007 | -2.15499808927504 | 0.657709922001086 |
| LOC_Os06g22060|13106.t02022 | 0.929027596698831 | -1.8085889531713 |
| LOC_Os06g22919|13106.t02107 | 2.30626306326529 | -2.50902867131239 |
| LOC_Os06g22960|13106.t02110 | 1.04593938755192 | -1.18695253937796 |
| LOC_Os06g23274|13106.t02139 | -1.1726224793956 | 0.534183395508909 |
| LOC_Os06g23350|13106.t02146 | -2.26320176495197 | 1.04718800985979 |
| LOC_Os06g23800|13106.t02188 | 1.81058613940129 | 0.0499386208758236 |
| LOC_Os06g24430|13106.t02249 | -1.43557444786811 | 0.251572482045472 |
| LOC_Os06g25500|13106.t02349 | 0.463364024350017 | 1.1633966706591 |
| LOC_Os06g26180|13106.t02417 | -4.8618392025702 | 0.35250139089626 |
| LOC_Os06g27760|13106.t02471 | -1.88230330512992 | 0.430012447899836 |
| LOC_Os06g30130|13106.t02699 | -0.827073784409532 | 1.06858437367322 |
| LOC_Os06g30179|13106.t02703 | -1.73560041386271 | 0.895249777277111 |
| LOC_Os06g30370|13106.t02720 | -0.378098752027848 | -1.19115119688911 |
| LOC_Os06g31890|13106.t02869 | -2.49324784966671 | 0.611175051721716 |
| LOC_Os06g32240|13106.t02904 | -1.92301811807024 | 0.363527480523401 |
| LOC_Os06g32350|13106.t02915 | -1.08363062617212 | -0.627364051992041 |
| LOC_Os06g32355|13106.t04874 | -3.22440928195492 | -0.634559553396252 |
| LOC_Os06g32370|13106.t02917 | -1.63944678123375 | -0.731856754751163 |
| LOC_Os06g33330|13106.t03011 | -1.55592342567148 | 0.687368541491112 |
| LOC_Os06g34780|13106.t03154 | 1.6824813136536 | -0.27198947401154 |
| LOC_Os06g36490|13106.t03324 | 1.14714958065705 | 0.113901679607787 |
| LOC_Os06g36560|13106.t03331 | 1.52691160518836 | -1.79728905343436 |
| LOC_Os06g38294|13106.t03499 | 0.373331823487547 | -1.18402337289602 |
| LOC_Os06g39140|13106.t03585 | 0.793512626042354 | -1.53502387984533 |
| LOC_Os06g39370|13106.t03607 | 1.67731960853783 | -1.08436247083577 |
| LOC_Os06g41030|13106.t03775 | 1.61049520082788 | -3.24528515018249 |
| LOC_Os06g42020|13106.t03872 | 0.836809460044897 | -1.48023140700495 |
| LOC_Os06g42660|13106.t03938 | 0.872397790283382 | -1.40977261691942 |
| LOC_Os06g42754|13106.t03947 | -1.20828961659164 | 0.313873304455455 |
| LOC_Os06g43044|13106.t03974 | 0.722239022891338 | -1.0495970526751 |
| LOC_Os06g43600|13106.t04026 | 1.22728668790278 | -2.16743354296895 |
| LOC_Os06g43810|13106.t04047 | 1.29268110587378 | -0.889132380481848 |
| LOC_Os06g43960|13106.t04062 | -0.196503285385029 | 1.13031203733985 |
| LOC_Os06g44080|13106.t04075 | 0.93411095621515 | -1.10754440188438 |
| LOC_Os06g44190|13106.t04087 | -1.5139158991499 | 0.900362264825313 |
| LOC_Os06g45090|13106.t04178 | 1.12130364063061 | -1.27829772647977 |
| LOC_Os06g45140|13106.t04183 | 1.35679129997004 | -2.46463455195393 |
| LOC_Os06g45640|13106.t04234 | 1.34037533682861 | -1.2195220541174 |
| LOC_Os06g45990|13106.t04269 | 0.255200218620014 | -1.00951961329976 |
| LOC_Os06g46140|13106.t04285 | 2.11544072092971 | -2.0495970526751 |
| LOC_Os06g46284|13106.t04297 | 1.07975029537146 | -2.05559579329654 |
| LOC_Os06g46340|13106.t04301 | 0.506341314327039 | -1.41123485736443 |
| LOC_Os06g47250|13106.t04391 | 0.482781478177251 | -1.27786604034821 |
| LOC_Os06g48060|13106.t04473 | -1.15810691158676 | 0.411132537296622 |
| LOC_Os06g48500|13106.t04517 | -0.767603132724439 | 1.02764594625737 |
| LOC_Os06g48600|13106.t04528 | 0.281383005332636 | -1.45069536099606 |
| LOC_Os06g49100|13106.t04577 | -0.482232831541005 | -1.12759956467637 |
| LOC_Os06g49190|13106.t04587 | -1.43120484824034 | 0.381037301654771 |
| LOC_Os06g49250|13106.t04593 | -0.682515503125645 | 1.16062065471525 |
| LOC_Os06g49760|13106.t04645 | 0.532672433621467 | -1.13788358509305 |
| LOC_Os06g49880|13106.t04657 | 0.945515719487398 | 2.58267116282442 |
| LOC_Os06g50230|13106.t04692 | 1.9455157194874 | -12.7091320366341 |
| LOC_Os07g02790|13107.t00176 | 2.14714958065705 | -0.564170225504853 |
| LOC_Os07g04450|13107.t00339 | 1.4620912452283 | 0.61336796004734 |
| LOC_Os07g04500|13107.t00344 | 1.1536239148176 | -0.235152705831165 |
| LOC_Os07g05020|13107.t00393 | -1.84589765870118 | -0.149132726226002 |
| LOC_Os07g05040|13107.t00396 | 0.335462237803414 | -1.73765304636035 |
| LOC_Os07g05370|13107.t00430 | -1.04080934361553 | 0.783051889546426 |
| LOC_Os07g05390|13107.t00432 | 1.25132414901148 | -0.4121671320598 |
| LOC_Os07g05820|13107.t00475 | 0.650059835961226 | -1.10404483669747 |
| LOC_Os07g06840|13107.t00572 | -0.248862325679455 | -1.62248672109568 |
| LOC_Os07g07530|13107.t00637 | 2.6824813136536 | 0.535365448046064 |
| LOC_Os07g07930|13107.t00677 | 1.03808183822347 | -0.965204865384722 |
| LOC_Os07g08500|13107.t00729 | 1.02797787967937 | -0.851657675063183 |
| LOC_Os07g09060|13107.t00784 | 1.06372568438555 | -0.884194609923477 |
| LOC_Os07g09630|13107.t00839 | -0.721908941425732 | -3.21952205411741 |
| LOC_Os07g09970|13107.t00873 | -0.51016376428879 | 1.03786578857525 |
| LOC_Os07g10460|13107.t00923 | 1.81700097896472 | -1.60213807570387 |
| LOC_Os07g10570|13107.t00935 | -1.36423766145386 | 0.261531641359746 |
| LOC_Os07g10580|13107.t00936 | -1.4816555354273 | -0.101259172497588 |
| LOC_Os07g10810|13107.t00959 | 2.50701924738456 | -0.611475940283207 |
| LOC_Os07g12340|13107.t01108 | 1.18808602276147 | -1.15495005282133 |
| LOC_Os07g14150|13107.t01282 | 1.11248740354036 | -0.79841236471904 |
| LOC_Os07g14270|13107.t01294 | 0.778096006489209 | -1.03205162310348 |
| LOC_Os07g14310|13107.t01298 | 1.23502233668239 | 0.149711755548314 |
| LOC_Os07g14350|13107.t01302 | 1.01792421501545 | -0.182863583538551 |
| LOC_Os07g16970|13107.t01560 | -0.88455927907029 | 1.6228282892964 |
| LOC_Os07g18750|13107.t01739 | 0.918010033087493 | -1.46117899331772 |
| LOC_Os07g20340|13107.t01843 | -2.38388749596211 | 0.789883452117046 |
| LOC_Os07g20420|13107.t01850 | 2.31474952915312 | 0.82487206524105 |
| LOC_Os07g22224|13107.t01927 | -1.30756296764407 | 0.587308079807066 |
| LOC_Os07g22400|13107.t01945 | 1.15496908511636 | -0.634559553396241 |
| LOC_Os07g22580|13107.t01961 | 0.588581174772117 | -1.19502749219666 |
| LOC_Os07g23570|13107.t02050 | 3.04985237930213 | -0.786562646841301 |
| LOC_Os07g24000|13107.t02091 | -2.09881343848054 | 0.525980336981784 |
| LOC_Os07g25002|13107.t04585 | 1.0245872904786 | 0.158207832438308 |
| LOC_Os07g25810|13107.t02269 | 1.12385296074591 | 0.483835147405982 |
| LOC_Os07g28480|13107.t02532 | -2.01384229601525 | 0.89640805768418 |
| LOC_Os07g28850|13107.t02570 | -9.2099472851331 | 1.98602685705563 |
| LOC_Os07g32600|13107.t02931 | 0.166424649648517 | -1.33564811123386 |
| LOC_Os07g32680|13107.t02939 | 1.08301924323733 | -1.33499927153735 |
| LOC_Os07g33240|13107.t02993 | 0.354672967474181 | -1.11421991756733 |
| LOC_Os07g36170|13107.t03275 | 1.00338221373724 | -0.435830523306554 |
| LOC_Os07g36500|13107.t03306 | 0.945515719487402 | -1.17670897057843 |
| LOC_Os07g36590|13107.t03314 | -0.327104735175597 | -1.04007427855659 |
| LOC_Os07g36610|13107.t03316 | -1.0544842805126 | -0.0609123659029274 |
| LOC_Os07g37030|13107.t03356 | 1.40494733812469 | -1.63455955339625 |
| LOC_Os07g37400|13107.t03393 | 1.58075142668978 | -0.356258390909146 |
| LOC_Os07g37454|13107.t03398 | 0.100793944965307 | -1.8085889531713 |
| LOC_Os07g37570|13107.t03409 | 1.06099293690733 | 0.558085524546149 |
| LOC_Os07g38130|13107.t03465 | -1.49030334192794 | 0.75162308321041 |
| LOC_Os07g38290|13107.t03480 | -1.53331843037242 | 1.13614846624962 |
| LOC_Os07g39920|13107.t03642 | -1.31082403377239 | 1.15349481270242 |
| LOC_Os07g39980|13107.t03648 | 1.06626787894801 | -0.354451634203513 |
| LOC_Os07g41200|13107.t03762 | 0.849300404228094 | -1.20678038522582 |
| LOC_Os07g41240|13107.t03766 | 0.29129055632913 | -1.54709671214591 |
| LOC_Os07g41280|13107.t03770 | 0.713642245140063 | -1.03081984564447 |
| LOC_Os07g42324|13107.t03871 | 0.633855960053864 | -1.10849074172866 |
| LOC_Os07g42910|13107.t03929 | -1.21202555749908 | 1.51868670689944 |
| LOC_Os07g43170|13107.t03953 | 1.11132461245748 | -0.312631458508886 |
| LOC_Os07g43540|13107.t03990 | 1.61294038040053 | -0.436620175784339 |
| LOC_Os07g44140|13107.t04046 | 1.37208824913272 | -2.11734165931093 |
| LOC_Os07g44180|13107.t04049 | 0.513158744008532 | -1.19816106215337 |
| LOC_Os07g44430|13107.t04073 | -2.17295218174312 | 0.606715977637346 |
| LOC_Os07g45300|13107.t04158 | 1.22115016210083 | 0.402915152022415 |
| LOC_Os07g46360|13107.t04263 | -2.88164768365038 | 0.565648014982111 |
| LOC_Os07g46480|13107.t04275 | 0.700075373668348 | -1.18887184127722 |
| LOC_Os07g46560|13107.t04284 | 0.371441534908979 | -1.28663624997594 |
| LOC_Os07g46990|13107.t04329 | -0.489531740522939 | 1.07866362216434 |
| LOC_Os07g47620|13107.t04392 | -0.0713580990769928 | -3.15393371248983 |
| LOC_Os07g47790|13107.t04409 | -0.825002434389836 | -1.70594418663301 |
| LOC_Os07g47840|13107.t04414 | -2.6733941131571 | 1.75295688311869 |
| LOC_Os07g47960|13107.t04426 | 1.68744756654696 | -0.5856499529153 |
| LOC_Os07g48280|13107.t04456 | 1.19062821732393 | -1.45870971799683 |
| LOC_Os07g48460|13107.t04474 | -1.19080575172538 | -0.6316062360069 |
| LOC_Os07g48510|13107.t04479 | 0.761465113455336 | -1.17562670573743 |
| LOC_Os07g48710|13107.t04499 | 1.47444418529423 | -1.09141722836972 |
| LOC_Os07g49360|13107.t04564 | -1.97202212032062 | 0.90733422543302 |
| LOC_Os08g01370|13108.t00038 | -4.10223741196098 | 2.38646559314079 |
| LOC_Os08g01610|13108.t00063 | 1.25884779068637 | -0.0073688172857946 |
| LOC_Os08g01760|13108.t00078 | 0.897917339115004 | -1.00068745219415 |
| LOC_Os08g03410|13108.t00243 | -1.41271527426983 | 0.24161420618404 |
| LOC_Os08g03420|13108.t00244 | -0.314636177813273 | -1.69345324244981 |
| LOC_Os08g03690|13108.t00272 | -1.11564814319099 | 0.553399370876327 |
| LOC_Os08g04460|13108.t00347 | 1.35777613409567 | -0.290605152178879 |
| LOC_Os08g05530|13108.t00453 | 0.0028488945533485 | -1.21465629894559 |
| LOC_Os08g06100|13108.t00505 | -2.2508814933161 | 0.0997805713631337 |
| LOC_Os08g10500|13108.t00942 | 1.22052276698726 | -0.543706122945137 |
| LOC_Os08g10510|13108.t00943 | 0.701645318189831 | -1.19973740210295 |
| LOC_Os08g13920|13108.t01280 | 0.222562215693916 | -1.10254593262405 |
| LOC_Os08g14400|13108.t01324 | 1.55849259637815 | -1.55209739320428 |
| LOC_Os08g14580|13108.t01342 | 1.16790814082385 | 0.223421441731323 |
| LOC_Os08g15296|13108.t04310 | 1.98504408367403 | 1.73467425626947 |
| LOC_Os08g17160|13108.t01596 | 0.162327108560548 | -1.14357320088411 |
| LOC_Os08g17680|13108.t01644 | 0.364468268769626 | -1.08130591240243 |
| LOC_Os08g18920|13108.t01715 | 1.15063014939201 | -0.634559553396252 |
| LOC_Os08g18974|13108.t01721 | -0.362057082422898 | 1.26074306793706 |
| LOC_Os08g19140|13108.t01740 | 1.44432157645884 | 0.886272609905195 |
| LOC_Os08g19210|13108.t01747 | 1.13396080890051 | 0.0477001486798231 |
| LOC_Os08g19670|13108.t01791 | 1.13007967471661 | -0.760090435480107 |
| LOC_Os08g21541|13108.t01952 | 1.30165952971268 | -0.445525729006231 |
| LOC_Os08g23170|13108.t02092 | -2.14267227636021 | 0.918887400614191 |
| LOC_Os08g23870|13108.t02162 | -1.75402848127098 | 0.130920546462264 |
| LOC_Os08g27824|13108.t02546 | 1.1082452195255 | -0.0071617863546945 |
| LOC_Os08g29110|13108.t02673 | 1.16790814082385 | -0.84101043086367 |
| LOC_Os08g29669|13108.t02727 | -0.191987804262538 | -1.28663624997594 |
| LOC_Os08g30020|13108.t02760 | 1.15608270542706 | 0.602479643904602 |
| LOC_Os08g30210|13108.t02778 | -3.30241179395619 | -1.29752456611868 |
| LOC_Os08g31850|13108.t02936 | 2.33017956972272 | -1.21952205411741 |
| LOC_Os08g31980|13108.t02949 | 1.38487289796166 | -1.34295599536568 |
| LOC_Os08g32600|13108.t03012 | 1.04197855898869 | -0.559791784994277 |
| LOC_Os08g32910|13108.t03041 | 0.568331119207093 | -1.2690421899612 |
| LOC_Os08g33100|13108.t03061 | 0.43650207199954 | -1.45169549624644 |
| LOC_Os08g34280|13108.t03178 | -1.29959677834913 | 0.359793883462612 |
| LOC_Os08g35190|13108.t03269 | 1.25217705772145 | -1.39063397051016 |
| LOC_Os08g36320|13108.t03382 | -1.19057685545483 | 0.294186230638979 |
| LOC_Os08g36910|13108.t03439 | 2.04843403243296 | -4.19934417217977 |
| LOC_Os08g36920|13108.t03440 | 1.27446424205253 | -0.574259043128431 |
| LOC_Os08g37432|13108.t03491 | 1.1381607974298 | -0.856951974732696 |
| LOC_Os08g37580|13108.t03504 | -1.10102686644963 | -0.354451634203511 |
| LOC_Os08g37660|13108.t03513 | 0.734011614293691 | -1.68799881235771 |
| LOC_Os08g37700|13108.t03517 | 1.03090721071804 | 0.475493992068668 |
| LOC_Os08g38270|13108.t03573 | -1.32241748575923 | -0.347277601315778 |
| LOC_Os08g38460|13108.t03591 | 1.53047822020855 | 0.0435123517163887 |
| LOC_Os08g38710|13108.t03616 | 2.09751881293245 | -0.634559553396248 |
| LOC_Os08g39050|13108.t03648 | 1.61794106145889 | 0.53536544804606 |
| LOC_Os08g39694|13108.t03712 | 2.22930868548799 | -2.5731590087321 |
| LOC_Os08g40620|13108.t03804 | 1.26501707698022 | -0.774489814540723 |
| LOC_Os08g40720|13108.t03814 | -1.63944678123376 | 0.643425193903511 |
| LOC_Os08g41280|13108.t03869 | 0.272877700425392 | -1.02263000507482 |
| LOC_Os08g41440|13108.t03885 | 0.628126784215204 | -1.14999894963293 |
| LOC_Os08g42470|13108.t03987 | 0.308085798872103 | -1.04959705267509 |
| LOC_Os08g42720|13108.t04012 | -2.30241179395619 | 1.09479285666008 |
| LOC_Os08g42910|13108.t04031 | -1.96468761016195 | 1.02142241064493 |
| LOC_Os08g43730|13108.t04110 | 0.34092441201731 | -1.01112290486046 |
| LOC_Os08g44270|13108.t04161 | 1.23787069899141 | -1.45768179131217 |
| LOC_Os08g44750|13108.t04208 | -1.0655394690216 | -0.275665132154937 |
| LOC_Os08g44850|13108.t04218 | 1.03862512387888 | -1.63455955339625 |
| LOC_Os08g45110|13108.t04243 | -1.81632454331784 | 0.908174711935608 |
| LOC_Os08g45140|13108.t04246 | 0.195035318846708 | -1.30257779384497 |
| LOC_Os09g03960|13109.t00295 | 2.15496908511635 | -3.04959705267509 |
| LOC_Os09g04100|13109.t00309 | -2.42604314312456 | 0.662057452961926 |
| LOC_Os09g04160|13109.t00315 | 1.15047631935072 | -1.01179501996271 |
| LOC_Os09g06740|13109.t00469 | 1.18255491678825 | -0.687026973290386 |
| LOC_Os09g06770|13109.t00472 | 1.2123022601823 | -1.04959705267509 |
| LOC_Os09g07154|13109.t00511 | 2.08753472435982 | -1.65965053435908 |
| LOC_Os09g08280|13109.t00624 | -3.34674482487897 | 1.29893120311378 |
| LOC_Os09g11440|13109.t00939 | 0.406846871040177 | -1.51426531967854 |
| LOC_Os09g12600|13109.t01055 | 0.185898169727992 | -1.04081452237713 |
| LOC_Os09g13920|13109.t01184 | 1.37435901829127 | -0.984502024453213 |
| LOC_Os09g15480|13109.t01344 | -0.532531577317244 | -1.87556765290004 |
| LOC_Os09g15700|13109.t01367 | 0.872911970530557 | -1.24857808740149 |
| LOC_Os09g16510|13109.t01447 | 1.9264068965397 | -1.71256206539752 |
| LOC_Os09g16520|13109.t01448 | 0.356303745764883 | -1.32970497186783 |
| LOC_Os09g19890|13109.t01727 | -1.43433235132915 | 1.19833046076849 |
| LOC_Os09g19952|13109.t01734 | 1.23451546985326 | 0.392500400475805 |
| LOC_Os09g19954|13109.t01735 | 1.35003899702231 | 0.724014138278164 |
| LOC_Os09g20400|13109.t01780 | -1.16996149793254 | 1.63366752165787 |
| LOC_Os09g20440|13109.t01784 | -2.94200955125419 | 1.35565942581117 |
| LOC_Os09g21120|13109.t01851 | 3.43736881581707 | -0.0495970526750922 |
| LOC_Os09g21919|13109.t01932 | -1.48675848471675 | 0.449472382453824 |
| LOC_Os09g23220|13109.t02011 | 0.699759305085447 | -1.2547114825797 |
| LOC_Os09g23300|13109.t02019 | 1.11349244914019 | -0.24977143467031 |
| LOC_Os09g23740|13109.t02065 | 1.40656561652308 | 0.0114323807224683 |
| LOC_Os09g25380|13109.t02225 | 1.21470235230279 | 0.0743916646003613 |
| LOC_Os09g26160|13109.t02301 | 1.00729191707409 | -0.590165434037794 |
| LOC_Os09g26380|13109.t02323 | -1.36817846370075 | 1.02343000053133 |
| LOC_Os09g26700|13109.t02355 | 1.14231642692111 | 0.204159539570696 |
| LOC_Os09g26900|13109.t02375 | 0.500730876814504 | -1.57684405553996 |
| LOC_Os09g27140|13109.t02400 | 1.02186660561752 | 0.099266333239389 |
| LOC_Os09g27330|13109.t02419 | 2.17817647627768 | -0.0495970526750912 |
| LOC_Os09g27820|13109.t02470 | 0.256830140349907 | -1.20076112600874 |
| LOC_Os09g27830|13109.t02471 | 0.761651527087211 | -1.4605301536212 |
| LOC_Os09g28420|13109.t02527 | 1.18430257907451 | -2.69345324244982 |
| LOC_Os09g28440|13109.t02529 | 1.04787743711708 | -1.55209739320428 |
| LOC_Os09g28520|13109.t02537 | 1.07994203970833 | -1.43488720855988 |
| LOC_Os09g30070|13109.t02685 | 1.26038905684081 | 0.0357944385555493 |
| LOC_Os09g30160|13109.t02694 | 1.41390264335917 | -0.0299682459261615 |
| LOC_Os09g30190|13109.t02697 | 0.511453394526802 | -1.18836112263094 |
| LOC_Os09g30240|13109.t02702 | -1.14640676995364 | 0.480917664023681 |
| LOC_Os09g31040|13109.t02749 | -1.12848486195638 | -2.56417022550485 |
| LOC_Os09g31080|13109.t02752 | 0.549784310806734 | -1.29015087926801 |
| LOC_Os09g31430|13109.t02786 | -2.63944678123376 | -0.371525147562456 |
| LOC_Os09g31490|13109.t02801 | 0.907041571672758 | -1.29752456611868 |
| LOC_Os09g32010|13109.t02817 | -1.0337257203458 | 0.38491031080198 |
| LOC_Os09g32640|13109.t02881 | -0.115884825176754 | -1.26532574373054 |
| LOC_Os09g32988|13109.t02922 | 0.581104293278526 | -1.21309578495797 |
| LOC_Os09g33550|13109.t02934 | -1.55405529000311 | 0.307096460372953 |
| LOC_Os09g33680|13109.t02948 | -4.91246527564017 | 1.65007710692276 |
| LOC_Os09g33876|13109.t02968 | -1.22440928195492 | 1.98993131151154 |
| LOC_Os09g35910|13109.t03075 | 2.00964605690712 | -0.339103669870082 |
| LOC_Os09g36700|13109.t03155 | -2.65352196644548 | 0.615577858423102 |
| LOC_Os09g36740|13109.t03159 | 0.556662152012206 | -1.38746669143148 |
| LOC_Os09g37080|13109.t03193 | 0.917226675055979 | -2.03565786171721 |
| LOC_Os09g37920|13109.t03280 | 1.08599694303723 | 0.163396670659109 |
| LOC_Os09g38330|13109.t03315 | 1.07383981646294 | 0.568532311981266 |
| LOC_Os09g38777|13109.t03361 | 1.33253884259664 | -1.42810867592882 |
| LOC_Os09g39440|13109.t03428 | 1.01778600597756 | -1.81315685685301 |
| LOC_Os09g39730|13109.t03457 | -1.26768753039439 | 0.0310715071995077 |
| LOC_Os10g02770|13110.t00167 | 2.4049473381247 | 0.687368541491116 |
| LOC_Os10g07998|13110.t00615 | 0.10079394496531 | -2.92406617059123 |
| LOC_Os10g09930|13110.t00801 | -1.24430883939262 | -0.173332421097342 |
| LOC_Os10g13700|13110.t01094 | -0.702182536581718 | -3.60418590435273 |
| LOC_Os10g14020|13110.t01121 | 1.75019154134913 | -1.99445549848263 |
| LOC_Os10g14180|13110.t01134 | 1.62460039757311 | -1.15029105229456 |
| LOC_Os10g20450|13110.t01534 | 1.03714619495297 | -1.17297946818037 |
| LOC_Os10g21190|13110.t01605 | 1.09398341284841 | -0.0905273167299771 |
| LOC_Os10g21418|13110.t03614 | 1.09598321402123 | 0.141768473040738 |
| LOC_Os10g24954|13110.t01914 | 1.79351262604235 | -3.37152514756246 |
| LOC_Os10g25000|13110.t01917 | 0.172926215590471 | -1.70670933915208 |
| LOC_Os10g26700|13110.t02073 | -0.244308839392622 | -1.84710418877635 |
| LOC_Os10g28230|13110.t02172 | 0.663840315940792 | -1.01112290486045 |
| LOC_Os10g28240|13110.t02173 | 1.49571280204788 | -0.193986962010267 |
| LOC_Os10g29470|13110.t02287 | -0.392353919268982 | -1.3094641794302 |
| LOC_Os10g29570|13110.t02295 | -1.55698462104179 | 0.581758353530512 |
| LOC_Os10g30560|13110.t02388 | -2.63944678123376 | 1.68736854149111 |
| LOC_Os10g31540|13110.t02473 | 0.680054576259068 | -1.32970497186782 |
| LOC_Os10g31850|13110.t02504 | 0.678155388250815 | -1.87871249347151 |
| LOC_Os10g32050|13110.t02521 | -0.317518686346394 | -1.89759395923004 |
| LOC_Os10g33040|13110.t02603 | 0.315272339465373 | -1.03347738731182 |
| LOC_Os10g33990|13110.t02695 | 1.81998483740354 | 0.0145332847446221 |
| LOC_Os10g35060|13110.t02795 | 1.32178364860167 | -0.796310623392859 |
| LOC_Os10g35460|13110.t02837 | 1.8283830858894 | -1.60832801182307 |
| LOC_Os10g35580|13110.t02849 | 0.693452431474604 | -1.22503905908497 |
| LOC_Os10g35840|13110.t02872 | 1.01500081980869 | -0.569604111555476 |
| LOC_Os10g36170|13110.t02899 | -1.77069131451201 | 1.55612400821286 |
| LOC_Os10g36180|13110.t02900 | -3.18964386379424 | 1.50773432906887 |
| LOC_Os10g38090|13110.t03063 | -1.41149483455541 | 0.271464298394734 |
| LOC_Os10g38470|13110.t03090 | -0.746361985150269 | -1.19398696201027 |
| LOC_Os10g38489|13110.t03091 | -1.00295397987253 | 0.472823981977349 |
| LOC_Os10g39140|13110.t03154 | 0.111011876190267 | -1.10280166984683 |
| LOC_Os10g39610|13110.t03200 | -1.61101984704641 | 0.893855682307747 |
| LOC_Os10g39660|13110.t03203 | 1.62358762460003 | -0.993013524308724 |
| LOC_Os10g39750|13110.t03213 | 0.0131544363627553 | -1.01538133733718 |
| LOC_Os10g40360|13110.t03274 | 1.86534537080341 | -1.79961879966674 |
| LOC_Os10g40530|13110.t03289 | 0.18722161588068 | -1.25395555118128 |
| LOC_Os10g40550|13110.t03291 | 2.24097160301358 | 0.348952323815185 |
| LOC_Os10g40640|13110.t03303 | 1.66172275348681 | -1.37152514756245 |
| LOC_Os10g41330|13110.t03366 | 2.14345509709931 | -0.230169298316915 |
| LOC_Os10g41550|13110.t03386 | 1.92294530791206 | -2.21952205411741 |
| LOC_Os10g41710|13110.t03398 | 1.07383981646294 | -0.775422089236098 |
| LOC_Os10g42410|13110.t03462 | 1.46907767554441 | 0.223421441731325 |
| LOC_Os10g42660|13110.t03487 | 0.159640524840243 | -1.20160014612014 |
| LOC_Os11g02080|13111.t00104 | -0.660699330462359 | -1.90899872250229 |
| LOC_Os11g02240|13111.t00120 | 1.54846396568191 | -1.86083540921993 |
| LOC_Os11g02290|13111.t00125 | 0.723123298150952 | -1.32643725803391 |
| LOC_Os11g02330|13111.t00129 | 1.0499752716776 | -1.66662076262855 |
| LOC_Os11g02350|13111.t00131 | -0.116394881763597 | -1.15500929256268 |
| LOC_Os11g02379|13111.t00134 | 2.16790814082385 | -2.95648764828361 |
| LOC_Os11g02389|13111.t00135 | -1.34358666821745 | 0.198043176235157 |
| LOC_Os11g02400|13111.t00136 | -1.64866531126314 | 0.29240681897693 |
| LOC_Os11g02424|13111.t00138 | -1.39076766837703 | 0.314559277601568 |
| LOC_Os11g02440|13111.t00139 | -1.75080789018146 | -0.523528241007508 |
| LOC_Os11g03700|13111.t00263 | -1.41203628513068 | 1.33431792910797 |
| LOC_Os11g05160|13111.t00415 | 0.461913933725483 | -1.30898368149374 |
| LOC_Os11g05410|13111.t00441 | -1.7065609770923 | 1.53536544804606 |
| LOC_Os11g05730|13111.t00474 | 1.00440940854097 | -1.38663203995266 |
| LOC_Os11g06440|13111.t00546 | 1.49440896550103 | -1.2195220541174 |
| LOC_Os11g07600|13111.t00663 | -1.49742777636133 | 0.165670934227975 |
| LOC_Os11g07911|13111.t00695 | -1.52263311625101 | 0.433344511105554 |
| LOC_Os11g07960|13111.t00702 | 0.673143449074266 | -2.26682776889576 |
| LOC_Os11g08210|13111.t00726 | 1.29741887382025 | -0.51601993822085 |
| LOC_Os11g10510|13111.t00951 | 0.0641602159860219 | -2.76384257034121 |
| LOC_Os11g10590|13111.t00959 | -2.98355871630593 | 1.27130676453993 |
| LOC_Os11g10750|13111.t00973 | 1.16994728609744 | -0.394925580142535 |
| LOC_Os11g11370|13111.t01034 | 1.17080803132964 | -0.199344172179771 |
| LOC_Os11g12710|13111.t01165 | -1.27687670184905 | 0.659623551008215 |
| LOC_Os11g13570|13111.t01198 | 1.3136668311354 | -3.89089930665604 |
| LOC_Os11g16550|13111.t01446 | 0.885255194669922 | -1.47340476158167 |
| LOC_Os11g16924|13111.t01482 | 2.29129055632913 | -0.175127934758951 |
| LOC_Os11g18570|13111.t01629 | -1.3212708212075 | 1.23051086651764 |
| LOC_Os11g20790|13111.t01844 | 0.772742788256622 | -1.01499839115112 |
| LOC_Os11g24070|13111.t02028 | -2.90863341404915 | 1.41818190867572 |
| LOC_Os11g25860|13111.t02196 | -0.334592199705337 | -1.50479167842592 |
| LOC_Os11g26570|13111.t02267 | -2.02106127897516 | 0.583852322038395 |
| LOC_Os11g26750|13111.t02284 | -2.46952177979144 | 1.29617778416664 |
| LOC_Os11g26760|13111.t02285 | -1.91246527564018 | 0.985892032043531 |
| LOC_Os11g26790|13111.t02288 | -1.50567356348166 | 0.989679567419169 |
| LOC_Os11g31880|13111.t02780 | 1.32055515083432 | 0.757757869382512 |
| LOC_Os11g31900|13111.t02782 | 1.54786029843679 | -1.09399117203354 |
| LOC_Os11g31980|13111.t02790 | 0.205274993288334 | -1.226901584483 |
| LOC_Os11g32650|13111.t02857 | -2.49058339531928 | 0.693627531962792 |
| LOC_Os11g32890|13111.t02881 | -2.77069131451201 | 1.81054528374194 |
| LOC_Os11g33100|13111.t02902 | 1.12650802483254 | 0.0336584509742544 |
| LOC_Os11g35400|13111.t03086 | 1.07676025276565 | 0.0636135577729049 |
| LOC_Os11g37100|13111.t03251 | 0.660113500625156 | -1.52764434947973 |
| LOC_Os11g37270|13111.t03267 | -1.51140110772775 | 0.534154989254269 |
| LOC_Os11g40500|13111.t03584 | 1.07040696731521 | -0.11233280802306 |
| LOC_Os11g40590|13111.t03593 | 1.20060517864329 | -2.32675527744243 |
| LOC_Os12g01770|13112.t00075 | 1.07204812541633 | -0.284062306312116 |
| LOC_Os12g02040|13112.t00102 | -0.690072854303727 | -1.56180321096094 |
| LOC_Os12g02200|13112.t00118 | 1.14916346343514 | -1.35854729356425 |
| LOC_Os12g02240|13112.t00122 | 0.262705895368097 | -1.89287154898764 |
| LOC_Os12g02290|13112.t00127 | 0.57768074160745 | -1.02737688783353 |
| LOC_Os12g02320|13112.t00130 | -1.10575432086939 | 0.372976190411342 |
| LOC_Os12g02340|13112.t00132 | -1.67463620969606 | 0.494226753252577 |
| LOC_Os12g02370|13112.t00134 | -1.32367091332799 | -0.264335901468106 |
| LOC_Os12g02570|13112.t00154 | 1.87625305705028 | -0.242242130617485 |
| LOC_Os12g03530|13112.t00246 | -1.37641237539996 | 0.664098762168268 |
| LOC_Os12g03540|13112.t00247 | 1.04935153047193 | -0.415246524991773 |
| LOC_Os12g04240|13112.t00315 | 1.20725680829967 | -0.129324245145832 |
| LOC_Os12g04260|13112.t00317 | 0.466347882788839 | 1.12728070940898 |
| LOC_Os12g04440|13112.t00334 | 0.9455157194874 | -1.14270645706658 |
| LOC_Os12g05120|13112.t00402 | 0.0069162641515420 | -1.22266176164797 |
| LOC_Os12g05210|13112.t00412 | -2.47247509718079 | 1.00468681666883 |
| LOC_Os12g06780|13112.t00563 | 1.16342074983154 | -1.18084158595334 |
| LOC_Os12g06870|13112.t00572 | 1.13107137264348 | -0.7912850570837 |
| LOC_Os12g07280|13112.t00611 | -1.63944678123376 | 1.2333369105964 |
| LOC_Os12g07820|13112.t00664 | 1.14904911357253 | -0.924066170591234 |
| LOC_Os12g08130|13112.t00697 | 1.87840152362886 | -0.701673749254789 |
| LOC_Os12g12880|13112.t01158 | 0.0285714592149554 | -1.2195220541174 |
| LOC_Os12g13120|13112.t01181 | 2.06099293690734 | -0.634559553396245 |
| LOC_Os12g13445|13112.t04165 | -1.0544842805126 | 3.03786578857525 |
| LOC_Os12g13570|13112.t01226 | 0.763138976998828 | -1.12359763411887 |
| LOC_Os12g19381|13112.t01779 | 1.69274964910743 | -1.0495970526751 |
| LOC_Os12g24020|13112.t02131 | 1.38608831087338 | -4.80548733171976 |
| LOC_Os12g25630|13112.t02284 | 0.733255880886731 | -1.24121712521672 |
| LOC_Os12g27830|13112.t02499 | -1.15817969502118 | 0.39129057202165 |
| LOC_Os12g29760|13112.t02692 | 0.231633738434233 | -1.04031923908709 |
| LOC_Os12g31850|13112.t02892 | -1.57804623656962 | 1.20820070479255 |
| LOC_Os12g32580|13112.t02960 | 1.752870641545 | 0.312973026709615 |
| LOC_Os12g34018|13112.t04154 | 1.12483941893197 | 0.110867619518153 |
| LOC_Os12g34860|13112.t03186 | 1.17292621559047 | -0.0852209624058165 |
| LOC_Os12g36210|13112.t03320 | -1.28036568792893 | -0.698689890815963 |
| LOC_Os12g36220|13112.t03321 | -1.44657935682549 | -0.741611602941592 |
| LOC_Os12g36240|13112.t03323 | -1.48744368778871 | -0.620484368184525 |
| LOC_Os12g36910|13112.t03386 | 1.31702584322532 | -0.980649698957602 |
| LOC_Os12g37320|13112.t03427 | -2.97096092495032 | 1.60378191121814 |
| LOC_Os12g39860|13112.t03675 | 1.28360863380577 | -0.274531668573994 |
| LOC_Os12g40180|13112.t03707 | 2.1639392386209 | -2.58011176937388 |
| LOC_Os12g42400|13112.t03929 | 1.34761416305874 | -0.0495970526750944 |
| LOC_Os12g43140|13112.t04001 | -2.24712935845499 | 1.46610278560895 |
| LOC_Os12g43340|13112.t04018 | -1.67897514542039 | 0.0538325369803132 |
| LOC_Os12g44190|13112.t04101 | -3.22440928195491 | 2.17279536866135 |
| LOC_Os12g44270|13112.t04109 | -1.30824087275839 | 0.566062245268978 |
