# Supplementary material for: Transcriptomic Analysis of Rice (Oryza sativa) Developing Embryos Using the RNA-Seq Technique
Source: PLoS One. 2012 Feb 8;7(2):e30646. doi: 10.1371/journal.pone.0030646 (PMC3275597; doi:10.1371/journal.pone.0030646)
Supplement: Table S2 — Differentially expressed genes between R1 and R2. FDR: false discovery rate. We used FDR<0.001 and the absolute value of log2Ratio≥1 as the threshold to judge the significance of gene expression difference. (DOC) [file pone.0030646.s002.doc]

**Table S2** Differentially expressed genes between R1 and R2. FDR: false discovery rate. We used FDR < 0.001 and the absolute value of log_2_Ratio≥1 as the threshold to judge the significance of gene expression difference.

| ***Gene ID*** | ***Description*** | ***Up-Down-***  ***Regulation***  ***(R2/R1)*** | ***Gene length***  ***(bp)*** | ***FDR*** |
| --- | --- | --- | --- | --- |
| LOC_Os01g03890 | DUF260 domain containing protein, putative, expressed | Down | 985 | 3.71E-05 |
| LOC_Os09g07130 | retrotransposon protein, putative, unclassified | Down | 1536 | 0.000232 |
| LOC_Os02g49370 | histone-like transcription factor and archaeal histone, putative, expressed | Down | 1169 | 5.75E-05 |
| LOC_Os09g21120 | armadillo/beta-catenin repeat family protein, putative, expressed | Down | 2592 | 0.000438 |
| LOC_Os04g29274 | expressed protein | Down | 872 | 0.000274 |
| LOC_Os07g23570 | cytochrome P450 72A1, putative, expressed | Down | 2009 | 0.000274 |
| LOC_Os02g05430 | expressed protein | Down | 2860 | 0.000885 |
| LOC_Os01g59680 | NHL25, putative, expressed | Down | 1455 | 0.000596 |
| LOC_Os07g07530 | expressed protein | Down | 1992 | 0.000169 |
| LOC_Os01g08380 | transferase family protein, putative, expressed | Down | 1748 | 4.22E-09 |
| LOC_Os01g54340 | plant-specific domain TIGR01615 family protein, expressed | Down | 1323 | 0 |
| LOC_Os01g03690 | TKL_IRAK_DUF26-lg.1 - DUF26 kinases have homology to DUF26 containing loci, expressed | Down | 3298 | 3.35E-05 |
| LOC_Os02g16540 | OsWRKY39v2 - Superfamily of TFs having WRKY and zinc finger domains, expressed | Down | 2272 | 4.98E-08 |
| LOC_Os07g10810 | conserved hypothetical protein | Down | 1152 | 6.66E-11 |
| LOC_Os10g02770 | glycosyl hydrolases family 16, putative, expressed | Down | 1670 | 0.000308 |
| LOC_Os04g51130 | expressed protein | Down | 1666 | 6.65E-05 |
| LOC_Os01g56220 | expressed protein | Down | 830 | 0.0004 |
| LOC_Os05g35140 | nodulin MtN3 family protein, putative, expressed | Down | 1359 | 9.13E-08 |
| LOC_Os02g49840 | OsMADS57 - MADS-box family gene with MIKCc type-box, expressed | Down | 1384 | 7.23E-05 |
| LOC_Os08g31850 | expressed protein | Down | 1552 | 3.72E-05 |
| LOC_Os07g20420 | tetratricopeptide-like helical, putative, expressed | Down | 2401 | 0.000545 |
| LOC_Os01g04280 | calmodulin binding protein, putative, expressed | Down | 1953 | 2.51E-11 |
| LOC_Os06g22919 | DEFL9 - Defensin and Defensin-like DEFL family, expressed | Down | 4043 | 1.46E-08 |
| LOC_Os11g16924 | expressed protein | Down | 2619 | 6.14E-06 |
| LOC_Os03g61240 | expressed protein | Down | 1088 | 0.000777 |
| LOC_Os10g40550 | CPuORF23 - conserved peptide uORF-containing transcript, expressed | Down | 2027 | 1.46E-08 |
| LOC_Os05g36270 | fructose-1,6-bisphosphatase, putative, expressed | Down | 1600 | 2.26E-09 |
| LOC_Os08g39694 | cytochrome P450, putative, expressed | Down | 2787 | 7.84E-09 |
| LOC_Os03g55240 | cytochrome P450, putative, expressed | Down | 2545 | 0 |
| LOC_Os06g04240 | expressed protein | Down | 731 | 6.83E-06 |
| LOC_Os09g27330 | oxidoreductase/ transition metal ion binding protein, putative, expressed | Down | 746 | 3.08E-05 |
| LOC_Os11g02379 | LTPL6 - Protease inhibitor/seed storage/LTP family protein precursor, expressed | Down | 1480 | 1.08E-06 |
| LOC_Os12g40180 | expressed protein | Down | 625 | 9.84E-13 |
| LOC_Os02g36530 | hypothetical protein | Down | 267 | 4.98E-08 |
| LOC_Os03g55776 | expressed protein | Down | 849 | 0 |
| LOC_Os09g03960 | expressed protein | Down | 764 | 0.00023 |
| LOC_Os07g02790 | expressed protein | Down | 774 | 3.80E-05 |
| LOC_Os10g41330 | AP2 domain containing protein, expressed | Down | 1183 | 3.19E-07 |
| LOC_Os06g46140 | expressed protein | Down | 1534 | 0.000331 |
| LOC_Os08g38710 | uncharacterized glycosyltransferase, putative, expressed | Down | 2727 | 1.76E-07 |
| LOC_Os09g07154 | hypothetical protein | Down | 1110 | 1.57E-12 |
| LOC_Os12g13120 | histidine triad family protein, putative, expressed | Down | 1283 | 0.000214 |
| LOC_Os08g36910 | alpha-amylase precursor, putative, expressed | Down | 2770 | 0 |
| LOC_Os02g09990 | expressed protein | Down | 1317 | 6.26E-12 |
| LOC_Os05g33820 | lipase, putative, expressed | Down | 2059 | 3.33E-06 |
| LOC_Os04g48270 | OsFBX148 - F-box domain containing protein, expressed | Down | 1797 | 4.19E-06 |
| LOC_Os01g72530 | OsCML31 - Calmodulin-related calcium sensor protein, expressed | Down | 999 | 5.69E-08 |
| LOC_Os06g07600 | uncharacterized glycosyltransferase, putative, expressed | Down | 2952 | 0 |
| LOC_Os09g35910 | homeobox associated leucine zipper, putative, expressed | Down | 1435 | 6.02E-05 |
| LOC_Os04g02530 | expressed protein | Down | 2899 | 2.14E-11 |
| LOC_Os01g04670 | expressed protein | Down | 799 | 0 |
| LOC_Os08g15296 | photosystem II reaction center protein H, putative, expressed | Down | 949 | 0.000466 |
| LOC_Os03g15270 | gibberellin receptor GID1L2, putative, expressed | Down | 1703 | 4.17E-12 |
| LOC_Os06g50230 | expressed protein | Down | 1189 | 5.12E-12 |
| LOC_Os09g16510 | OsWRKY74 - Superfamily of TFs having WRKY and zinc finger domains, expressed | Down | 1774 | 2.64E-07 |
| LOC_Os10g41550 | beta-amylase, putative, expressed | Down | 2130 | 0 |
| LOC_Os01g40190 | retrotransposon protein, putative, unclassified, expressed | Down | 2687 | 4.24E-06 |
| LOC_Os03g31679 | annexin A7, putative, expressed | Down | 892 | 9.69E-08 |
| LOC_Os03g49440 | phosphatase, putative, expressed | Down | 1225 | 5.67E-12 |
| LOC_Os12g08130 | amino acid transporter, putative, expressed | Down | 1882 | 3.22E-11 |
| LOC_Os12g02570 | expressed protein | Down | 2509 | 6.13E-06 |
| LOC_Os02g15280 | VQ domain containing protein, putative, expressed | Down | 468 | 0 |
| LOC_Os10g40360 | proline oxidase, mitochondrial precursor, putative, expressed | Down | 2075 | 0 |
| LOC_Os03g55540 | ZOS3-18 - C2H2 zinc finger protein, expressed | Down | 1285 | 0 |
| LOC_Os01g04050 | BBTI12 - Bowman-Birk type bran trypsin inhibitor precursor, expressed | Down | 804 | 0 |
| LOC_Os03g02550 | OsFBX76 - F-box domain containing protein, expressed | Down | 1736 | 0 |
| LOC_Os03g44540 | nuclear transcription factor Y subunit, putative, expressed | Down | 2188 | 0 |
| LOC_Os05g44060 | expressed protein | Down | 846 | 4.61E-12 |
| LOC_Os10g35460 | COBRA, putative, expressed | Down | 1617 | 0 |
| LOC_Os10g33990 | DUF584 domain containing protein, putative, expressed | Down | 534 | 0 |
| LOC_Os07g10460 | 5-nucleotidase surE, putative, expressed | Down | 1274 | 2.91E-12 |
| LOC_Os06g23800 | flavin-containing monooxygenase family protein, putative, expressed | Down | 1210 | 7.30E-05 |
| LOC_Os01g69850 | OsMADS65 - MADS-box family gene with MIKC* type-box, expressed | Down | 1318 | 0 |
| LOC_Os05g44290 | protein kinase domain containing protein, expressed | Down | 3778 | 6.35E-12 |
| LOC_Os10g24954 | ulp1 protease family, C-terminal catalytic domain containing protein | Down | 2955 | 1.46E-06 |
| LOC_Os02g44230 | CPuORF22 - conserved peptide uORF-containing transcript, expressed | Down | 1833 | 2.97E-05 |
| LOC_Os04g58890 | expressed protein | Down | 1460 | 6.49E-08 |
| LOC_Os01g12110 | expressed protein | Down | 927 | 2.91E-09 |
| LOC_Os03g60570 | ZOS3-22 - C2H2 zinc finger protein, expressed | Down | 915 | 1.83E-09 |
| LOC_Os12g32580 | expressed protein | Down | 885 | 0.000162 |
| LOC_Os02g54730 | transmembrane amino acid transporter protein, putative, expressed | Down | 2181 | 8.66E-07 |
| LOC_Os10g14020 | TPD1, putative, expressed | Down | 980 | 0 |
| LOC_Os01g15000 | lipase, putative, expressed | Down | 2738 | 0 |
| LOC_Os03g16670 | haloacid dehalogenase-like hydrolase family protein, putative, expressed | Down | 1377 | 6.95E-12 |
| LOC_Os01g16250 | expressed protein | Down | 756 | 0 |
| LOC_Os03g46440 | BTBA4 - Bric-a-Brac,Tramtrack, Broad Complex BTB domain with Ankyrin repeat region, expressed | Down | 3770 | 0 |
| LOC_Os01g43480 | AAA-type ATPase family protein, putative, expressed | Down | 3476 | 2.15E-11 |
| LOC_Os02g41840 | DUF584 domain containing protein, putative, expressed | Down | 1067 | 7.41E-13 |
| LOC_Os03g51080 | glutamate decarboxylase, putative, expressed | Down | 2184 | 7.01E-06 |
| LOC_Os01g09220 | transposon protein, putative, CACTA, En/Spm sub-class, expressed | Down | 1834 | 1.38E-10 |
| LOC_Os04g56610 | expressed protein | Down | 201 | 5.35E-08 |
| LOC_Os02g54980 | pheophorbide a oxygenase, chloroplast precursor, putative, expressed | Down | 2128 | 4.73E-07 |
| LOC_Os12g19381 | ribulose bisphosphate carboxylase small chain, chloroplast precursor, putative, expressed | Down | 1112 | 0.000353 |
| LOC_Os04g52260 | LTPL124 - Protease inhibitor/seed storage/LTP family protein precursor, expressed | Down | 1022 | 6.04E-05 |
| LOC_Os03g18770 | wound-induced protein WI12, putative, expressed | Down | 1254 | 1.75E-12 |
| LOC_Os07g47960 | basic helix-loop-helix domain containing protein, expressed | Down | 1072 | 4.99E-08 |
| LOC_Os06g34780 | expressed protein | Down | 696 | 6.52E-06 |
| LOC_Os05g46760 | STE_MEKK_ste11_MAP3K.19 - STE kinases include homologs to sterile 7, sterile 11 and sterile 20 from yeast, expressed | Down | 1419 | 2.15E-11 |
| LOC_Os06g39370 | OsFBK16 - F-box domain and kelch repeat containing protein, expressed | Down | 1707 | 0 |
| LOC_Os04g54210 | hypothetical protein | Down | 285 | 2.07E-09 |
| LOC_Os03g10620 | hydrolase, alpha/beta fold family domain containing protein, expressed | Down | 2003 | 1.52E-05 |
| LOC_Os02g41954 | gibberellin 2-beta-dioxygenase 7, putative, expressed | Down | 1660 | 8.84E-05 |
| LOC_Os04g01230 | phosphoglycerate mutase, putative, expressed | Down | 1596 | 9.59E-06 |
| LOC_Os10g40640 | glycosyl transferase 8 domain containing protein, putative, expressed | Down | 2374 | 3.05E-09 |
| LOC_Os03g09230 | LTPL69 - Protease inhibitor/seed storage/LTP family protein precursor, expressed | Down | 1157 | 5.59E-05 |
| LOC_Os04g45970 | glutamate dehydrogenase protein, putative, expressed | Down | 2101 | 1.43E-12 |
| LOC_Os04g16830 | DNA-directed RNA polymerase subunit beta, putative | Down | 1797 | 3.83E-06 |
| LOC_Os02g11070 | 3-ketoacyl-CoA synthase, putative, expressed | Down | 2054 | 2.50E-08 |
| LOC_Os03g21640 | expressed protein | Down | 2753 | 0 |
| LOC_Os10g14180 | expressed protein | Down | 7259 | 1.76E-12 |
| LOC_Os10g39660 | expressed protein | Down | 1608 | 2.21E-06 |
| LOC_Os08g39050 | pentatricopeptide, putative, expressed | Down | 2338 | 0.000301 |
| LOC_Os07g43540 | ORC6 - Putative origin recognition complex subunit 6, expressed | Down | 1101 | 0.000189 |
| LOC_Os06g41030 | DUF1680 domain containing protein, putative, expressed | Down | 3020 | 4.89E-12 |
| LOC_Os05g13580 | OsCML18 - Calmodulin-related calcium sensor protein, expressed | Down | 1031 | 0.000119 |
| LOC_Os03g03724 | expressed protein | Down | 716 | 1.21E-08 |
| LOC_Os07g37400 | OsFBX257 - F-box domain containing protein, expressed | Down | 1166 | 0 |
| LOC_Os04g49550 | RING-H2 finger protein ATL2A, putative, expressed | Down | 1119 | 1.72E-05 |
| LOC_Os03g53020 | helix-loop-helix DNA-binding domain containing protein, expressed | Down | 1286 | 0 |
| LOC_Os02g02400 | catalase isozyme A, putative, expressed | Down | 2421 | 8.17E-12 |
| LOC_Os01g64470 | harpin-induced protein 1 domain containing protein, expressed | Down | 1012 | 0.000627 |
| LOC_Os03g53930 | expressed protein | Down | 1222 | 0.000627 |
| LOC_Os08g14400 | homeobox domain containing protein, expressed | Down | 1250 | 0.000398 |
| LOC_Os04g46980 | cis-zeatin O-glucosyltransferase, putative, expressed | Down | 1753 | 1.51E-10 |
| LOC_Os05g49160 | expressed protein | Down | 948 | 9.72E-11 |
| LOC_Os06g12150 | shikimate kinase, putative, expressed | Down | 1921 | 6.21E-06 |
| LOC_Os11g02240 | CAMK_KIN1/SNF1/Nim1_like.4 - CAMK includes calcium/calmodulin depedent protein kinases, expressed | Down | 2121 | 1.17E-11 |
| LOC_Os11g31900 | acyl carrier protein, putative, expressed | Down | 1072 | 0 |
| LOC_Os04g51090 | tRNA-splicing endonuclease positive effector-related, putative, expressed | Down | 8043 | 2.41E-07 |
| LOC_Os06g19444 | CCT/B-box zinc finger protein, putative, expressed | Down | 2298 | 3.72E-08 |
| LOC_Os01g04920 | glycosyl transferase, group 1 domain containing protein, expressed | Down | 1891 | 0 |
| LOC_Os08g38460 | zinc finger, C3HC4 type domain containing protein, expressed | Down | 1016 | 1.41E-06 |
| LOC_Os02g32580 | expressed protein | Down | 1101 | 3.61E-13 |
| LOC_Os06g36560 | inositol oxygenase, putative, expressed | Down | 1797 | 3.08E-11 |
| LOC_Os05g43170 | calreticulin precursor protein, putative, expressed | Down | 1888 | 4.78E-11 |
| LOC_Os01g60730 | RING-H2 finger protein, putative, expressed | Down | 1349 | 5.15E-05 |
| LOC_Os05g39720 | OsWRKY70 - Superfamily of TFs having WRKY and zinc finger domains, expressed | Down | 2273 | 8.12E-05 |
| LOC_Os02g36700 | sucrose transporter BoSUT1, putative, expressed | Down | 2044 | 0.000325 |
| LOC_Os02g05890 | EMB1303, putative, expressed | Down | 1220 | 0.000818 |
| LOC_Os01g07970 | expressed protein | Down | 1015 | 1.16E-06 |
| LOC_Os03g51390 | expressed protein | Down | 1166 | 1.16E-06 |
| LOC_Os01g49320 | glycosyl hydrolase, putative, expressed | Down | 1339 | 1.83E-06 |
| LOC_Os10g28240 | calcium-transporting ATPase, plasma membrane-type, putative, expressed | Down | 3377 | 1.57E-08 |
| LOC_Os11g06440 | expressed protein | Down | 1855 | 1.17E-05 |
| LOC_Os04g48350 | dehydration-responsive element-binding protein, putative, expressed | Down | 1053 | 7.57E-10 |
| LOC_Os05g47960 | expressed protein | Down | 818 | 0.000463 |
| LOC_Os02g06930 | protein kinase, putative, expressed | Down | 2078 | 0 |
| LOC_Os07g48710 | VQ domain containing protein, putative, expressed | Down | 1160 | 5.86E-07 |
| LOC_Os10g42410 | zinc-binding protein, putative, expressed | Down | 2394 | 6.61E-05 |
| LOC_Os05g48930 | OsGrx_S2 - glutaredoxin subgroup III, expressed | Down | 945 | 3.33E-07 |
| LOC_Os07g04450 | expressed protein | Down | 758 | 5.25E-07 |
| LOC_Os01g55030 | DNA-binding protein-related, putative, expressed | Down | 990 | 0 |
| LOC_Os04g44510 | GEM, putative, expressed | Down | 1075 | 1.93E-10 |
| LOC_Os06g06350 | AMP-binding enzyme, putative, expressed | Down | 2284 | 4.73E-07 |
| LOC_Os08g19140 | adenylate kinase family protein, putative, expressed | Down | 2279 | 0.000149 |
| LOC_Os02g07180 | expressed protein | Down | 1699 | 0.000149 |
| LOC_Os05g49860 | Core histone H2A/H2B/H3/H4 domain containing protein, putative, expressed | Down | 826 | 1.11E-11 |
| LOC_Os03g55430 | expressed protein | Down | 921 | 5.31E-05 |
| LOC_Os01g54550 | HSF-type DNA-binding domain containing protein, expressed | Down | 1953 | 1.20E-05 |
| LOC_Os04g49520 | pentatricopeptide repeat protein PPR868-14, putative, expressed | Down | 3655 | 3.32E-08 |
| LOC_Os02g01970 | expressed protein | Down | 2547 | 0.000934 |
| LOC_Os02g09980 | expressed protein | Down | 917 | 4.76E-05 |
| LOC_Os02g44870 | dehydrin, putative, expressed | Down | 1371 | 0 |
| LOC_Os09g30160 | zinc finger, C3HC4 type domain containing protein, expressed | Down | 965 | 0 |
| LOC_Os09g23740 | 1-phosphatidylinositol-4-phosphate 5-kinase/ zinc ion binding protein, putative, expressed | Down | 5435 | 0 |
| LOC_Os02g46210 | DUF581 domain containing protein, expressed | Down | 1220 | 9.54E-06 |
| LOC_Os07g37030 | cytochrome b6-f complex iron-sulfur subunit, chloroplast precursor, putative, expressed | Down | 1252 | 0.000188 |
| LOC_Os12g24020 | rhodanese-like domain containing protein, putative, expressed | Down | 1542 | 2.28E-09 |
| LOC_Os08g31980 | trehalose-6-phosphate synthase, putative, expressed | Down | 3289 | 0 |
| LOC_Os04g49450 | MYB family transcription factor, putative, expressed | Down | 2089 | 0 |
| LOC_Os04g09800 | SWI/SNF-related matrix-associated actin-dependent regulator of chromatin subfamily A, putative | Down | 3399 | 9.35E-05 |
| LOC_Os01g65650 | receptor-like protein kinase HAIKU2 precursor, putative, expressed | Down | 4110 | 9.23E-09 |
| LOC_Os04g33740 | glycosyl hydrolases, putative, expressed | Down | 2228 | 1.51E-06 |
| LOC_Os09g13920 | expressed protein | Down | 1268 | 1.39E-10 |
| LOC_Os07g44140 | cytochrome P450 72A1, putative, expressed | Down | 2285 | 0 |
| LOC_Os01g62900 | amino acid kinase, putative, expressed | Down | 3441 | 2.91E-12 |
| LOC_Os02g16500 | expressed protein | Down | 222 | 9.19E-12 |
| LOC_Os01g47760 | OsGrx_I1 - glutaredoxin subgroup III, expressed | Down | 981 | 1.09E-11 |
| LOC_Os02g09810 | amino acid transporter, putative, expressed | Down | 1874 | 1.67E-05 |
| LOC_Os04g21110 | phosphoribulokinase/Uridine kinase family protein, expressed | Down | 3403 | 2.65E-07 |
| LOC_Os08g04460 | NADPH-dependent FMN reductase domain containing protein, expressed | Down | 1055 | 2.72E-10 |
| LOC_Os06g45140 | bZIP transcription factor domain containing protein, expressed | Down | 1876 | 1.91E-12 |
| LOC_Os05g25770 | OsWRKY45 - Superfamily of TFs having WRKY and zinc finger domains, expressed | Down | 1528 | 0 |
| LOC_Os09g19954 | ribosomal protein, putative | Down | 2145 | 3.88E-12 |
| LOC_Os12g42400 | nuclear transcription factor Y subunit, putative, expressed | Down | 1751 | 9.03E-09 |
| LOC_Os02g33149 | OR, putative, expressed | Down | 1351 | 2.35E-05 |
| LOC_Os05g34270 | inactive receptor kinase At1g27190 precursor, putative, expressed | Down | 2711 | 9.74E-10 |
| LOC_Os03g29190 | PDI, putative, expressed | Down | 2198 | 1.41E-11 |
| LOC_Os01g14850 | MFS18 protein precursor, putative, expressed | Down | 1009 | 1.64E-11 |
| LOC_Os06g45640 | core histone H2A/H2B/H3/H4, putative, expressed | Down | 1440 | 0.000183 |
| LOC_Os01g66850 | pectinacetylesterase domain containing protein, expressed | Down | 1209 | 0 |
| LOC_Os09g38777 | MOSC domain-containing protein, mitochondrial precursor, putative, expressed | Down | 1261 | 0.00029 |
| LOC_Os02g36340 | riboflavin biosynthesis protein ribAB, chloroplast precursor, putative, expressed | Down | 2247 | 0 |
| LOC_Os10g35060 | Ser/Thr protein phosphatase family protein, putative, expressed | Down | 1502 | 0 |
| LOC_Os11g31880 | phosphoglycerate mutase, putative, expressed | Down | 1618 | 5.12E-05 |
| LOC_Os12g36910 | calmodulin binding protein, putative, expressed | Down | 2368 | 0 |
| LOC_Os05g41670 | ICE-like protease p20 domain containing protein, putative, expressed | Down | 1619 | 0.000713 |
| LOC_Os11g13570 | gibberellin receptor GID1L2, putative, expressed | Down | 1522 | 1.80E-06 |
| LOC_Os02g08440 | OsWRKY71 - Superfamily of TFs having WRKY and zinc finger domains, expressed | Down | 1987 | 1.43E-12 |
| LOC_Os08g21541 | expressed protein | Down | 4751 | 0.000627 |
| LOC_Os04g30420 | dehydrogenase, putative, expressed | Down | 1426 | 1.75E-12 |
| LOC_Os03g01770 | rhodanese, putative, expressed | Down | 729 | 0.000353 |
| LOC_Os11g08210 | no apical meristem protein, putative, expressed | Down | 1998 | 3.34E-12 |
| LOC_Os01g68650 | plant-specific domain TIGR01615 family protein, expressed | Down | 1123 | 0 |
| LOC_Os06g43810 | expressed protein | Down | 666 | 1.41E-09 |
| LOC_Os03g55590 | MYB family transcription factor, putative, expressed | Down | 2367 | 0 |
| LOC_Os03g20460 | guanylate kinase, putative, expressed | Down | 1443 | 0.000553 |
| LOC_Os01g67850 | zinc finger, RING-type, putative, expressed | Down | 1373 | 4.15E-11 |
| LOC_Os12g39860 | phosphoribosyl transferase, putative, expressed | Down | 1222 | 0 |
| LOC_Os02g45930 | expressed protein | Down | 1113 | 2.04E-11 |
| LOC_Os04g45370 | OsSAUR19 - Auxin-responsive SAUR gene family member, expressed | Down | 863 | 0.000866 |
| LOC_Os03g48390 | expressed protein | Down | 264 | 0.000485 |
| LOC_Os03g52680 | expressed protein | Down | 1184 | 2.73E-09 |
| LOC_Os08g36920 | AP2 domain containing protein, expressed | Down | 1122 | 4.72E-11 |
| LOC_Os01g64640 | histone H3, putative, expressed | Down | 819 | 2.32E-11 |
| LOC_Os05g07220 | kelch repeat-containing protein, putative, expressed | Down | 3840 | 4.25E-05 |
| LOC_Os01g08600 | expressed protein | Down | 816 | 0 |
| LOC_Os08g40620 | rabGAP/TBC domain-containing protein, putative, expressed | Down | 3483 | 0 |
| LOC_Os01g64900 | HEAT, putative, expressed | Down | 5074 | 2.00E-07 |
| LOC_Os09g30070 | expressed protein | Down | 5035 | 8.79E-11 |
| LOC_Os08g01610 | DUF250 domain containing protein, putative, expressed | Down | 2071 | 0 |
| LOC_Os01g36950 | N-rich protein, putative, expressed | Down | 1771 | 5.31E-12 |
| LOC_Os01g29150 | cytochrome P450 72A1, putative, expressed | Down | 2143 | 3.04E-08 |
| LOC_Os01g53620 | expressed protein | Down | 654 | 0.000375 |
| LOC_Os08g35190 | auxin-repressed protein, putative, expressed | Down | 1502 | 3.29E-05 |
| LOC_Os07g05390 | expressed protein | Down | 3139 | 2.23E-09 |
| LOC_Os01g49370 | expressed protein | Down | 981 | 5.09E-06 |
| LOC_Os02g05680 | apoptosis-inducing factor 2, putative, expressed | Down | 2423 | 1.22E-06 |
| LOC_Os08g44270 | vignain precursor, putative, expressed | Down | 1458 | 8.73E-09 |
| LOC_Os01g59350 | transcription factor, putative, expressed | Down | 3161 | 0 |
| LOC_Os07g14310 | expressed protein | Down | 1822 | 0.000915 |
| LOC_Os09g19952 | ribosomal protein L22, putative | Down | 2455 | 0 |
| LOC_Os03g17940 | phosphatase, putative, expressed | Down | 1198 | 2.21E-05 |
| LOC_Os05g47540 | CPuORF26 - conserved peptide uORF-containing transcript, expressed | Down | 2503 | 6.94E-05 |
| LOC_Os06g43600 | LTPL129 - Protease inhibitor/seed storage/LTP family protein precursor, expressed | Down | 1657 | 3.42E-12 |
| LOC_Os02g54254 | saccharopine dehydrogenase, putative, expressed | Down | 3913 | 6.10E-12 |
| LOC_Os02g48320 | DNA binding protein, putative, expressed | Down | 2259 | 1.38E-11 |
| LOC_Os07g45300 | tim44-like domain containing protein | Down | 1062 | 6.10E-05 |
| LOC_Os08g10500 | hypothetical protein | Down | 276 | 4.34E-12 |
| LOC_Os02g57620 | Citrate transporter protein, putative, expressed | Down | 2615 | 0.000395 |
| LOC_Os02g54060 | chaperonin, putative, expressed | Down | 1112 | 3.49E-07 |
| LOC_Os09g25380 | kinesin motor protein-related, putative, expressed | Down | 1866 | 1.88E-09 |
| LOC_Os01g60860 | spotted leaf 11, putative, expressed | Down | 2494 | 4.61E-08 |
| LOC_Os09g06770 | RING finger protein, putative, expressed | Down | 716 | 0.000344 |
| LOC_Os12g04240 | peflin, putative, expressed | Down | 1927 | 5.53E-11 |
| LOC_Os03g16480 | zinc finger family protein, putative, expressed | Down | 1486 | 1.88E-10 |
| LOC_Os03g20700 | magnesium-chelatase, putative, expressed | Down | 5655 | 8.26E-12 |
| LOC_Os11g40590 | DUF1399 containing protein, putative, expressed | Down | 3972 | 5.47E-12 |
| LOC_Os02g58260 | metallo-beta-lactamase family protein, putative, expressed | Down | 798 | 2.00E-05 |
| LOC_Os04g55410 | FGGY family of carbohydrate kinases, putative, expressed | Down | 2283 | 4.86E-06 |
| LOC_Os01g49200 | microtubule associated protein, putative, expressed | Down | 2553 | 0 |
| LOC_Os02g45450 | dehydration-responsive element-binding protein, putative, expressed | Down | 1272 | 8.61E-06 |
| LOC_Os06g21400 | hypothetical protein | Down | 198 | 2.04E-08 |
| LOC_Os03g57310 | syntaxin, putative, expressed | Down | 1577 | 3.13E-05 |
| LOC_Os05g41760 | AP2 domain containing protein, expressed | Down | 985 | 1.95E-12 |
| LOC_Os07g48280 | expressed protein | Down | 2726 | 0 |
| LOC_Os07g12340 | NAC domain-containing protein 67, putative, expressed | Down | 1313 | 3.19E-08 |
| LOC_Os09g06740 | zinc finger, C3HC4 type domain containing protein, expressed | Down | 1258 | 4.84E-05 |
| LOC_Os03g04070 | no apical meristem protein, putative, expressed | Down | 1300 | 0.000174 |
| LOC_Os03g10880 | BTBN5 - Bric-a-Brac, Tramtrack, Broad Complex BTB domain with non-phototropic hypocotyl 3 NPH3 and coiled-coil domains, expressed | Down | 2348 | 0.000311 |
| LOC_Os12g34860 | phenylalanyl-tRNA synthetase, putative, expressed | Down | 2115 | 7.44E-05 |
| LOC_Os11g11370 | expressed protein | Down | 937 | 5.85E-08 |
| LOC_Os11g10750 | OsSCP48 - Putative Serine Carboxypeptidase homologue, expressed | Down | 1923 | 0 |
| LOC_Os08g29110 | thioredoxin, putative, expressed | Down | 876 | 3.22E-05 |
| LOC_Os03g43720 | transporter family protein, putative, expressed | Down | 2961 | 0.000549 |
| LOC_Os08g14580 | haloacid dehalogenase-like hydrolase domain-containing protein 1A, putative, expressed | Down | 1197 | 1.57E-05 |
| LOC_Os03g04100 | expressed protein | Down | 2133 | 1.39E-11 |
| LOC_Os12g06780 | expressed protein | Down | 1152 | 2.80E-05 |
| LOC_Os02g48360 | pyrophosphate--fructose 6-phosphate 1-phosphotransferase subunit alpha, putative, expressed | Down | 2403 | 5.50E-12 |
| LOC_Os03g17900 | hydrolase, alpha/beta fold family protein, putative, expressed | Down | 2237 | 4.95E-12 |
| LOC_Os08g30020 | membrane protein, putative, expressed | Down | 2139 | 0.000418 |
| LOC_Os07g22400 | POLA3 - Putative DNA polymerase alpha complex subunit, expressed | Down | 1941 | 2.83E-10 |
| LOC_Os03g05750 | heavy-metal-associated domain-containing protein, putative, expressed | Down | 1561 | 6.25E-12 |
| LOC_Os07g04500 | expressed protein | Down | 786 | 2.18E-06 |
| LOC_Os03g19720 | EF hand family protein, putative, expressed | Down | 522 | 5.13E-10 |
| LOC_Os08g18920 | protein kinase domain containing protein, expressed | Down | 3173 | 9.24E-08 |
| LOC_Os09g04160 | expressed protein | Down | 2681 | 0 |
| LOC_Os12g02200 | CAMK_KIN1/SNF1/Nim1_like.6 - CAMK includes calcium/calmodulin depedent protein kinases, expressed | Down | 2193 | 1.47E-11 |
| LOC_Os12g07820 | OsAPx6 - Stromal Ascorbate Peroxidase encoding gene 5,8, expressed | Down | 1315 | 3.94E-07 |
| LOC_Os05g05480 | OsDegp6 - Putative Deg protease homologue, expressed | Down | 2325 | 0.00074 |
| LOC_Os03g58830 | BEE 1, putative, expressed | Down | 1282 | 8.08E-08 |
| LOC_Os06g36490 | ethylene-responsive element-binding protein, putative, expressed | Down | 1162 | 1.61E-05 |
| LOC_Os01g09800 | BTBA1 - Bric-a-Brac,Tramtrack, Broad Complex BTB domain with Ankyrin repeat region, expressed | Down | 2330 | 3.46E-08 |
| LOC_Os09g26700 | choline/ethanolamine kinase, putative, expressed | Down | 1860 | 0 |
| LOC_Os03g05540 | tetratricopeptide repeat containing protein, putative, expressed | Down | 2176 | 0.000276 |
| LOC_Os08g37432 | MATE efflux family protein, putative, expressed | Down | 2068 | 0.000118 |
| LOC_Os02g39850 | transferase family protein, putative, expressed | Down | 1865 | 0.00056 |
| LOC_Os02g50990 | RING-H2 finger protein ATL1Q, putative, expressed | Down | 1888 | 0.000559 |
| LOC_Os08g19210 | expressed protein | Down | 1518 | 0.000102 |
| LOC_Os04g02670 | hypothetical protein | Down | 159 | 7.21E-09 |
| LOC_Os12g06870 | OsNucAP2 - Putative Nucleoporin Autopeptidase homologue, expressed | Down | 3466 | 2.20E-10 |
| LOC_Os08g19670 | expressed protein | Down | 1015 | 2.21E-11 |
| LOC_Os01g40650 | expressed protein | Down | 527 | 3.44E-06 |
| LOC_Os04g52370 | UTP--glucose-1-phosphate uridylyltransferase, putative, expressed | Down | 2073 | 0.000424 |
| LOC_Os05g50910 | extra-large G-protein-related, putative, expressed | Down | 3119 | 1.27E-07 |
| LOC_Os04g45610 | expressed protein | Down | 1872 | 0.000182 |
| LOC_Os11g33100 | SNARE associated Golgi protein, putative, expressed | Down | 1332 | 3.49E-10 |
| LOC_Os05g15510 | cellulase, putative, expressed | Down | 1980 | 1.45E-10 |
| LOC_Os12g34018 | ATP synthase protein YMF19, putative, expressed | Down | 1161 | 0.000862 |
| LOC_Os05g30860 | expressed protein | Down | 2471 | 0.000862 |
| LOC_Os04g08350 | cysteine synthase, chloroplast/chromoplast precursor, putative, expressed | Down | 1624 | 1.64E-11 |
| LOC_Os02g47310 | Cyclopropane-fatty-acyl-phospholipid synthase, putative, expressed | Down | 1535 | 9.57E-08 |
| LOC_Os07g25810 | retrotransposon protein, putative, unclassified, expressed | Down | 1684 | 0.00037 |
| LOC_Os03g22270 | auxin-repressed protein, putative, expressed | Down | 2330 | 0 |
| LOC_Os06g45090 | expressed protein | Down | 2200 | 1.65E-11 |
| LOC_Os01g06590 | zinc finger, C3HC4 type domain containing protein, expressed | Down | 2875 | 2.25E-06 |
| LOC_Os03g17690 | OsAPx1 - Cytosolic Ascorbate Peroxidase encoding gene 1-8, expressed | Down | 1254 | 1.69E-10 |
| LOC_Os04g33820 | OsFBX132 - F-box domain containing protein, expressed | Down | 1299 | 6.09E-12 |
| LOC_Os02g16630 | tryptophan biosynthesis protein trpCF, putative, expressed | Down | 1924 | 0.000119 |
| LOC_Os09g23300 | integral membrane protein, putative, expressed | Down | 1230 | 0 |
| LOC_Os07g14150 | cytidine deaminase, putative, expressed | Down | 2180 | 8.24E-12 |
| LOC_Os07g43170 | adenylate kinase, putative, expressed | Down | 1829 | 9.67E-08 |
| LOC_Os03g10240 | DUF677 domain containing protein, putative, expressed | Down | 2257 | 8.66E-11 |
| LOC_Os08g27824 | NOL1/NOP2/sun family protein, putative, expressed | Down | 2295 | 1.12E-06 |
| LOC_Os03g44380 | 9-cis-epoxycarotenoid dioxygenase 1, chloroplast precursor, putative, expressed | Down | 2671 | 9.67E-11 |
| LOC_Os02g41510 | MYB family transcription factor, putative, expressed | Down | 1042 | 3.11E-07 |
| LOC_Os01g22954 | serine carboxypeptidase, putative, expressed | Down | 1984 | 1.51E-11 |
| LOC_Os04g40630 | BTBZ4 - Bric-a-Brac, Tramtrack, Broad Complex BTB domain with TAZ zinc finger and Calmodulin-binding domains, expressed | Down | 1714 | 8.27E-12 |
| LOC_Os10g21418 | NADPH-dependent oxidoreductase, putative | Down | 2205 | 0 |
| LOC_Os10g21190 | expressed protein | Down | 858 | 0 |
| LOC_Os05g45740 | mitochondrial ATP synthase g subunit family protein, putative, expressed | Down | 819 | 5.57E-08 |
| LOC_Os03g15560 | wibg, putative, expressed | Down | 1443 | 3.58E-07 |
| LOC_Os06g08440 | two-component response regulator, putative, expressed | Down | 3326 | 8.50E-07 |
| LOC_Os03g07380 | expressed protein | Down | 778 | 1.78E-09 |
| LOC_Os09g37920 | helicase, putative, expressed | Down | 4101 | 0.000213 |
| LOC_Os05g45460 | POEI52 - Pollen Ole e I allergen and extensin family protein precursor, expressed | Down | 1079 | 0 |
| LOC_Os04g46280 | hydrolase, NUDIX family, domain containing protein, expressed | Down | 1811 | 4.73E-12 |
| LOC_Os07g32680 | retrotransposon protein, putative, unclassified, expressed | Down | 1424 | 6.30E-12 |
| LOC_Os09g28520 | expressed protein | Down | 1467 | 1.53E-11 |
| LOC_Os06g46284 | glycosyl hydrolase, family 31, putative, expressed | Down | 3459 | 3.89E-12 |
| LOC_Os05g31620 | OsCML15 - Calmodulin-related calcium sensor protein, expressed | Down | 979 | 7.37E-11 |
| LOC_Os11g35400 | AMP-binding enzyme, putative, expressed | Down | 2532 | 2.17E-12 |
| LOC_Os02g47120 | region found in RelA/SpoT proteins containing protein, expressed | Down | 2985 | 1.16E-08 |
| LOC_Os09g38330 | MORN repeat domain containing protein, expressed | Down | 2873 | 0.000323 |
| LOC_Os10g41710 | chaperonin, putative, expressed | Down | 904 | 0.000323 |
| LOC_Os12g01770 | protein phosphatase 2c, putative, expressed | Down | 1729 | 1.26E-05 |
| LOC_Os04g56590 | ATP/GTP binding protein, putative, expressed | Down | 1595 | 5.57E-08 |
| LOC_Os11g40500 | OsSigP7 - Putative Type I Signal Peptidase homologue; employs a putative Ser/Lys catalytic dyad, expressed | Down | 1057 | 1.78E-08 |
| LOC_Os01g70790 | SRC2 protein, putative, expressed | Down | 1528 | 0 |
| LOC_Os03g45280 | dehydrin, putative, expressed | Down | 925 | 8.69E-12 |
| LOC_Os07g39980 | GA22009-PA, putative, expressed | Down | 1535 | 8.18E-06 |
| LOC_Os06g14406 | SYD, putative, expressed | Down | 2770 | 0 |
| LOC_Os02g41904 | DEF7 - Defensin and Defensin-like DEFL family | Down | 638 | 0 |
| LOC_Os07g09060 | aldehyde dehydrogenase, putative, expressed | Down | 2033 | 1.67E-10 |
| LOC_Os07g37570 | expressed protein | Down | 652 | 0.000491 |
| LOC_Os06g03670 | dehydration-responsive element-binding protein, putative, expressed | Down | 1127 | 1.65E-05 |
| LOC_Os01g52500 | NADP-dependent malic enzyme, putative, expressed | Down | 3179 | 4.85E-12 |
| LOC_Os01g59060 | 50S ribosomal protein, putative, expressed | Down | 756 | 0.000371 |
| LOC_Os05g08420 | expressed protein | Down | 845 | 0 |
| LOC_Os01g53710 | dual specificity protein phosphatase, putative, expressed | Down | 1591 | 1.11E-11 |
| LOC_Os01g05630 | Core histone H2A/H2B/H3/H4 domain containing protein, putative, expressed | Down | 972 | 1.11E-11 |
| LOC_Os05g41780 | AP2 domain containing protein, expressed | Down | 1130 | 0 |
| LOC_Os04g13260 | expressed protein | Down | 931 | 7.76E-05 |
| LOC_Os11g02330 | LTPL22 - Protease inhibitor/seed storage/LTP family protein precursor, expressed | Down | 770 | 2.07E-12 |
| LOC_Os04g49370 | expressed protein | Down | 1437 | 4.08E-11 |
| LOC_Os12g03540 | WD domain, G-beta repeat domain containing protein, expressed | Down | 1790 | 6.10E-06 |
| LOC_Os03g44810 | expressed protein | Down | 1661 | 6.75E-05 |
| LOC_Os09g28440 | AP2 domain containing protein, expressed | Down | 1100 | 5.31E-06 |
| LOC_Os06g22960 | aquaporin protein, putative, expressed | Down | 1358 | 0 |
| LOC_Os02g55910 | monogalactosyldiacylglycerol synthase, putative, expressed | Down | 2192 | 0 |
| LOC_Os01g17050 | VQ domain containing protein, putative, expressed | Down | 1023 | 0 |
| LOC_Os03g47980 | expressed protein | Down | 2270 | 0.000561 |
| LOC_Os08g32600 | STE_MEKK_ste11_MAP3K.21 - STE kinases include homologs to sterile 7, sterile 11 and sterile 20 from yeast, expressed | Down | 2718 | 5.47E-08 |
| LOC_Os02g17280 | gamma-secretase subunit APH-1B, putative, expressed | Down | 1127 | 2.68E-09 |
| LOC_Os01g16170 | PQ loop repeat domain containing protein, expressed | Down | 2352 | 0 |
| LOC_Os08g44850 | C2 domain containing protein, putative, expressed | Down | 1973 | 0.000422 |
| LOC_Os07g07930 | LTPL78 - Protease inhibitor/seed storage/LTP family protein precursor, expressed | Down | 974 | 4.67E-07 |
| LOC_Os03g15750 | ASC1, putative, expressed | Down | 1855 | 0.000102 |
| LOC_Os10g20450 | MATE efflux family protein, putative, expressed | Down | 1781 | 2.51E-05 |
| LOC_Os02g04520 | AGG2, putative, expressed | Down | 2537 | 6.05E-06 |
| LOC_Os08g37700 | RNA recognition motif containing protein, putative, expressed | Down | 3695 | 0.000984 |
| LOC_Os07g08500 | C-5 cytosine-specific DNA methylase, putative, expressed | Down | 1605 | 9.18E-06 |
| LOC_Os06g14420 | hydrolase, NUDIX family, domain containing protein, expressed | Down | 1454 | 1.38E-11 |
| LOC_Os03g40540 | cytochrome P450, putative, expressed | Down | 2005 | 0 |
| LOC_Os02g47670 | bg55, putative, expressed | Down | 3349 | 0.000135 |
| LOC_Os07g25002 | chloroplast 30S ribosomal protein S7, putative, expressed | Down | 586 | 5.96E-06 |
| LOC_Os09g27140 | expressed protein | Down | 1351 | 0.000479 |
| LOC_Os06g04800 | peptidase, T1 family, putative, expressed | Down | 1129 | 0 |
| LOC_Os06g04230 | expressed protein | Down | 517 | 6.91E-12 |
| LOC_Os02g03220 | protein binding protein, putative, expressed | Down | 2237 | 7.55E-05 |
| LOC_Os01g34870 | expressed protein | Down | 1420 | 1.94E-08 |
| LOC_Os07g14350 | methyltransferase, putative, expressed | Down | 1392 | 1.21E-05 |
| LOC_Os09g39440 | inosine-uridine preferring nucleoside hydrolase family protein, putative, expressed | Down | 1533 | 1.80E-11 |
| LOC_Os01g15290 | ribosomal L18p/L5e family protein, putative, expressed | Down | 770 | 1.04E-05 |
| LOC_Os01g52110 | RING finger and CHY zinc finger domain-containing protein 1, putative, expressed | Down | 3847 | 0 |
| LOC_Os10g35840 | shikimate/quinate 5-dehydrogenase, putative, expressed | Down | 2319 | 1.06E-11 |
| LOC_Os03g11500 | DUF647 domain containing protein, putative, expressed | Down | 1410 | 4.29E-05 |
| LOC_Os03g22790 | beta-amylase, putative, expressed | Down | 2022 | 3.58E-11 |
| LOC_Os03g58040 | glutamate dehydrogenase protein, putative, expressed | Down | 1698 | 0 |
| LOC_Os03g15540 | HEAT repeat family protein, putative, expressed | Down | 2243 | 0.000837 |
| LOC_Os03g39000 | inositol-1-monophosphatase, putative, expressed | Down | 1232 | 9.88E-05 |
| LOC_Os09g26160 | glutamate receptor, putative, expressed | Down | 3437 | 2.14E-06 |
| LOC_Os05g29010 | POLA4 - Putative DNA polymerase alpha complex subunit, expressed | Down | 2955 | 2.89E-08 |
| LOC_Os01g74370 | domain of unknown function DUF966 domain containing protein, expressed | Down | 1501 | 1.67E-11 |
| LOC_Os01g05970 | OsFBO1 - F-box and other domain containing protein, expressed | Down | 767 | 2.07E-12 |
| LOC_Os11g05730 | histone H3, putative, expressed | Down | 622 | 0.00047 |
| LOC_Os07g36170 | chitin-inducible gibberellin-responsive protein, putative, expressed | Down | 2906 | 0 |
| LOC_Os05g33140 | CHIT5 - Chitinase family protein precursor, expressed | Up | 1287 | 6.65E-14 |
| LOC_Os03g52860 | lipoxygenase, putative, expressed | Up | 2941 | 1.61E-35 |
| LOC_Os03g14180 | hsp20/alpha crystallin family protein, putative, expressed | Up | 1270 | 6.47E-19 |
| LOC_Os03g53610 | late embryogenesis abundant protein D-34, putative | Up | 636 | 1.82E-22 |
| LOC_Os09g33680 | Os9bglu31 - beta-glucosidase, dhurrinase, similar to G. max hydroxyisourate hydrolase, expressed | Up | 2337 | ######## |
| LOC_Os06g26180 | hypothetical protein | Up | 468 | 6.43E-07 |
| LOC_Os02g09250 | cytochrome P450 71D10, putative, expressed | Up | 1818 | 2.96E-23 |
| LOC_Os02g04780 | expressed protein | Up | 1396 | 5.72E-23 |
| LOC_Os01g73720 | RCLEA4 - Root cap and Late embryogenesis related family protein precursor, expressed | Up | 1353 | 6.28E-08 |
| LOC_Os08g01370 | expressed protein | Up | 291 | 4.10E-97 |
| LOC_Os01g06630 | small hydrophilic plant seed protein, putative, expressed | Up | 863 | ######## |
| LOC_Os05g04870 | oxidoreductase, short chain dehydrogenase/reductase family protein, putative, expressed | Up | 1386 | 0 |
| LOC_Os04g02754 | amidase family protein, putative, expressed | Up | 1781 | 2.61E-10 |
| LOC_Os01g04660 | lipid phosphatase protein, putative, expressed | Up | 1703 | 2.62E-12 |
| LOC_Os01g43851 | cytochrome P450 72A1, putative, expressed | Up | 1760 | 5.29E-09 |
| LOC_Os01g63210 | SOUL heme-binding protein, putative, expressed | Up | 959 | 8.98E-25 |
| LOC_Os09g08280 | expressed protein | Up | 586 | ######## |
| LOC_Os08g30210 | 1-aminocyclopropane-1-carboxylate oxidase homolog 1, putative, expressed | Up | 1382 | 0.000822 |
| LOC_Os05g39690 | oxidoreductase, aldo/keto reductase family protein, putative, expressed | Up | 1297 | 0 |
| LOC_Os06g32355 | conserved hypothetical protein | Up | 237 | 3.83E-05 |
| LOC_Os02g48570 | peptide transporter PTR2, putative, expressed | Up | 2590 | 1.06E-14 |
| LOC_Os10g36180 | expressed protein | Up | 1650 | ######## |
| LOC_Os06g04940 | early nodulin 93 ENOD93 protein, putative, expressed | Up | 1045 | ######## |
| LOC_Os03g52380 | PIII5 - Proteinase inhibitor II family protein precursor, expressed | Up | 598 | 4.32E-07 |
| LOC_Os11g10590 | hypothetical protein | Up | 267 | ######## |
| LOC_Os01g64670 | soluble inorganic pyrophosphatase, putative, expressed | Up | 1641 | 1.32E-06 |
| LOC_Os12g37320 | lipoxygenase 2.2, chloroplast precursor, putative, expressed | Up | 1520 | 1.49E-25 |
| LOC_Os04g33990 | harpin-induced protein 1 domain containing protein, expressed | Up | 1098 | 6.57E-14 |
| LOC_Os09g20440 | succinate dehydrogenase and fumarate reductase iron-sulfur protein | Up | 1854 | 2.27E-06 |
| LOC_Os11g24070 | LTPL10 - Protease inhibitor/seed storage/LTP family protein precursor, expressed | Up | 1011 | 1.86E-15 |
| LOC_Os07g46360 | glyoxalase family protein, putative, expressed | Up | 900 | 1.13E-72 |
| LOC_Os05g30490 | harpin-induced protein 1 domain containing protein | Up | 783 | 2.68E-13 |
| LOC_Os04g41970 | endoglucanase, putative, expressed | Up | 2407 | 1.11E-10 |
| LOC_Os03g53620 | late embryogenesis abundant protein D-34, putative, expressed | Up | 775 | 3.56E-41 |
| LOC_Os01g55000 | expressed protein | Up | 1493 | 1.97E-05 |
| LOC_Os01g58670 | conserved hypothetical protein | Up | 1689 | 1.97E-05 |
| LOC_Os11g32890 | expressed protein | Up | 824 | 2.19E-07 |
| LOC_Os07g47840 | expressed protein | Up | 1573 | 1.09E-06 |
| LOC_Os02g52560 | xyloglucan fucosyltransferase, putative, expressed | Up | 2206 | 2.02E-08 |
| LOC_Os09g36700 | ribonuclease T2 family domain containing protein, expressed | Up | 1213 | 1.27E-46 |
| LOC_Os09g31430 | Os9bglu30 - beta-glucosidase, similar to Os4bglu12 exoglucanase, expressed | Up | 1844 | 2.58E-13 |
| LOC_Os04g20774 | pumilio-family RNA binding repeat containing protein, expressed | Up | 1806 | 0.000676 |
| LOC_Os10g30560 | UDP-glucoronosyl and UDP-glucosyl transferase domain containing protein, expressed | Up | 1921 | 0.000676 |
| LOC_Os02g04160 | transcription elongation factor 1, putative, expressed | Up | 923 | 1.83E-06 |
| LOC_Os01g57690 | early nodulin 20 precursor, putative, expressed | Up | 1180 | 0.000159 |
| LOC_Os02g06410 | CBS domain containing membrane protein, putative, expressed | Up | 1478 | 1.69E-19 |
| LOC_Os03g54050 | anther-specific proline-rich protein APG precursor, putative, expressed | Up | 1613 | 1.50E-14 |
| LOC_Os06g31890 | THION3 - Plant thionin family protein precursor, expressed | Up | 816 | ######## |
| LOC_Os11g32650 | chalcone synthase, putative, expressed | Up | 1805 | 9.56E-13 |
| LOC_Os12g05210 | expressed protein | Up | 823 | 2.21E-45 |
| LOC_Os11g26750 | dehydrin, putative, expressed | Up | 952 | 7.79E-07 |
| LOC_Os04g52750 | expressed protein | Up | 489 | ######## |
| LOC_Os09g04100 | expressed protein | Up | 742 | 4.28E-28 |
| LOC_Os05g49300 | iron-sulfur cluster assembly enzyme ISCU, mitochondrial precursor, putative, expressed | Up | 977 | 4.73E-16 |
| LOC_Os01g04370 | hsp20/alpha crystallin family protein, putative, expressed | Up | 851 | ######## |
| LOC_Os01g12580 | late embryogenesis abundant protein, putative, expressed | Up | 1255 | 5.93E-37 |
| LOC_Os07g20340 | heavy metal-associated domain containing protein, expressed | Up | 1386 | ######## |
| LOC_Os01g04380 | hsp20/alpha crystallin family protein, putative, expressed | Up | 844 | 1.80E-22 |
| LOC_Os04g01690 | pyridoxal-dependent decarboxylase protein, putative, expressed | Up | 2146 | 1.51E-05 |
| LOC_Os04g44210 | expressed protein | Up | 2222 | 1.40E-21 |
| LOC_Os05g31670 | AWPM-19-like membrane family protein, putative, expressed | Up | 970 | ######## |
| LOC_Os08g42720 | solute carrier family 35 member F1, putative, expressed | Up | 1668 | 4.02E-05 |
| LOC_Os06g23350 | late embryogenesis abundant protein D-34, putative, expressed | Up | 1121 | 1.31E-30 |
| LOC_Os08g06100 | O-methyltransferase, putative, expressed | Up | 1518 | 5.10E-07 |
| LOC_Os12g43140 | late embryogenesis abundant protein D-34, putative, expressed | Up | 675 | 4.47E-08 |
| LOC_Os01g04360 | hsp20/alpha crystallin family protein, putative, expressed | Up | 789 | 1.29E-10 |
| LOC_Os03g51350 | expressed protein | Up | 959 | 0 |
| LOC_Os04g01740 | heat shock protein, putative, expressed | Up | 2584 | 3.23E-12 |
| LOC_Os07g44430 | peroxiredoxin, putative | Up | 768 | 0 |
| LOC_Os02g39000 | remorin C-terminal domain containing protein, putative, expressed | Up | 1478 | 3.21E-10 |
| LOC_Os06g21910 | late embryogenesis abundant group 1, putative, expressed | Up | 553 | 0 |
| LOC_Os08g23170 | expressed protein | Up | 688 | ######## |
| LOC_Os07g24000 | AWPM-19-like membrane family protein, putative, expressed | Up | 1248 | 0 |
| LOC_Os03g03810 | DEF8 - Defensin and Defensin-like DEFL family, expressed | Up | 579 | 1.56E-53 |
| LOC_Os02g15250 | late embryogenesis abundant domain-containing protein, putative, expressed | Up | 1672 | 0 |
| LOC_Os03g27800 | paramyosin, putative, expressed | Up | 2897 | 2.35E-05 |
| LOC_Os03g02050 | LTPL151 - Protease inhibitor/seed storage/LTP family protein precursor, expressed | Up | 811 | 0 |
| LOC_Os05g31020 | eukaryotic peptide chain release factor subunit 1-1, putative, expressed | Up | 2132 | 2.30E-70 |
| LOC_Os01g45659 | hypothetical protein | Up | 582 | 3.34E-09 |
| LOC_Os11g26570 | dehydrin, putative, expressed | Up | 1405 | 0 |
| LOC_Os07g28480 | glutathione S-transferase, putative, expressed | Up | 1207 | 2.30E-26 |
| LOC_Os02g30600 | conserved hypothetical protein | Up | 210 | 6.63E-20 |
| LOC_Os02g07840 | bZIP transcription factor domain containing protein, expressed | Up | 1396 | 5.19E-15 |
| LOC_Os05g49730 | protein phosphatase 2C, putative, expressed | Up | 1497 | 9.08E-05 |
| LOC_Os02g54140 | hsp20/alpha crystallin family protein, putative, expressed | Up | 885 | 1.75E-15 |
| LOC_Os04g35490 | expressed protein | Up | 1395 | 2.17E-63 |
| LOC_Os07g49360 | peroxidase precursor, putative, expressed | Up | 1356 | 0.000628 |
| LOC_Os01g29780 | expressed protein | Up | 785 | 3.73E-12 |
| LOC_Os08g42910 | peptidase, M24 family protein, putative, expressed | Up | 1774 | 2.33E-11 |
| LOC_Os05g39250 | phosphatidylethanolamine-binding protein, putative, expressed | Up | 758 | 7.31E-79 |
| LOC_Os01g16920 | embryonic protein DC-8, putative, expressed | Up | 966 | 2.51E-57 |
| LOC_Os05g05930 | peripheral-type benzodiazepine receptor, putative, expressed | Up | 829 | 3.32E-11 |
| LOC_Os06g32240 | THION9 - Plant thionin family protein precursor, expressed | Up | 1004 | ######## |
| LOC_Os11g26760 | dehydrin, putative, expressed | Up | 795 | 3.16E-26 |
| LOC_Os01g42520 | expressed protein | Up | 1879 | 1.19E-15 |
| LOC_Os02g57110 | GDSL-like lipase/acylhydrolase, putative, expressed | Up | 1668 | 2.81E-08 |
| LOC_Os06g27760 | peptide methionine sulfoxide reductase msrB, putative, expressed | Up | 1029 | 3.36E-07 |
| LOC_Os04g32080 | 11-beta-hydroxysteroid dehydrogenase, putative, expressed | Up | 1449 | ######## |
| LOC_Os02g15740 | expressed protein | Up | 1242 | 1.20E-55 |
| LOC_Os07g05020 | expressed protein | Up | 543 | 0.000119 |
| LOC_Os03g04660 | cytochrome P450 86A1, putative, expressed | Up | 2135 | 9.34E-06 |
| LOC_Os08g45110 | AP2 domain containing protein, expressed | Up | 1179 | 1.54E-14 |
| LOC_Os04g59260 | peroxidase precursor, putative, expressed | Up | 1558 | 2.30E-28 |
| LOC_Os04g43360 | Os4bglu14 - monolignol beta-glucoside homologue without catalytic acid/base, expressed | Up | 2026 | 5.09E-54 |
| LOC_Os03g16940 | glyoxalase family protein, putative, expressed | Up | 870 | 0 |
| LOC_Os05g07560 | expressed protein | Up | 4291 | 1.43E-05 |
| LOC_Os01g45624 | oleosin, putative, expressed | Up | 700 | 4.97E-06 |
| LOC_Os08g23870 | late embryogenesis abundant group 1, putative, expressed | Up | 841 | 0 |
| LOC_Os11g02440 | chalcone--flavonone isomerase, putative, expressed | Up | 1208 | 1.64E-15 |
| LOC_Os04g39864 | Os4bglu11 - beta-glucosidase homologue, similar to Os4Bglu12 exoglucanase/beta-glucosidase, expressed | Up | 1954 | 0.000421 |
| LOC_Os01g03360 | BBTI5 - Bowman-Birk type bran trypsin inhibitor precursor, expressed | Up | 1174 | 0 |
| LOC_Os03g28990 | zinc finger family protein, putative, expressed | Up | 1933 | 5.55E-13 |
| LOC_Os06g30179 | cytochrome P450, putative, expressed | Up | 2108 | ######## |
| LOC_Os03g04190 | cytochrome P450, putative | Up | 1548 | 7.74E-17 |
| LOC_Os03g53900 | universal stress protein domain containing protein, putative, expressed | Up | 1566 | 2.38E-90 |
| LOC_Os06g11090 | CXE carboxylesterase, putative, expressed | Up | 1729 | 0.000632 |
| LOC_Os01g59020 | cytochrome P450, putative, expressed | Up | 1760 | 1.01E-05 |
| LOC_Os03g04220 | glutathione S-transferase, putative, expressed | Up | 1335 | 1.41E-10 |
| LOC_Os01g63010 | universal stress protein domain containing protein, putative, expressed | Up | 1697 | 3.58E-33 |
| LOC_Os12g43340 | actin-depolymerizing factor, putative, expressed | Up | 833 | 7.84E-25 |
| LOC_Os12g02340 | LTPL14 - Protease inhibitor/seed storage/LTP family protein precursor, expressed | Up | 903 | 3.57E-07 |
| LOC_Os04g38940 | integral membrane protein, putative, expressed | Up | 1344 | 0.000557 |
| LOC_Os04g49980 | late embryogenesis abundant group 1, putative, expressed | Up | 775 | 0 |
| LOC_Os11g02400 | LTPL8 - Protease inhibitor/seed storage/LTP family protein precursor, expressed | Up | 1135 | 9.30E-13 |
| LOC_Os08g40720 | FAD-binding and arabino-lactone oxidase domains containing protein, putative, expressed | Up | 1743 | 3.41E-05 |
| LOC_Os06g05480 | expressed protein | Up | 915 | 1.15E-11 |
| LOC_Os04g59150 | peroxidase precursor, putative, expressed | Up | 1717 | 6.87E-06 |
| LOC_Os06g32370 | THION16 - Plant thionin family protein precursor, putative | Up | 447 | 6.86E-06 |
| LOC_Os01g32380 | expressed protein | Up | 590 | 4.10E-07 |
| LOC_Os10g39610 | expressed protein | Up | 681 | 0 |
| LOC_Os02g49520 | armadillo/beta-catenin repeat family protein, putative, expressed | Up | 4094 | 0.000723 |
| LOC_Os02g01150 | erythronate-4-phosphate dehydrogenase domain containing protein, expressed | Up | 2997 | 0.000217 |
| LOC_Os04g40540 | protein-L-isoaspartate O-methyltransferase, putative, expressed | Up | 2188 | 1.56E-07 |
| LOC_Os12g31850 | ureide permease, putative, expressed | Up | 2301 | 0.000626 |
| LOC_Os05g47870 | expressed protein | Up | 3077 | 4.63E-09 |
| LOC_Os06g18670 | anthocyanidin 3-O-glucosyltransferase, putative, expressed | Up | 1795 | 7.80E-76 |
| LOC_Os02g26470 | expressed protein | Up | 544 | 6.58E-22 |
| LOC_Os03g31750 | pyruvate, phosphate dikinase, chloroplast precursor, putative, expressed | Up | 3097 | ######## |
| LOC_Os10g29570 | late embryogenesis abundant protein D-34, putative, expressed | Up | 486 | 0.00032 |
| LOC_Os06g33330 | powdery mildew resistant protein 5, putative, expressed | Up | 1961 | 4.15E-34 |
| LOC_Os09g33550 | CCT/B-box zinc finger protein, putative, expressed | Up | 1938 | 1.98E-06 |
| LOC_Os05g44340 | heat shock protein 101, putative, expressed | Up | 3264 | 1.20E-32 |
| LOC_Os05g46480 | late embryogenesis abundant protein, group 3, putative, expressed | Up | 1296 | 0 |
| LOC_Os07g38290 | plastocyanin-like domain containing protein, putative, expressed | Up | 992 | 8.64E-10 |
| LOC_Os01g65670 | amino acid transporter, putative, expressed | Up | 1798 | 9.19E-12 |
| LOC_Os11g07911 | expressed protein | Up | 701 | 2.51E-06 |
| LOC_Os04g35270 | alpha-1,4-fucosyltransferase, putative, expressed | Up | 1371 | 4.16E-05 |
| LOC_Os06g44190 | hypothetical protein | Up | 243 | 3.15E-22 |
| LOC_Os11g37270 | AMBP1 - Antimicrobial peptide MBP-1 family protein precursor, expressed | Up | 1727 | 0 |
| LOC_Os11g26790 | dehydrin, putative, expressed | Up | 1207 | 0 |
| LOC_Os11g07600 | ABC-2 type transporter domain containing protein, expressed | Up | 2195 | 1.85E-06 |
| LOC_Os05g38230 | oxidoreductase, aldo/keto reductase family protein, putative, expressed | Up | 1635 | 6.49E-13 |
| LOC_Os07g38130 | polygalacturonase inhibitor 1 precursor, putative, expressed | Up | 1456 | 2.32E-21 |
| LOC_Os12g36240 | inhibitor I family protein, putative | Up | 258 | 2.46E-32 |
| LOC_Os09g21919 | expressed protein | Up | 1109 | 1.28E-29 |
| LOC_Os07g10580 | PROLM26 - Prolamin precursor, expressed | Up | 694 | 7.47E-28 |
| LOC_Os01g41720 | conserved hypothetical protein | Up | 1728 | 2.36E-07 |
| LOC_Os03g20120 | glycosyl transferase 8 domain containing protein, putative, expressed | Up | 1433 | 6.84E-07 |
| LOC_Os03g06180 | expressed protein | Up | 2398 | 4.02E-66 |
| LOC_Os02g15930 | expressed protein | Up | 1592 | 1.02E-12 |
| LOC_Os04g38790 | expressed protein | Up | 1199 | 0.000496 |
| LOC_Os03g63280 | regulatory protein, putative, expressed | Up | 1604 | 7.45E-05 |
| LOC_Os01g47400 | OsMan01 - Endo-Beta-Mannanase, expressed | Up | 1776 | 2.50E-07 |
| LOC_Os12g36220 | inhibitor I family protein, putative, expressed | Up | 545 | 6.95E-55 |
| LOC_Os01g39020 | HSF-type DNA-binding domain containing protein, expressed | Up | 1569 | 6.19E-08 |
| LOC_Os06g24430 | hypothetical protein | Up | 624 | 1.05E-07 |
| LOC_Os09g19890 | expressed protein | Up | 2652 | 2.48E-14 |
| LOC_Os06g49190 | LTPL154 - Protease inhibitor/seed storage/LTP family protein precursor, expressed | Up | 650 | 8.67E-11 |
| LOC_Os03g04770 | beta-amylase, putative, expressed | Up | 2304 | 1.10E-66 |
| LOC_Os01g64120 | 2Fe-2S iron-sulfur cluster binding domain containing protein, expressed | Up | 949 | 1.16E-18 |
| LOC_Os04g40510 | glycosyl hydrolase family 5 protein, putative, expressed | Up | 2819 | 3.52E-18 |
| LOC_Os08g03410 | glutelin, putative, expressed | Up | 1871 | 0 |
| LOC_Os10g38090 | cytochrome P450, putative, expressed | Up | 2016 | 1.22E-21 |
| LOC_Os03g46100 | cupin domain containing protein, expressed | Up | 1965 | 0 |
| LOC_Os03g30530 | expressed protein | Up | 3312 | 3.62E-12 |
| LOC_Os11g02424 | LTPL9 - Protease inhibitor/seed storage/LTP family protein precursor, expressed | Up | 1249 | 1.09E-06 |
| LOC_Os06g04990 | early nodulin 93 ENOD93 protein, putative, expressed | Up | 1006 | 1.99E-62 |
| LOC_Os01g10580 | B-box zinc finger family protein, putative, expressed | Up | 1526 | 3.63E-06 |
| LOC_Os05g28210 | small hydrophilic plant seed protein, putative, expressed | Up | 816 | 0 |
| LOC_Os01g15830 | peroxidase precursor, putative, expressed | Up | 1501 | 0.000739 |
| LOC_Os12g03530 | alpha-N-arabinofuranosidase, putative, expressed | Up | 2098 | 4.78E-05 |
| LOC_Os05g40010 | LTPL17 - Protease inhibitor/seed storage/LTP family protein precursor, expressed | Up | 1294 | 9.40E-05 |
| LOC_Os05g35200 | glycosyl transferase, putative, expressed | Up | 2628 | 1.65E-18 |
| LOC_Os01g52830 | DUF1264 domain containing protein, putative, expressed | Up | 1449 | 2.29E-77 |
| LOC_Os04g39150 | pathogenesis-related Bet v I family protein, putative, expressed | Up | 885 | 3.54E-14 |
| LOC_Os09g26380 | aminotransferase, classes I and II, domain containing protein, expressed | Up | 1927 | 4.36E-76 |
| LOC_Os06g05010 | early nodulin 93 ENOD93 protein, putative, expressed | Up | 727 | 1.43E-21 |
| LOC_Os07g10570 | PROLM25 - Prolamin precursor, expressed | Up | 972 | 3.51E-18 |
| LOC_Os02g55080 | snf1-related kinase interactor 2, putative, expressed | Up | 1201 | 7.33E-12 |
| LOC_Os04g55730 | alpha-N-acetylglucosaminidase, putative, expressed | Up | 2892 | 3.05E-09 |
| LOC_Os01g54300 | OsMan02 - Endo-Beta-Mannanase | Up | 2178 | 1.55E-13 |
| LOC_Os11g02389 | protease inhibitor/seed storage/LTP family, putative, expressed | Up | 799 | 5.61E-96 |
| LOC_Os02g06560 | wound induced protein, putative, expressed | Up | 267 | 5.46E-12 |
| LOC_Os03g26490 | expressed protein | Up | 902 | 1.05E-08 |
| LOC_Os03g07180 | embryonic protein DC-8, putative, expressed | Up | 2079 | ######## |
| LOC_Os12g02370 | chalcone--flavonone isomerase, putative, expressed | Up | 1289 | 3.81E-11 |
| LOC_Os08g38270 | fasciclin domain containing protein, expressed | Up | 1252 | 3.37E-07 |
| LOC_Os11g18570 | cytochrome P450, putative, expressed | Up | 1819 | 6.81E-05 |
| LOC_Os05g08044 | expressed protein | Up | 1420 | 2.51E-15 |
| LOC_Os05g04820 | MYB family transcription factor, putative, expressed | Up | 3730 | 0.000136 |
| LOC_Os03g12730 | receptor protein kinase CLAVATA1 precursor, putative, expressed | Up | 3871 | 1.24E-05 |
| LOC_Os12g44270 | glycine-rich protein, putative | Up | 372 | 0.00053 |
| LOC_Os07g22224 | expressed protein | Up | 801 | 5.85E-57 |
| LOC_Os04g28820 | retrotransposon protein, putative, unclassified, expressed | Up | 6249 | ######## |
| LOC_Os08g34280 | cinnamoyl-CoA reductase, putative, expressed | Up | 1495 | 0.000446 |
| LOC_Os03g57960 | cupin domain containing protein, expressed | Up | 1800 | 0 |
| LOC_Os04g52110 | late embryogenesis abundant protein, group 3, putative, expressed | Up | 1074 | ######## |
| LOC_Os03g59460 | transcription factor, putative | Up | 990 | 1.67E-07 |
| LOC_Os12g36210 | inhibitor I family protein, putative, expressed | Up | 566 | 5.90E-08 |
| LOC_Os05g15770 | glycosyl hydrolase, putative, expressed | Up | 1315 | 0.000268 |
| LOC_Os11g12710 | retrotransposon protein, putative, unclassified | Up | 1707 | 0.000624 |
| LOC_Os01g46580 | actin-related protein 2/3 complex subunit 2, putative, expressed | Up | 2131 | 4.22E-85 |
| LOC_Os01g62290 | DnaK family protein, putative, expressed | Up | 2626 | 2.84E-24 |
| LOC_Os06g05020 | early nodulin 93 ENOD93 protein, putative, expressed | Up | 789 | 9.05E-42 |
| LOC_Os09g39730 | Core histone H2A/H2B/H3/H4 domain containing protein, putative, expressed | Up | 1585 | 1.47E-34 |
| LOC_Os01g56610 | homocysteine S-methyltransferase protein, putative, expressed | Up | 1439 | 4.41E-18 |
| LOC_Os01g50700 | dehydrin family protein, expressed | Up | 2213 | ######## |
| LOC_Os04g05050 | pectate lyase precursor, putative, expressed | Up | 2153 | 4.76E-05 |
| LOC_Os10g09930 | expressed protein | Up | 1012 | 0.000263 |
| LOC_Os02g05830 | ribulose bisphosphate carboxylase small chain, chloroplast precursor, putative, expressed | Up | 1359 | ######## |
| LOC_Os04g09390 | HEV3 - Hevein family protein precursor, expressed | Up | 1134 | 0 |
| LOC_Os06g02490 | acyl CoA binding protein, putative, expressed | Up | 528 | 1.86E-07 |
| LOC_Os03g15530 | expressed protein | Up | 2162 | 3.81E-54 |
| LOC_Os06g42754 | expressed protein | Up | 815 | 2.79E-09 |
| LOC_Os03g20680 | late embryogenesis abundant protein 1, putative, expressed | Up | 1325 | 0 |
| LOC_Os02g33380 | pectinesterase inhibitor domain containing protein, putative, expressed | Up | 859 | 5.07E-18 |
| LOC_Os02g25680 | expressed protein | Up | 1096 | 1.35E-05 |
| LOC_Os07g48460 | stress responsive protein, putative, expressed | Up | 1621 | 2.25E-24 |
| LOC_Os08g36320 | decarboxylase, putative, expressed | Up | 2042 | 3.65E-51 |
| LOC_Os05g43460 | DUF567 domain containing protein, putative, expressed | Up | 1013 | 5.88E-07 |
| LOC_Os03g28160 | jacalin-like lectin domain containing protein, expressed | Up | 952 | 1.05E-09 |
| LOC_Os01g18630 | aspartic proteinase oryzasin-1 precursor, putative, expressed | Up | 2142 | 2.01E-07 |
| LOC_Os01g03680 | BBTI8 - Bowman-Birk type bran trypsin inhibitor precursor, expressed | Up | 996 | ######## |
| LOC_Os06g23274 | zinc finger, C3HC4 type, domain containing protein, expressed | Up | 6165 | 9.46E-40 |
| LOC_Os04g54620 | expressed protein | Up | 249 | 2.56E-05 |
| LOC_Os01g56235 | expressed protein | Up | 1044 | 7.06E-05 |
| LOC_Os12g27830 | dehydrogenase/reductase, putative, expressed | Up | 1412 | ######## |
| LOC_Os06g48060 | ABC transporter, ATP-binding protein, putative, expressed | Up | 1543 | 3.49E-05 |
| LOC_Os04g43170 | caleosin related protein, putative, expressed | Up | 1236 | 0 |
| LOC_Os09g30240 | phosphofructokinase, putative, expressed | Up | 1588 | 0.000323 |
| LOC_Os02g04369 | expressed protein | Up | 805 | 3.34E-05 |
| LOC_Os05g25640 | cytochrome P450, putative, expressed | Up | 1839 | 1.46E-07 |
| LOC_Os03g57690 | aldehyde oxidase, putative | Up | 4068 | 1.26E-17 |
| LOC_Os03g11910 | DnaK family protein, putative, expressed | Up | 2387 | 1.22E-08 |
| LOC_Os04g18950 | expressed protein | Up | 491 | 0.000749 |
| LOC_Os05g04700 | OsRCI2-6 - Hydrophobic protein LTI6B, expressed | Up | 279 | 0.000214 |
| LOC_Os06g05000 | early nodulin 93 ENOD93 protein, putative, expressed | Up | 778 | 2.66E-10 |
| LOC_Os02g32860 | poly synthetase 3, putative, expressed | Up | 2918 | 0 |
| LOC_Os08g03690 | LTPL24 - Protease inhibitor/seed storage/LTP family protein precursor, expressed | Up | 856 | 7.58E-24 |
| LOC_Os12g02320 | LTPL12 - Protease inhibitor/seed storage/LTP family protein precursor, expressed | Up | 865 | ######## |
| LOC_Os08g37580 | homeobox associated leucine zipper, putative, expressed | Up | 1922 | 7.08E-06 |
| LOC_Os01g55690 | glutelin, putative, expressed | Up | 1737 | 4.68E-29 |
| LOC_Os06g32350 | THION12 - Plant thionin family protein precursor | Up | 399 | 8.68E-09 |
| LOC_Os02g41630 | phenylalanine ammonia-lyase, putative, expressed | Up | 2614 | 1.41E-34 |
| LOC_Os03g42520 | expressed protein | Up | 1934 | 1.39E-09 |
| LOC_Os03g12990 | phytosulfokines precursor, putative, expressed | Up | 838 | 3.52E-13 |
| LOC_Os01g68870 | leucine-rich repeat receptor protein kinase EXS precursor, putative, expressed | Up | 4667 | 6.24E-06 |
| LOC_Os03g07100 | LTPL82 - Protease inhibitor/seed storage/LTP family protein precursor, expressed | Up | 958 | 2.64E-37 |
| LOC_Os05g39320 | thiamine pyrophosphate enzyme, C-terminal TPP binding domain containing protein, expressed | Up | 1818 | 0.000103 |
| LOC_Os08g44750 | auxin-induced protein 5NG4, putative, expressed | Up | 1622 | 8.40E-06 |
| LOC_Os03g17790 | OsRCI2-5 - Putative low temperature and salt responsive protein, expressed | Up | 519 | 4.55E-10 |
| LOC_Os07g36610 | CSLF9 - cellulose synthase-like family F; beta1,3;1,4 glucan synthase, expressed | Up | 2841 | 1.35E-05 |
| LOC_Os06g04460 | hypersensitive-induced response protein, putative, expressed | Up | 1290 | 1.09E-06 |
| LOC_Os03g62590 | oxidoreductase, short chain dehydrogenase/reductase family domain containing protein, expressed | Up | 1512 | 1.12E-86 |
| LOC_Os04g14150 | dehydration response related protein, putative, expressed | Up | 1339 | 3.39E-07 |
| LOC_Os07g05370 | TKL_IRAK_CrRLK1L-1.15 - The CrRLK1L-1 subfamily has homology to the CrRLK1L homolog, expressed | Up | 3592 | 0.000129 |
| LOC_Os03g42430 | B3 DNA binding domain containing protein, expressed | Up | 1469 | 2.06E-08 |
| LOC_Os03g51010 | hydrolase, alpha/beta fold family domain containing protein, expressed | Up | 1864 | 0.000319 |
| LOC_Os03g21790 | cupin domain containing protein, expressed | Up | 2843 | ######## |
| LOC_Os09g32010 | ternary complex factor MIP1, putative, expressed | Up | 3027 | 4.73E-11 |
| LOC_Os01g13060 | CK1_CaseinKinase_1.1 - CK1 includes the casein kinase 1 kinases, expressed | Up | 3190 | 9.98E-05 |
| LOC_Os06g06980 | caffeoyl-CoA O-methyltransferase, putative, expressed | Up | 1151 | 3.68E-06 |
| LOC_Os01g37690 | sodium/calcium exchanger protein, putative, expressed | Up | 2098 | 1.84E-09 |
| LOC_Os10g38489 | glutathione S-transferase GSTU6, putative, expressed | Up | 1082 | 0.000147 |
| LOC_Os01g02160 | hydroxyproline-rich glycoprotein family protein, putative, expressed | Up | 821 | 2.91E-06 |
