# Supplementary material for: Transcriptomic Analysis of Rice (Oryza sativa) Developing Embryos Using the RNA-Seq Technique
Source: PLoS One. 2012 Feb 8;7(2):e30646. doi: 10.1371/journal.pone.0030646 (PMC3275597; doi:10.1371/journal.pone.0030646)
Supplement: Table S3 — Differentially expressed genes between R3 and R2. FDR: false discovery rate. We used FDR<0.001 and the absolute value of log2Ratio≥1 as the threshold to judge the significance of gene expression difference. (DOC) [file pone.0030646.s003.doc]

**Table S3** Differentially expressed genes between R3 and R2. FDR: false discovery rate. We used FDR < 0.001 and the absolute value of log_2_Ratio≥1 as the threshold to judge the significance of gene expression difference.

| ***GeneID*** | ***Description*** | ***Up-Down-***  ***Regulation***  ***(R3/R2)*** | ***Gene length***  ***(bp)*** | ***FDR*** |
| --- | --- | --- | --- | --- |
| LOC_Os01g59200 | DUF617 domain containing protein, expressed | Up | 1225 | 4.84E-05 |
| LOC_Os03g16920 | DnaK family protein, putative, expressed | Up | 2485 | 4.60E-06 |
| LOC_Os12g13445 | expressed protein | Up | 1075 | 0.000616 |
| LOC_Os06g49880 | B-box zinc finger family protein, putative, expressed | Up | 1422 | 0.000535 |
| LOC_Os01g55160 | expressed protein | Up | 976 | 1.42E-06 |
| LOC_Os03g42464 | expressed protein | Up | 4373 | 4.60E-06 |
| LOC_Os08g01370 | expressed protein | Up | 291 | 4.35E-11 |
| LOC_Os12g44190 | ATPase 3, putative, expressed | Up | 2073 | 0.000104 |
| LOC_Os03g21260 | 2,3-bisphosphoglycerate-independent phosphoglycerate mutase, putative | Up | 1677 | 8.74E-05 |
| LOC_Os02g09250 | cytochrome P450 71D10, putative, expressed | Up | 1818 | 2.83E-12 |
| LOC_Os05g44810 | OsIAA18 - Auxin-responsive Aux/IAA gene family member, expressed | Up | 1396 | 0.000965 |
| LOC_Os05g33140 | CHIT5 - Chitinase family protein precursor, expressed | Up | 1287 | 2.40E-12 |
| LOC_Os09g33876 | expressed protein | Up | 2322 | 3.61E-07 |
| LOC_Os07g28850 | retrotransposon protein, putative, unclassified, expressed | Up | 3538 | 0.000263 |
| LOC_Os05g07560 | expressed protein | Up | 4291 | 3.52E-12 |
| LOC_Os01g06630 | small hydrophilic plant seed protein, putative, expressed | Up | 863 | 0 |
| LOC_Os02g46610 | zinc-binding protein, putative, expressed | Up | 1371 | 0.000931 |
| LOC_Os06g02000 | adenylate kinase, putative, expressed | Up | 1267 | 2.85E-05 |
| LOC_Os02g04160 | transcription elongation factor 1, putative, expressed | Up | 923 | 6.08E-12 |
| LOC_Os03g03810 | DEF8 - Defensin and Defensin-like DEFL family, expressed | Up | 579 | 7.38E-11 |
| LOC_Os05g39690 | oxidoreductase, aldo/keto reductase family protein, putative, expressed | Up | 1297 | 0 |
| LOC_Os05g31670 | AWPM-19-like membrane family protein, putative, expressed | Up | 970 | 4.99E-12 |
| LOC_Os04g02754 | amidase family protein, putative, expressed | Up | 1781 | 2.62E-12 |
| LOC_Os02g15930 | expressed protein | Up | 1592 | 3.25E-11 |
| LOC_Os05g11510 | B-box zinc finger family protein, putative, expressed | Up | 2058 | 0.00084 |
| LOC_Os11g32890 | expressed protein | Up | 824 | 4.74E-12 |
| LOC_Os05g50710 | late embryogenesis abundant protein, putative, expressed | Up | 1222 | 6.72E-12 |
| LOC_Os07g47840 | expressed protein | Up | 1573 | 7.48E-12 |
| LOC_Os06g08250 | zinc finger family protein, putative, expressed | Up | 970 | 0.000733 |
| LOC_Os04g59520 | anthranilate phosphoribosyltransferase, putative, expressed | Up | 4001 | 3.45E-06 |
| LOC_Os03g27280 | CAMK_CAMK_like.19 - CAMK includes calcium/calmodulin depedent protein kinases, expressed | Up | 1720 | 2.70E-12 |
| LOC_Os01g04370 | hsp20/alpha crystallin family protein, putative, expressed | Up | 851 | 9.30E-11 |
| LOC_Os10g30560 | UDP-glucoronosyl and UDP-glucosyl transferase domain containing protein, expressed | Up | 1921 | 1.36E-06 |
| LOC_Os01g63210 | SOUL heme-binding protein, putative, expressed | Up | 959 | 6.98E-12 |
| LOC_Os02g33070 | expressed protein | Up | 806 | 0 |
| LOC_Os09g33680 | Os9bglu31 - beta-glucosidase, dhurrinase, similar to G. max hydroxyisourate hydrolase, expressed | Up | 2337 | 6.88E-11 |
| LOC_Os09g20400 | expressed protein | Up | 1441 | 4.30E-12 |
| LOC_Os01g55430 | OsFBT2 - F-box and tubby domain containing protein, expressed | Up | 1736 | 2.82E-07 |
| LOC_Os01g04660 | lipid phosphatase protein, putative, expressed | Up | 1703 | 2.66E-12 |
| LOC_Os07g16970 | rab GDP dissociation inhibitor alpha, putative, expressed | Up | 2045 | 0.000389 |
| LOC_Os03g52380 | PIII5 - Proteinase inhibitor II family protein precursor, expressed | Up | 598 | 1.56E-09 |
| LOC_Os12g37320 | lipoxygenase 2.2, chloroplast precursor, putative, expressed | Up | 1520 | 8.76E-13 |
| LOC_Os01g64670 | soluble inorganic pyrophosphatase, putative, expressed | Up | 1641 | 8.58E-09 |
| LOC_Os05g35360 | beta-galactosidase 7 precursor, putative, expressed | Up | 2543 | 3.23E-08 |
| LOC_Os01g58670 | conserved hypothetical protein | Up | 1689 | 1.96E-07 |
| LOC_Os10g36170 | LTPL160 - Protease inhibitor/seed storage/LTP family protein precursor, expressed | Up | 594 | 3.10E-05 |
| LOC_Os02g04780 | expressed protein | Up | 1396 | 2.13E-12 |
| LOC_Os11g05410 | Ser/Thr protein phosphatase family protein, putative, expressed | Up | 1607 | 7.21E-05 |
| LOC_Os03g17470 | IN2-1 protein, putative, expressed | Up | 1379 | 6.04E-07 |
| LOC_Os07g42910 | cytochrome c oxidase subunit, putative, expressed | Up | 902 | 7.47E-12 |
| LOC_Os03g26870 | WD-40 repeat family protein, putative, expressed | Up | 3358 | 1.27E-12 |
| LOC_Os10g36180 | expressed protein | Up | 1650 | 4.02E-11 |
| LOC_Os03g58580 | nodulin, putative, expressed | Up | 2331 | 2.84E-08 |
| LOC_Os04g47590 | niemann-Pick C1 protein precursor, putative, expressed | Up | 3936 | 9.60E-11 |
| LOC_Os04g33990 | harpin-induced protein 1 domain containing protein, expressed | Up | 1098 | 8.46E-12 |
| LOC_Os02g48570 | peptide transporter PTR2, putative, expressed | Up | 2590 | 4.05E-12 |
| LOC_Os12g43140 | late embryogenesis abundant protein D-34, putative, expressed | Up | 675 | 0 |
| LOC_Os05g20450 | retrotransposon protein, putative, unclassified | Up | 4269 | 2.62E-12 |
| LOC_Os03g27800 | paramyosin, putative, expressed | Up | 2897 | 1.84E-08 |
| LOC_Os01g45659 | hypothetical protein | Up | 582 | 8.45E-12 |
| LOC_Os01g04360 | hsp20/alpha crystallin family protein, putative, expressed | Up | 789 | 0 |
| LOC_Os11g24070 | LTPL10 - Protease inhibitor/seed storage/LTP family protein precursor, expressed | Up | 1011 | 0 |
| LOC_Os03g11490 | expressed protein | Up | 3681 | 0 |
| LOC_Os03g14180 | hsp20/alpha crystallin family protein, putative, expressed | Up | 1270 | 2.42E-12 |
| LOC_Os09g20440 | succinate dehydrogenase and fumarate reductase iron-sulfur protein | Up | 1854 | 6.37E-06 |
| LOC_Os01g03360 | BBTI5 - Bowman-Birk type bran trypsin inhibitor precursor, expressed | Up | 1174 | 0 |
| LOC_Os01g04380 | hsp20/alpha crystallin family protein, putative, expressed | Up | 844 | 2.06E-11 |
| LOC_Os11g03700 | histone-lysine N-methyltransferase, H3 lysine-9 specific SUVH1, putative | Up | 1902 | 2.79E-06 |
| LOC_Os05g04870 | oxidoreductase, short chain dehydrogenase/reductase family protein, putative, expressed | Up | 1386 | 7.10E-10 |
| LOC_Os03g21650 | expressed protein | Up | 465 | 0.000588 |
| LOC_Os05g29050 | phospholipase D p1, putative, expressed | Up | 4155 | 0.000287 |
| LOC_Os09g08280 | expressed protein | Up | 586 | 1.48E-10 |
| LOC_Os11g26750 | dehydrin, putative, expressed | Up | 952 | 8.12E-07 |
| LOC_Os05g47870 | expressed protein | Up | 3077 | 7.42E-12 |
| LOC_Os11g10590 | hypothetical protein | Up | 267 | 0 |
| LOC_Os05g35980 | expressed protein | Up | 4631 | 0.000869 |
| LOC_Os08g18974 | expressed protein | Up | 1891 | 2.95E-12 |
| LOC_Os03g52860 | lipoxygenase, putative, expressed | Up | 2941 | 0 |
| LOC_Os01g50616 | phosphatidylinositol transfer, putative, expressed | Up | 2817 | 7.25E-06 |
| LOC_Os12g07280 | ZOS12-02 - C2H2 zinc finger protein, expressed | Up | 2196 | 0.00058 |
| LOC_Os11g18570 | cytochrome P450, putative, expressed | Up | 1819 | 1.71E-09 |
| LOC_Os05g11090 | thioredoxin, putative, expressed | Up | 1503 | 0.00091 |
| LOC_Os03g54050 | anther-specific proline-rich protein APG precursor, putative, expressed | Up | 1613 | 0 |
| LOC_Os01g24070 | GATA zinc finger domain containing protein, expressed | Up | 1281 | 0.000119 |
| LOC_Os06g14406 | SYD, putative, expressed | Up | 2770 | 0 |
| LOC_Os12g31850 | ureide permease, putative, expressed | Up | 2301 | 1.37E-05 |
| LOC_Os03g31750 | pyruvate, phosphate dikinase, chloroplast precursor, putative, expressed | Up | 3097 | 2.66E-10 |
| LOC_Os03g18960 | calmodulin binding protein, putative, expressed | Up | 1527 | 0.000164 |
| LOC_Os09g19890 | expressed protein | Up | 2652 | 1.46E-11 |
| LOC_Os02g25680 | expressed protein | Up | 1096 | 5.19E-12 |
| LOC_Os05g20460 | retrotransposon protein, putative, unclassified | Up | 2235 | 2.53E-06 |
| LOC_Os01g18630 | aspartic proteinase oryzasin-1 precursor, putative, expressed | Up | 2142 | 3.21E-12 |
| LOC_Os05g05930 | peripheral-type benzodiazepine receptor, putative, expressed | Up | 829 | 6.94E-12 |
| LOC_Os06g25500 | hypothetical protein | Up | 1894 | 6.12E-05 |
| LOC_Os04g28250 | cysteine proteinase inhibitor precursor, putative, expressed | Up | 842 | 1.40E-07 |
| LOC_Os06g49250 | peptide transporter PTR2, putative, expressed | Up | 2595 | 1.26E-05 |
| LOC_Os07g39920 | formin, putative, expressed | Up | 2823 | 9.53E-05 |
| LOC_Os01g41720 | conserved hypothetical protein | Up | 1728 | 1.30E-10 |
| LOC_Os01g12580 | late embryogenesis abundant protein, putative, expressed | Up | 1255 | 5.27E-12 |
| LOC_Os03g17790 | OsRCI2-5 - Putative low temperature and salt responsive protein, expressed | Up | 519 | 3.38E-11 |
| LOC_Os01g11460 | zinc finger, C3HC4 type domain containing protein, expressed | Up | 1465 | 0.000149 |
| LOC_Os07g38290 | plastocyanin-like domain containing protein, putative, expressed | Up | 992 | 7.31E-12 |
| LOC_Os04g46390 | chaperone protein dnaJ, putative, expressed | Up | 1695 | 0 |
| LOC_Os03g04680 | cytochrome P450, putative, expressed | Up | 2104 | 0.00023 |
| LOC_Os06g43960 | expressed protein | Up | 1566 | 1.50E-06 |
| LOC_Os03g30530 | expressed protein | Up | 3312 | 7.35E-13 |
| LOC_Os12g04260 | astaxanthin synthase KC28, putative | Up | 1676 | 8.39E-05 |
| LOC_Os08g42720 | solute carrier family 35 member F1, putative, expressed | Up | 1668 | 0.000844 |
| LOC_Os04g28820 | retrotransposon protein, putative, unclassified, expressed | Up | 6249 | 6.65E-10 |
| LOC_Os02g07840 | bZIP transcription factor domain containing protein, expressed | Up | 1396 | 1.75E-11 |
| LOC_Os07g46990 | copper/zinc superoxide dismutase, putative, expressed | Up | 835 | 1.77E-11 |
| LOC_Os06g30130 | cysteine-rich receptor-like protein kinase 10 precursor, putative, expressed | Up | 1945 | 0.000728 |
| LOC_Os04g43360 | Os4bglu14 - monolignol beta-glucoside homologue without catalytic acid/base, expressed | Up | 2026 | 0 |
| LOC_Os03g62650 | ion channel DMI1-like, chloroplast precursor, putative, expressed | Up | 3392 | 7.21E-06 |
| LOC_Os02g06725 | expressed protein | Up | 2712 | 0.000962 |
| LOC_Os02g39000 | remorin C-terminal domain containing protein, putative, expressed | Up | 1478 | 2.61E-07 |
| LOC_Os06g23350 | late embryogenesis abundant protein D-34, putative, expressed | Up | 1121 | 2.14E-11 |
| LOC_Os03g18264 | expressed protein | Up | 4154 | 1.21E-08 |
| LOC_Os02g01150 | erythronate-4-phosphate dehydrogenase domain containing protein, expressed | Up | 2997 | 0.000148 |
| LOC_Os05g49300 | iron-sulfur cluster assembly enzyme ISCU, mitochondrial precursor, putative, expressed | Up | 977 | 6.65E-10 |
| LOC_Os07g09970 | LTPL84 - Protease inhibitor/seed storage/LTP family protein precursor, expressed | Up | 1007 | 0.000347 |
| LOC_Os06g48500 | expressed protein | Up | 2104 | 4.37E-08 |
| LOC_Os09g26380 | aminotransferase, classes I and II, domain containing protein, expressed | Up | 1927 | 1.47E-10 |
| LOC_Os03g04660 | cytochrome P450 86A1, putative, expressed | Up | 2135 | 8.11E-05 |
| LOC_Os08g42910 | peptidase, M24 family protein, putative, expressed | Up | 1774 | 1.16E-08 |
| LOC_Os01g10580 | B-box zinc finger family protein, putative, expressed | Up | 1526 | 2.82E-07 |
| LOC_Os12g05210 | expressed protein | Up | 823 | 3.36E-11 |
| LOC_Os05g45730 | ubiquinone oxidoreductase, putative, expressed | Up | 1150 | 2.46E-05 |
| LOC_Os06g50230 | expressed protein | Down | 1189 | 8.61E-11 |
| LOC_Os01g65590 | galactosyltransferase, putative, expressed | Down | 1764 | 1.30E-07 |
| LOC_Os01g08380 | transferase family protein, putative, expressed | Down | 1748 | 9.68E-07 |
| LOC_Os03g16260 | protein kinase, putative, expressed | Down | 1909 | 9.97E-09 |
| LOC_Os02g56680 | dehydrogenase, putative, expressed | Down | 1391 | 2.40E-23 |
| LOC_Os01g54340 | plant-specific domain TIGR01615 family protein, expressed | Down | 1323 | 1.19E-14 |
| LOC_Os12g24020 | rhodanese-like domain containing protein, putative, expressed | Down | 1542 | 1.65E-13 |
| LOC_Os02g56460 | dehydrogenase, putative, expressed | Down | 1443 | 5.83E-06 |
| LOC_Os03g02550 | OsFBX76 - F-box domain containing protein, expressed | Down | 1736 | 4.91E-11 |
| LOC_Os05g31280 | GASR5 - Gibberellin-regulated GASA/GAST/Snakin family protein precursor, expressed | Down | 1086 | 4.69E-08 |
| LOC_Os08g36910 | alpha-amylase precursor, putative, expressed | Down | 2770 | 9.00E-16 |
| LOC_Os06g03520 | DUF581 domain containing protein, expressed | Down | 1010 | 0.000787 |
| LOC_Os11g13570 | gibberellin receptor GID1L2, putative, expressed | Down | 1522 | 4.23E-09 |
| LOC_Os03g49440 | phosphatase, putative, expressed | Down | 1225 | 1.42E-08 |
| LOC_Os02g25780 | expressed protein | Down | 1092 | 2.04E-05 |
| LOC_Os10g13700 | phosphoenolpyruvate carboxykinase, putative, expressed | Down | 2850 | 2.21E-09 |
| LOC_Os01g55240 | gibberellin 2-beta-dioxygenase, putative, expressed | Down | 1751 | 1.73E-06 |
| LOC_Os10g24954 | ulp1 protease family, C-terminal catalytic domain containing protein | Down | 2955 | 0.000667 |
| LOC_Os01g12110 | expressed protein | Down | 927 | 9.70E-06 |
| LOC_Os01g70080 | NB-ARC domain containing protein, expressed | Down | 3053 | 3.04E-05 |
| LOC_Os06g41030 | DUF1680 domain containing protein, putative, expressed | Down | 3020 | 2.51E-62 |
| LOC_Os07g09630 | oxidoreductase, putative, expressed | Down | 1179 | 5.35E-05 |
| LOC_Os07g47620 | universal stress protein domain containing protein, putative, expressed | Down | 2004 | 1.54E-30 |
| LOC_Os02g15280 | VQ domain containing protein, putative, expressed | Down | 468 | 1.60E-14 |
| LOC_Os03g04070 | no apical meristem protein, putative, expressed | Down | 1300 | 3.20E-06 |
| LOC_Os10g07998 | nodulin, putative, expressed | Down | 1372 | 2.55E-07 |
| LOC_Os02g39850 | transferase family protein, putative, expressed | Down | 1865 | 9.42E-06 |
| LOC_Os06g07600 | uncharacterized glycosyltransferase, putative, expressed | Down | 2952 | 1.07E-14 |
| LOC_Os03g12510 | non-symbiotic hemoglobin 2, putative, expressed | Down | 803 | 2.23E-11 |
| LOC_Os11g10510 | dehydrogenase, putative, expressed | Down | 1832 | 3.22E-16 |
| LOC_Os09g28420 | alpha-amylase precursor, putative, expressed | Down | 1547 | 0.000577 |
| LOC_Os02g37300 | heavy metal associated domain containing protein, expressed | Down | 806 | 2.08E-07 |
| LOC_Os06g05000 | early nodulin 93 ENOD93 protein, putative, expressed | Down | 778 | 3.32E-33 |
| LOC_Os12g40180 | expressed protein | Down | 625 | 1.38E-07 |
| LOC_Os03g55776 | expressed protein | Down | 849 | 5.93E-10 |
| LOC_Os09g31040 | EF hand family protein, putative, expressed | Down | 970 | 7.25E-06 |
| LOC_Os05g39320 | thiamine pyrophosphate enzyme, C-terminal TPP binding domain containing protein, expressed | Down | 1818 | 3.67E-14 |
| LOC_Os01g17170 | magnesium-protoporphyrin IX monomethyl ester cyclase,chloroplast precursor, putative, expressed | Down | 2335 | 1.51E-07 |
| LOC_Os06g45140 | bZIP transcription factor domain containing protein, expressed | Down | 1876 | 1.30E-19 |
| LOC_Os06g05020 | early nodulin 93 ENOD93 protein, putative, expressed | Down | 789 | 1.45E-98 |
| LOC_Os11g40590 | DUF1399 containing protein, putative, expressed | Down | 3972 | 1.19E-44 |
| LOC_Os05g44060 | expressed protein | Down | 846 | 2.16E-05 |
| LOC_Os11g07960 | transferase family protein, putative, expressed | Down | 1496 | 1.71E-11 |
| LOC_Os10g41550 | beta-amylase, putative, expressed | Down | 2130 | 7.02E-37 |
| LOC_Os01g69870 | expressed protein | Down | 981 | 3.36E-09 |
| LOC_Os06g43600 | LTPL129 - Protease inhibitor/seed storage/LTP family protein precursor, expressed | Down | 1657 | 7.94E-24 |
| LOC_Os05g49060 | uncharacterized protein ycf23, putative, expressed | Down | 1325 | 3.44E-05 |
| LOC_Os06g05010 | early nodulin 93 ENOD93 protein, putative, expressed | Down | 727 | 2.07E-40 |
| LOC_Os03g47530 | glycosyl transferase 8 domain containing protein, putative, expressed | Down | 1578 | 7.37E-17 |
| LOC_Os05g25770 | OsWRKY45 - Superfamily of TFs having WRKY and zinc finger domains, expressed | Down | 1528 | 6.99E-11 |
| LOC_Os07g44140 | cytochrome P450 72A1, putative, expressed | Down | 2285 | 2.42E-12 |
| LOC_Os05g35500 | MYB family transcription factor, putative, expressed | Down | 1195 | 4.75E-06 |
| LOC_Os02g03580 | transcription factor, putative, expressed | Down | 2059 | 1.89E-09 |
| LOC_Os02g22020 | MYB family transcription factor, putative, expressed | Down | 1711 | 1.54E-11 |
| LOC_Os05g39310 | thiamine pyrophosphate enzyme, C-terminal TPP binding domain containing protein | Down | 1818 | 1.83E-23 |
| LOC_Os06g46284 | glycosyl hydrolase, family 31, putative, expressed | Down | 3459 | 2.61E-26 |
| LOC_Os03g51390 | expressed protein | Down | 1166 | 0.000971 |
| LOC_Os02g52150 | heat shock 22 kDa protein, mitochondrial precursor, putative, expressed | Down | 1757 | 1.13E-25 |
| LOC_Os09g37080 | expressed protein | Down | 1261 | 3.81E-11 |
| LOC_Os03g11734 | MATE efflux protein, putative, expressed | Down | 2536 | 0.00022 |
| LOC_Os01g52660 | POEI51 - Pollen Ole e I allergen and extensin family protein precursor, expressed | Down | 1079 | 2.00E-08 |
| LOC_Os10g14020 | TPD1, putative, expressed | Down | 980 | 3.20E-08 |
| LOC_Os02g32580 | expressed protein | Down | 1101 | 1.62E-06 |
| LOC_Os03g55590 | MYB family transcription factor, putative, expressed | Down | 2367 | 1.28E-16 |
| LOC_Os04g16450 | aquaporin protein, putative, expressed | Down | 1629 | 4.60E-55 |
| LOC_Os03g29250 | SPX domain-containing protein, putative, expressed | Down | 1018 | 0.000205 |
| LOC_Os01g63690 | hs1, putative, expressed | Down | 1998 | 1.09E-18 |
| LOC_Os01g50420 | STE_MEKK_ste11_MAP3K.7 - STE kinases include homologs to sterile 7, sterile 11 and sterile 20 from yeast, expressed | Down | 1646 | 2.79E-11 |
| LOC_Os11g02080 | expressed protein | Down | 1139 | 9.03E-89 |
| LOC_Os03g06580 | MTN26L2 - MtN26 family protein precursor, expressed | Down | 1365 | 7.19E-05 |
| LOC_Os10g32050 | ankyrin repeat domain containing protein, expressed | Down | 933 | 0.000838 |
| LOC_Os12g02240 | hypothetical protein | Down | 300 | 3.66E-06 |
| LOC_Os05g46760 | STE_MEKK_ste11_MAP3K.19 - STE kinases include homologs to sterile 7, sterile 11 and sterile 20 from yeast, expressed | Down | 1419 | 0.000189 |
| LOC_Os03g57310 | syntaxin, putative, expressed | Down | 1577 | 0.000189 |
| LOC_Os10g31850 | RING finger and CHY zinc finger domain-containing protein 1, putative, expressed | Down | 1309 | 2.14E-32 |
| LOC_Os09g15480 | Ser/Thr-rich protein T10 in DGCR region, putative, expressed | Down | 1194 | 0.000494 |
| LOC_Os11g02240 | CAMK_KIN1/SNF1/Nim1_like.4 - CAMK includes calcium/calmodulin depedent protein kinases, expressed | Down | 2121 | 1.27E-35 |
| LOC_Os01g74370 | domain of unknown function DUF966 domain containing protein, expressed | Down | 1501 | 1.16E-14 |
| LOC_Os10g26700 | YGL010w, putative, expressed | Down | 1006 | 4.43E-07 |
| LOC_Os01g61080 | OsWRKY24 - Superfamily of TFs having WRKY and zinc finger domains, expressed | Down | 2161 | 1.19E-13 |
| LOC_Os03g52680 | expressed protein | Down | 1184 | 1.79E-06 |
| LOC_Os01g11340 | cytochrome P450, putative, expressed | Down | 2024 | 1.80E-06 |
| LOC_Os09g39440 | inosine-uridine preferring nucleoside hydrolase family protein, putative, expressed | Down | 1533 | 1.85E-13 |
| LOC_Os02g41904 | DEF7 - Defensin and Defensin-like DEFL family | Down | 638 | 6.85E-65 |
| LOC_Os07g37454 | urate anion exchanger, putative, expressed | Down | 1978 | 0.000263 |
| LOC_Os06g22060 | pyrophosphate--fructose 6-phosphate 1-phosphotransferase subunit alpha, putative, expressed | Down | 2676 | 0.000263 |
| LOC_Os02g32814 | heavy metal-associated domain containing protein, expressed | Down | 1179 | 7.89E-31 |
| LOC_Os10g40360 | proline oxidase, mitochondrial precursor, putative, expressed | Down | 2075 | 6.17E-07 |
| LOC_Os06g36560 | inositol oxygenase, putative, expressed | Down | 1797 | 6.30E-62 |
| LOC_Os04g02530 | expressed protein | Down | 2899 | 3.90E-24 |
| LOC_Os03g13300 | glutamate decarboxylase, putative, expressed | Down | 2149 | 3.55E-09 |
| LOC_Os03g05334 | expressed protein | Down | 656 | 7.09E-06 |
| LOC_Os03g06705 | 3-ketoacyl-CoA synthase, putative, expressed | Down | 1409 | 5.32E-05 |
| LOC_Os03g45250 | 2-aminoethanethiol dioxygenase, putative, expressed | Down | 1523 | 5.32E-05 |
| LOC_Os04g08350 | cysteine synthase, chloroplast/chromoplast precursor, putative, expressed | Down | 1624 | 8.82E-34 |
| LOC_Os04g47330 | rho-GTPase-activating protein-related, putative, expressed | Down | 913 | 1.91E-16 |
| LOC_Os03g58650 | expressed protein | Down | 1226 | 3.74E-06 |
| LOC_Os07g05040 | cadmium-induced protein, putative, expressed | Down | 982 | 2.79E-05 |
| LOC_Os10g25000 | expressed protein | Down | 992 | 0.000922 |
| LOC_Os07g47790 | AP2 domain containing protein, expressed | Down | 1000 | 2.66E-12 |
| LOC_Os01g04920 | glycosyl transferase, group 1 domain containing protein, expressed | Down | 1891 | 4.09E-10 |
| LOC_Os08g03420 | kelch repeat protein, putative, expressed | Down | 2060 | 9.28E-17 |
| LOC_Os08g37660 | plastocyanin-like domain containing protein, putative, expressed | Down | 1120 | 1.43E-27 |
| LOC_Os01g06560 | transcription factor HBP-1b, putative, expressed | Down | 1743 | 1.74E-14 |
| LOC_Os04g56230 | polyprenyl synthetase, putative, expressed | Down | 1722 | 8.56E-30 |
| LOC_Os05g07890 | embryo-specific 3, putative, expressed | Down | 781 | 4.42E-06 |
| LOC_Os02g32970 | hydrolase, alpha/beta fold family protein, putative, expressed | Down | 1552 | 1.69E-27 |
| LOC_Os04g23550 | basic helix-loop-helix family protein, putative, expressed | Down | 1400 | 3.48E-29 |
| LOC_Os11g02330 | LTPL22 - Protease inhibitor/seed storage/LTP family protein precursor, expressed | Down | 770 | 2.16E-22 |
| LOC_Os09g07154 | hypothetical protein | Down | 1110 | 1.37E-09 |
| LOC_Os04g55120 | jp18, putative, expressed | Down | 901 | 2.67E-07 |
| LOC_Os08g44850 | C2 domain containing protein, putative, expressed | Down | 1973 | 0.00071 |
| LOC_Os03g37490 | MATE efflux family protein, putative, expressed | Down | 2121 | 4.91E-05 |
| LOC_Os03g55240 | cytochrome P450, putative, expressed | Down | 2545 | 0.000244 |
| LOC_Os01g43480 | AAA-type ATPase family protein, putative, expressed | Down | 3476 | 8.00E-15 |
| LOC_Os07g06840 | gibberellin receptor GID1L2, putative, expressed | Down | 1447 | 1.58E-09 |
| LOC_Os03g04110 | lysM domain-containing GPI-anchored protein precursor, putative, expressed | Down | 1652 | 2.99E-06 |
| LOC_Os04g44510 | GEM, putative, expressed | Down | 1075 | 0.000125 |
| LOC_Os10g35460 | COBRA, putative, expressed | Down | 1617 | 1.18E-08 |
| LOC_Os05g43170 | calreticulin precursor protein, putative, expressed | Down | 1888 | 0.000212 |
| LOC_Os05g50750 | AAA family ATPase, putative, expressed | Down | 3085 | 2.02E-08 |
| LOC_Os06g03930 | cytochrome P450 86A1, putative, expressed | Down | 1997 | 2.00E-15 |
| LOC_Os02g31030 | glycerophosphoryl diester phosphodiesterase family protein, putative, expressed | Down | 1654 | 1.31E-19 |
| LOC_Os01g66830 | pectinacetylesterase domain containing protein, expressed | Down | 1242 | 1.05E-09 |
| LOC_Os04g42860 | GDSL-like lipase/acylhydrolase, putative, expressed | Down | 2004 | 0.000314 |
| LOC_Os09g26900 | ctr copper transporter family protein, putative, expressed | Down | 830 | 0.000537 |
| LOC_Os04g40730 | oxidoreductase, short chain dehydrogenase/reductase family, putative, expressed | Down | 1353 | 1.34E-08 |
| LOC_Os12g02040 | hypoxia-responsive family protein, putative, expressed | Down | 1026 | 5.37E-26 |
| LOC_Os05g33400 | basic 7S globulin precursor, putative, expressed | Down | 1482 | 1.65E-05 |
| LOC_Os09g28440 | AP2 domain containing protein, expressed | Down | 1100 | 2.79E-05 |
| LOC_Os07g41240 | cytochrome P450, putative, expressed | Down | 1945 | 0.000791 |
| LOC_Os02g50040 | endoglucanase, putative, expressed | Down | 2131 | 8.70E-08 |
| LOC_Os06g39140 | hemoglobin-like protein HbO, putative, expressed | Down | 965 | 2.42E-05 |
| LOC_Os11g37100 | expressed protein | Down | 2421 | 1.27E-08 |
| LOC_Os02g55910 | monogalactosyldiacylglycerol synthase, putative, expressed | Down | 2192 | 4.11E-11 |
| LOC_Os03g56590 | expressed protein | Down | 1853 | 7.68E-13 |
| LOC_Os04g19960 | retrotransposon protein, putative, unclassified, expressed | Down | 959 | 2.18E-06 |
| LOC_Os03g08220 | lipoxygenase protein, putative, expressed | Down | 3456 | 3.26E-09 |
| LOC_Os09g11440 | expressed protein | Down | 2132 | 3.53E-05 |
| LOC_Os11g25860 | protein Kinase, putative, expressed | Down | 3059 | 3.16E-06 |
| LOC_Os01g04670 | expressed protein | Down | 799 | 3.04E-05 |
| LOC_Os02g35900 | thioredoxin, putative, expressed | Down | 955 | 0.000175 |
| LOC_Os06g18010 | UDP-glucoronosyl and UDP-glucosyl transferase domain containing protein, expressed | Down | 1622 | 2.72E-06 |
| LOC_Os02g02400 | catalase isozyme A, putative, expressed | Down | 2421 | 7.96E-45 |
| LOC_Os06g42020 | CSLA9 - cellulose synthase-like family A, expressed | Down | 2147 | 0.00015 |
| LOC_Os11g16550 | uncharacterized protein ycf53, putative, expressed | Down | 1573 | 1.02E-07 |
| LOC_Os07g18750 | LTPL42 - Protease inhibitor/seed storage/LTP family protein precursor, expressed | Down | 748 | 1.13E-37 |
| LOC_Os09g27830 | OsPDIL2-3 protein disulfide isomerase PDIL2-3, expressed | Down | 1851 | 8.16E-16 |
| LOC_Os07g48280 | expressed protein | Down | 2726 | 7.27E-22 |
| LOC_Os03g53740 | expressed protein | Down | 901 | 1.92E-05 |
| LOC_Os08g44270 | vignain precursor, putative, expressed | Down | 1458 | 6.44E-05 |
| LOC_Os01g19820 | universal stress protein domain containing protein, putative, expressed | Down | 1080 | 3.22E-09 |
| LOC_Os08g33100 | core histone H2A/H2B/H3/H4, putative, expressed | Down | 697 | 1.26E-07 |
| LOC_Os05g45460 | POEI52 - Pollen Ole e I allergen and extensin family protein precursor, expressed | Down | 1079 | 6.89E-12 |
| LOC_Os06g48600 | macrophage migration inhibitory factor, putative, expressed | Down | 709 | 4.34E-07 |
| LOC_Os03g56070 | expressed protein | Down | 1858 | 2.16E-07 |
| LOC_Os01g21590 | homeodomain, putative, expressed | Down | 1644 | 2.48E-06 |
| LOC_Os09g28520 | expressed protein | Down | 1467 | 1.34E-08 |
| LOC_Os03g53020 | helix-loop-helix DNA-binding domain containing protein, expressed | Down | 1286 | 7.96E-05 |
| LOC_Os03g52320 | GRF-interacting factor 1, putative, expressed | Down | 1356 | 1.66E-08 |
| LOC_Os01g09220 | transposon protein, putative, CACTA, En/Spm sub-class, expressed | Down | 1834 | 8.28E-84 |
| LOC_Os03g59300 | expressed protein | Down | 1750 | 6.94E-16 |
| LOC_Os06g46340 | glycosyl hydrolase, family 31, putative, expressed | Down | 3094 | 3.43E-14 |
| LOC_Os02g52314 | BTB1 - Bric-a-Brac,Tramtrack, Broad Complex BTB domain, expressed | Down | 997 | 1.41E-08 |
| LOC_Os06g42660 | expressed protein | Down | 707 | 4.15E-11 |
| LOC_Os01g09030 | 2-aminoethanethiol dioxygenase, putative, expressed | Down | 3033 | 8.38E-20 |
| LOC_Os01g64256 | transposon protein, putative, Mutator sub-class, expressed | Down | 1998 | 0.000114 |
| LOC_Os01g04050 | BBTI12 - Bowman-Birk type bran trypsin inhibitor precursor, expressed | Down | 804 | 4.36E-06 |
| LOC_Os04g53950 | glycosyl hydrolases family 16 protein, protein, expressed | Down | 1481 | 1.22E-19 |
| LOC_Os02g45930 | expressed protein | Down | 1113 | 4.35E-15 |
| LOC_Os01g60860 | spotted leaf 11, putative, expressed | Down | 2494 | 0.000192 |
| LOC_Os09g36740 | tetratricopeptide repeat domain containing protein, expressed | Down | 1981 | 6.21E-06 |
| LOC_Os01g51230 | IQ calmodulin-binding motif domain containing protein, expressed | Down | 1676 | 2.00E-07 |
| LOC_Os06g09600 | expressed protein | Down | 710 | 5.28E-06 |
| LOC_Os03g58040 | glutamate dehydrogenase protein, putative, expressed | Down | 1698 | 5.68E-44 |
| LOC_Os12g02200 | CAMK_KIN1/SNF1/Nim1_like.6 - CAMK includes calcium/calmodulin depedent protein kinases, expressed | Down | 2193 | 3.31E-30 |
| LOC_Os04g47220 | aquaporin protein, putative, expressed | Down | 1343 | 9.85E-25 |
| LOC_Os01g63580 | glycerol-3-phosphate acyltransferase, putative, expressed | Down | 1715 | 4.99E-05 |
| LOC_Os02g41510 | MYB family transcription factor, putative, expressed | Down | 1042 | 9.92E-05 |
| LOC_Os03g04550 | expressed protein | Down | 1715 | 6.94E-07 |
| LOC_Os01g10860 | expressed protein | Down | 1111 | 0.000197 |
| LOC_Os01g52230 | phosphoethanolamine/phosphocholine phosphatase, putative, expressed | Down | 1217 | 2.90E-28 |
| LOC_Os02g44870 | dehydrin, putative, expressed | Down | 1371 | 2.46E-36 |
| LOC_Os08g31980 | trehalose-6-phosphate synthase, putative, expressed | Down | 3289 | 1.24E-07 |
| LOC_Os02g47130 | expressed protein | Down | 2513 | 3.65E-33 |
| LOC_Os07g32600 | glucan endo-1,3-beta-glucosidase precursor, putative, expressed | Down | 2186 | 2.18E-08 |
| LOC_Os07g32680 | retrotransposon protein, putative, unclassified, expressed | Down | 1424 | 1.46E-11 |
| LOC_Os03g20700 | magnesium-chelatase, putative, expressed | Down | 5655 | 9.91E-10 |
| LOC_Os03g60380 | cinnamoyl CoA reductase, putative, expressed | Down | 1797 | 1.77E-24 |
| LOC_Os04g22290 | hypothetical protein | Down | 711 | 2.16E-10 |
| LOC_Os09g16520 | cytochrome b5-like Heme/Steroid binding domain containing protein, expressed | Down | 935 | 3.01E-05 |
| LOC_Os10g31540 | glycine-rich cell wall structural protein 2 precursor, putative, expressed | Down | 899 | 3.54E-07 |
| LOC_Os11g02290 | expressed protein | Down | 873 | 0.000554 |
| LOC_Os06g14420 | hydrolase, NUDIX family, domain containing protein, expressed | Down | 1454 | 4.31E-10 |
| LOC_Os03g28330 | sucrose synthase, putative, expressed | Down | 3195 | 0 |
| LOC_Os10g29470 | dehydrogenase, putative, expressed | Down | 1564 | 1.81E-05 |
| LOC_Os11g05160 | DNA binding protein, putative, expressed | Down | 1640 | 8.44E-05 |
| LOC_Os03g45280 | dehydrin, putative, expressed | Down | 925 | 5.28E-41 |
| LOC_Os05g45030 | calcium homeostasis regulator CHoR1, putative, expressed | Down | 1300 | 7.07E-07 |
| LOC_Os08g45140 | OsGrx_S12 - glutaredoxin subgroup I, expressed | Down | 816 | 2.64E-08 |
| LOC_Os05g39230 | low photochemical bleaching 1 protein, putative, expressed | Down | 2984 | 2.79E-06 |
| LOC_Os05g33380 | fructose-bisphospate aldolase isozyme, putative, expressed | Down | 1442 | ######## |
| LOC_Os09g31490 | reductase, putative, expressed | Down | 1345 | 1.29E-05 |
| LOC_Os05g02300 | Core histone H2A/H2B/H3/H4 domain containing protein, putative, expressed | Down | 797 | 4.63E-06 |
| LOC_Os09g31080 | induced stolen tip protein TUB8, putative, expressed | Down | 1116 | 1.06E-97 |
| LOC_Os08g29669 | cytochrome b5-like Heme/Steroid binding domain containing protein, expressed | Down | 2000 | 9.11E-06 |
| LOC_Os07g46560 | seven in absentia protein family domain containing protein, expressed | Down | 1596 | 9.10E-06 |
| LOC_Os01g16250 | expressed protein | Down | 756 | 0.000466 |
| LOC_Os06g45090 | expressed protein | Down | 2200 | 4.49E-21 |
| LOC_Os03g20090 | MYB family transcription factor, putative, expressed | Down | 1758 | 8.15E-13 |
| LOC_Os06g47250 | DUF292 domain containing protein, expressed | Down | 2283 | 8.42E-05 |
| LOC_Os03g21640 | expressed protein | Down | 2753 | 3.01E-05 |
| LOC_Os08g32910 | expressed protein | Down | 2033 | 4.68E-09 |
| LOC_Os09g32640 | dehydrogenase, putative, expressed | Down | 1318 | 0.000328 |
| LOC_Os04g33820 | OsFBX132 - F-box domain containing protein, expressed | Down | 1299 | 1.74E-11 |
| LOC_Os03g14300 | THION29 - Plant thionin family protein precursor, expressed | Down | 889 | 1.25E-16 |
| LOC_Os09g23220 | glycosyl hydrolases family 16, putative, expressed | Down | 1467 | 9.91E-05 |
| LOC_Os10g40530 | LTPL146 - Protease inhibitor/seed storage/LTP family protein precursor, expressed | Down | 760 | 1.09E-08 |
| LOC_Os02g02410 | DnaK family protein, putative, expressed | Down | 2476 | 2.10E-79 |
| LOC_Os03g42420 | B3 DNA binding domain containing protein, expressed | Down | 1411 | 2.33E-06 |
| LOC_Os09g15700 | receptor-like protein kinase 5 precursor, putative, expressed | Down | 3194 | 5.19E-26 |
| LOC_Os06g10650 | tyrosine phosphatase family protein, putative, expressed | Down | 1214 | 0.000459 |
| LOC_Os02g09990 | expressed protein | Down | 1317 | 5.44E-09 |
| LOC_Os12g25630 | sulfite oxidase, putative, expressed | Down | 1666 | 3.52E-21 |
| LOC_Os02g49720 | aldehyde dehydrogenase, putative, expressed | Down | 2942 | 2.24E-12 |
| LOC_Os01g47760 | OsGrx_I1 - glutaredoxin subgroup III, expressed | Down | 981 | 1.02E-19 |
| LOC_Os01g46290 | lipase, putative, expressed | Down | 1650 | 9.63E-10 |
| LOC_Os03g01270 | expansin precursor, putative, expressed | Down | 1418 | 8.02E-10 |
| LOC_Os05g36280 | histone H3, putative, expressed | Down | 761 | 1.16E-24 |
| LOC_Os11g31980 | OsSCP63 - Putative Serine Carboxypeptidase homologue, expressed | Down | 2049 | 8.51E-08 |
| LOC_Os10g35580 | ATEB1A-like microtubule associated protein, putative, expressed | Down | 1383 | 5.70E-07 |
| LOC_Os12g05120 | receptor kinase, putative, expressed | Down | 2800 | 3.75E-06 |
| LOC_Os02g15810 | HMG1/2, putative, expressed | Down | 1899 | 1.32E-32 |
| LOC_Os12g12880 | expressed protein | Down | 1198 | 4.00E-07 |
| LOC_Os01g70740 | expressed protein | Down | 1692 | 5.73E-05 |
| LOC_Os08g05530 | LSM domain containing protein, expressed | Down | 336 | 0.000449 |
| LOC_Os09g32988 | POEI18 - Pollen Ole e I allergen and extensin family protein precursor, expressed | Down | 1524 | 6.13E-06 |
| LOC_Os01g09790 | IQ calmodulin-binding motif domain containing protein, expressed | Down | 1843 | 4.81E-05 |
| LOC_Os07g41200 | expressed protein | Down | 3291 | 1.27E-15 |
| LOC_Os04g10460 | amidase, putative, expressed | Down | 1842 | 0.000881 |
| LOC_Os10g42660 | expressed protein | Down | 1371 | 2.83E-05 |
| LOC_Os09g27820 | 1-aminocyclopropane-1-carboxylate oxidase protein, putative, expressed | Down | 1462 | 1.06E-09 |
| LOC_Os04g56240 | lipase, putative, expressed | Down | 2019 | 3.53E-19 |
| LOC_Os04g47250 | cytochrome P450, putative, expressed | Down | 2343 | 7.95E-05 |
| LOC_Os08g10510 | aminotransferase, putative, expressed | Down | 1955 | 3.59E-17 |
| LOC_Os07g44180 | OsRCI2-10 - Hydrophobic protein LTI6A, expressed | Down | 941 | 7.04E-21 |
| LOC_Os07g22580 | rhoGAP domain containing protein, expressed | Down | 1758 | 0.000619 |
| LOC_Os10g38470 | glutathione S-transferase, putative, expressed | Down | 1126 | 0.000187 |
| LOC_Os06g30370 | osMFT1 MFT-Like1 homologous to Mother of FT and TFL1 gene; contains Pfam profile PF01161: Phosphatidylethanolamine-binding protein, expressed | Down | 826 | 1.13E-10 |
| LOC_Os02g09960 | LYK8, putative, expressed | Down | 2372 | 0.000518 |
| LOC_Os07g46480 | eukaryotic aspartyl protease domain containing protein, expressed | Down | 1703 | 3.27E-08 |
| LOC_Os09g30190 | receptor-like protein kinase 2 precursor, putative, expressed | Down | 3700 | 2.45E-11 |
| LOC_Os06g22960 | aquaporin protein, putative, expressed | Down | 1358 | 2.03E-96 |
| LOC_Os02g53000 | lysM domain-containing GPI-anchored protein precursor, putative, expressed | Down | 2608 | 5.59E-11 |
| LOC_Os02g47180 | WD repeat-containing protein, putative, expressed | Down | 2655 | 9.47E-32 |
| LOC_Os02g53620 | nuclear transcription factor Y subunit, putative, expressed | Down | 1159 | 2.97E-06 |
| LOC_Os06g38294 | peptide transporter PTR2, putative, expressed | Down | 2290 | 9.45E-14 |
| LOC_Os04g50700 | pathogenesis-related Bet v I family protein, putative, expressed | Down | 665 | 7.40E-07 |
| LOC_Os05g09500 | hexokinase, putative, expressed | Down | 1878 | 7.67E-05 |
| LOC_Os05g34170 | tubulin/FtsZ domain containing protein, putative, expressed | Down | 1894 | 5.40E-11 |
| LOC_Os07g36500 | Core histone H2A/H2B/H3/H4 domain containing protein, putative, expressed | Down | 655 | 0.000256 |
| LOC_Os07g48510 | thioredoxin, putative, expressed | Down | 1477 | 1.89E-25 |
| LOC_Os03g07810 | expressed protein | Down | 1621 | 0.000851 |
| LOC_Os03g39020 | Kinesin motor domain domain containing protein, expressed | Down | 3842 | 3.44E-21 |
| LOC_Os01g68950 | ubiquitin family domain containing protein, expressed | Down | 303 | 7.83E-35 |
| LOC_Os02g06360 | uncharacterized protein CPn_0526/CP_0226/CPj0526/CpB0547, putative, expressed | Down | 1481 | 0.000712 |
| LOC_Os01g68650 | plant-specific domain TIGR01615 family protein, expressed | Down | 1123 | 6.29E-08 |
| LOC_Os02g47310 | Cyclopropane-fatty-acyl-phospholipid synthase, putative, expressed | Down | 1535 | 0.000596 |
| LOC_Os04g51460 | glycosyl hydrolases family 16, putative, expressed | Down | 1199 | 1.68E-17 |
| LOC_Os02g52210 | zinc finger, C3HC4 type domain containing protein, expressed | Down | 2241 | 0.000291 |
| LOC_Os11g02350 | LTPL25 - Protease inhibitor/seed storage/LTP family protein precursor, expressed | Down | 926 | 1.02E-34 |
| LOC_Os10g14180 | expressed protein | Down | 7259 | 1.70E-08 |
| LOC_Os08g41440 | UDP-glucuronate 4-epimerase, putative, expressed | Down | 2014 | 3.39E-11 |
| LOC_Os01g04409 | OsWAK1 - OsWAK receptor-like cytoplasmic kinase OsWAK-RLCK, expressed | Down | 2460 | 0.000816 |
| LOC_Os01g68660 | cysteine proteinase inhibitor precursor protein, putative, expressed | Down | 786 | 2.81E-32 |
| LOC_Os06g06460 | histone H3, putative, expressed | Down | 1030 | 5.79E-08 |
| LOC_Os01g53710 | dual specificity protein phosphatase, putative, expressed | Down | 1591 | 9.91E-09 |
| LOC_Os08g17160 | plastocyanin-like domain containing protein, putative, expressed | Down | 1016 | 2.11E-05 |
| LOC_Os05g06440 | dnaJ homolog subfamily B member 11 precursor, putative, expressed | Down | 1660 | 2.11E-05 |
| LOC_Os12g04440 | 2-isopropylmalate synthase B, putative, expressed | Down | 2565 | 0.0001 |
| LOC_Os03g55120 | plastocyanin-like domain containing protein, putative, expressed | Down | 985 | 3.38E-09 |
| LOC_Os02g56920 | WAX2, putative, expressed | Down | 2338 | 6.61E-08 |
| LOC_Os06g49760 | invertase/pectin methylesterase inhibitor family protein, putative, expressed | Down | 963 | 3.51E-10 |
| LOC_Os03g45760 | expressed protein | Down | 1489 | 1.84E-06 |
| LOC_Os03g02780 | Core histone H2A/H2B/H3/H4 domain containing protein, putative, expressed | Down | 732 | 6.91E-15 |
| LOC_Os01g64640 | histone H3, putative, expressed | Down | 819 | 5.77E-15 |
| LOC_Os03g16860 | DnaK family protein, putative, expressed | Down | 2268 | 8.61E-06 |
| LOC_Os05g27090 | basic helix-loop-helix domain containing protein, expressed | Down | 663 | 4.91E-05 |
| LOC_Os05g33690 | receptor-like protein kinase precursor, putative, expressed | Down | 2438 | 2.66E-08 |
| LOC_Os06g49100 | retrotransposon protein, putative, unclassified, expressed | Down | 1935 | 4.38E-07 |
| LOC_Os02g42330 | nitrilase, putative, expressed | Down | 1747 | 2.55E-07 |
| LOC_Os12g13570 | MYB family transcription factor, putative, expressed | Down | 3383 | 0.00065 |
| LOC_Os04g43230 | expressed protein | Down | 1778 | 6.88E-06 |
| LOC_Os05g33460 | mTERF domain containing protein, expressed | Down | 1629 | 0.000454 |
| LOC_Os04g50960 | expressed protein | Down | 2865 | 0.000378 |
| LOC_Os07g33240 | endoribonuclease, putative, expressed | Down | 1033 | 1.77E-35 |
| LOC_Os04g47580 | cyclin, putative, expressed | Down | 1816 | 1.13E-11 |
| LOC_Os07g42324 | hypothetical protein | Down | 1233 | 6.79E-08 |
| LOC_Os06g44080 | ubiquitin family protein, putative, expressed | Down | 834 | 7.76E-06 |
| LOC_Os01g70560 | expressed protein | Down | 1425 | 7.59E-05 |
| LOC_Os07g05820 | hydroxyacid oxidase 1, putative, expressed | Down | 1779 | 2.73E-07 |
| LOC_Os06g05740 | expressed protein | Down | 1345 | 5.29E-05 |
| LOC_Os10g39140 | flavonol synthase/flavanone 3-hydroxylase, putative, expressed | Down | 2269 | 8.98E-51 |
| LOC_Os08g13920 | glycosyl hydrolases family 16, putative, expressed | Down | 1436 | 7.84E-10 |
| LOC_Os11g31900 | acyl carrier protein, putative, expressed | Down | 1072 | 2.59E-07 |
| LOC_Os01g74040 | zinc finger, RING-type, putative, expressed | Down | 1526 | 4.90E-10 |
| LOC_Os06g39370 | OsFBK16 - F-box domain and kelch repeat containing protein, expressed | Down | 1707 | 0.000694 |
| LOC_Os06g14460 | chromosome condensation protein like, putative, expressed | Down | 1520 | 0.000337 |
| LOC_Os02g45780 | zinc finger, C3HC4 type domain containing protein, expressed | Down | 1274 | 0.000337 |
| LOC_Os08g17680 | stromal cell-derived factor 2-like protein precursor, putative, expressed | Down | 1270 | 0.000337 |
| LOC_Os04g31924 | nodulin, putative, expressed | Down | 2759 | 8.06E-05 |
| LOC_Os04g32540 | OsSCP24 - Putative Serine Carboxypeptidase homologue, expressed | Down | 2085 | 4.70E-05 |
| LOC_Os06g03710 | DELLA protein SLR1, putative, expressed | Down | 3102 | 3.92E-05 |
| LOC_Os05g02060 | mitochondrial import inner membrane translocase subunit Tim17, putative, expressed | Down | 870 | 2.81E-60 |
| LOC_Os03g02290 | kinesin motor domain containing protein, putative, expressed | Down | 2188 | 2.25E-06 |
| LOC_Os02g56540 | kinesin motor domain containing protein, putative, expressed | Down | 2970 | 2.26E-06 |
| LOC_Os03g36560 | peroxidase precursor, putative, expressed | Down | 1497 | 0.000931 |
| LOC_Os02g47350 | oxidoreductase, short chain dehydrogenase/reductase family, putative, expressed | Down | 1518 | 6.30E-05 |
| LOC_Os01g01870 | helix-loop-helix DNA-binding domain containing protein, expressed | Down | 1347 | 4.41E-05 |
| LOC_Os02g19820 | nodulin MtN3 family protein, putative, expressed | Down | 1530 | 5.35E-45 |
| LOC_Os02g06340 | EH domain-containing protein 1, putative, expressed | Down | 1915 | 1.32E-10 |
| LOC_Os04g56400 | glutamine synthetase, catalytic domain containing protein, expressed | Down | 1843 | 3.00E-06 |
| LOC_Os04g31524 | expressed protein | Down | 1234 | 4.97E-07 |
| LOC_Os01g53990 | pectinesterase, putative, expressed | Down | 1842 | 1.92E-14 |
| LOC_Os03g12900 | squalene monooxygenase, putative, expressed | Down | 3148 | 1.60E-14 |
| LOC_Os04g28420 | peptidyl-prolyl isomerase, putative, expressed | Down | 2218 | 3.34E-16 |
| LOC_Os06g43044 | GDSL-like lipase/acylhydrolase, putative, expressed | Down | 1555 | 9.76E-06 |
| LOC_Os08g42470 | BEE 1, putative, expressed | Down | 1366 | 1.97E-06 |
| LOC_Os04g17660 | rhodanese-like domain containing protein, putative, expressed | Down | 2130 | 0.000508 |
| LOC_Os03g46920 | expressed protein | Down | 2854 | 1.02E-08 |
| LOC_Os04g49370 | expressed protein | Down | 1437 | 2.61E-25 |
| LOC_Os03g17200 | plant-specific domain TIGR01589 family protein | Down | 237 | 1.64E-08 |
| LOC_Os03g05750 | heavy-metal-associated domain-containing protein, putative, expressed | Down | 1561 | 7.42E-07 |
| LOC_Os09g12600 | phosphate/phosphate translocator, putative, expressed | Down | 1842 | 3.91E-85 |
| LOC_Os12g29760 | oxidoreductase, aldo/keto reductase family protein, putative, expressed | Down | 1342 | 2.21E-06 |
| LOC_Os07g36590 | serine/threonine-protein kinase receptor precursor, putative, expressed | Down | 2911 | 3.13E-06 |
| LOC_Os01g60740 | LTPL16 - Protease inhibitor/seed storage/LTP family protein precursor, expressed | Down | 1060 | 9.99E-89 |
| LOC_Os10g33040 | receptor-like protein kinase precursor, putative, expressed | Down | 5076 | 0.000812 |
| LOC_Os07g14270 | calreticulin precursor protein, putative, expressed | Down | 2177 | ######## |
| LOC_Os03g06670 | Core histone H2A/H2B/H3/H4 domain containing protein, putative, expressed | Down | 880 | 2.81E-09 |
| LOC_Os07g41280 | 6-phosphogluconolactonase, putative, expressed | Down | 1843 | 4.02E-09 |
| LOC_Os06g05860 | 6-phosphofructokinase, putative, expressed | Down | 2043 | 1.41E-18 |
| LOC_Os12g02290 | LTPL23 - Protease inhibitor/seed storage/LTP family protein precursor, expressed | Down | 1036 | 1.59E-17 |
| LOC_Os01g08150 | expressed protein | Down | 2809 | 1.06E-07 |
| LOC_Os01g22954 | serine carboxypeptidase, putative, expressed | Down | 1984 | 4.29E-05 |
| LOC_Os04g15800 | expressed protein | Down | 1490 | 5.12E-05 |
| LOC_Os04g40950 | glyceraldehyde-3-phosphate dehydrogenase, putative, expressed | Down | 1571 | 0 |
| LOC_Os03g46640 | deoxyuridine 5-triphosphate nucleotidohydrolase, putative, expressed | Down | 1033 | 0.000125 |
| LOC_Os06g14620 | ribonucleoside-diphosphate reductase small chain, putative, expressed | Down | 1651 | 1.59E-06 |
| LOC_Os02g46910 | glycosyl hydrolases family 16, putative, expressed | Down | 2457 | 0.000181 |
| LOC_Os08g41280 | membrane protein, putative, expressed | Down | 1635 | 0.000215 |
| LOC_Os01g56880 | purple acid phosphatase precursor, putative, expressed | Down | 2227 | 3.88E-06 |
| LOC_Os03g03630 | expressed protein | Down | 955 | 0.000902 |
| LOC_Os03g17960 | expressed protein | Down | 2141 | 2.31E-05 |
| LOC_Os01g52500 | NADP-dependent malic enzyme, putative, expressed | Down | 3179 | 1.24E-06 |
| LOC_Os10g39750 | inducer of CBF expression 2, putative, expressed | Down | 1480 | 4.75E-05 |
| LOC_Os01g43140 | lipase, putative, expressed | Down | 2312 | 4.75E-05 |
| LOC_Os11g20790 | adenylate kinase, putative, expressed | Down | 1153 | 1.06E-15 |
| LOC_Os05g03620 | TKL_IRAK_CR4L.4 - The CR4L subfamily has homology with Crinkly4, expressed | Down | 2731 | 2.53E-06 |
| LOC_Os09g04160 | expressed protein | Down | 2681 | 9.49E-09 |
| LOC_Os03g05710 | acetyltransferase, GNAT family, putative, expressed | Down | 1385 | 0.00014 |
| LOC_Os08g43730 | DUF630/DUF632 domains containing protein, putative, expressed | Down | 3239 | 0.000167 |
| LOC_Os10g28230 | Core histone H2A/H2B/H3/H4 domain containing protein, putative, expressed | Down | 1005 | 2.74E-11 |
| LOC_Os02g47840 | universal stress protein domain containing protein, putative, expressed | Down | 843 | 8.72E-06 |
| LOC_Os05g02070 | expressed protein | Down | 890 | 0 |
| LOC_Os06g45990 | patellin-5, putative, expressed | Down | 3390 | 1.26E-05 |
| LOC_Os03g52650 | syntaxin-related protein, putative, expressed | Down | 1249 | 2.08E-09 |
| LOC_Os05g15510 | cellulase, putative, expressed | Down | 1980 | 0.000284 |
| LOC_Os04g41900 | expressed protein | Down | 1119 | 4.27E-09 |
| LOC_Os03g08470 | AP2 domain containing protein, expressed | Down | 1763 | 0.000488 |
| LOC_Os01g62420 | triosephosphate isomerase, cytosolic, putative, expressed | Down | 1420 | 4.01E-18 |
| LOC_Os05g01970 | NAD dependent epimerase/dehydratase family protein, putative, expressed | Down | 1609 | 6.29E-05 |
| LOC_Os02g45940 | Core histone H2A/H2B/H3/H4 domain containing protein, putative, expressed | Down | 844 | 7.54E-05 |
| LOC_Os05g41390 | cyclin, putative, expressed | Down | 2132 | 6.48E-08 |
| LOC_Os02g07870 | ATP synthase, putative, expressed | Down | 2500 | 0.000108 |
| LOC_Os08g01760 | dehydrogenase, putative, expressed | Down | 1754 | 2.38E-18 |
